# A comparative study of two-way and offline coupled WRF v3.4 and CMAQ v5.0.2 over the contiguous US: performance evaluation and impacts of chemistry–meteorology feedbacks on air quality

**DOI:** 10.5194/gmd-14-7189-2021

**Published:** 2021-11-26

**Authors:** Kai Wang, Yang Zhang, Shaocai Yu, David C. Wong, Jonathan Pleim, Rohit Mathur, James T. Kelly, Michelle Bell

**Affiliations:** 1 Department of Civil and Environmental Engineering, Northeastern University, Boston, MA 02115, USA; 2 Key Laboratory of Environmental Remediation and Ecological Health, Ministry of Education; Research Center for Air Pollution and Health, College of Environment and Resource Sciences, Zhejiang University, Hangzhou, Zhejiang 310058, P.R. China; 3 Center for Environmental Measurement and Modeling, U.S. EPA, Research Triangle Park, NC 27711, USA; 4 Office of Air Quality Planning and Standards, U.S. EPA, Research Triangle Park, NC 27711, USA; 5 School of Forestry & Environmental Studies, Yale University, New Haven, CT 06511, USA

## Abstract

The two-way coupled Weather Research and Forecasting and Community Multiscale Air Quality (WRF-CMAQ) model has been developed to more realistically represent the atmosphere by accounting for complex chemistry–meteorology feedbacks. In this study, we present a comparative analysis of two-way (with consideration of both aerosol direct and indirect effects) and offline coupled WRF v3.4 and CMAQ v5.0.2 over the contiguous US. Long-term (5 years from 2008 to 2012) simulations using WRF-CMAQ with both offline and two-way coupling modes are carried out with anthropogenic emissions based on multiple years of the U.S. National Emission Inventory and chemical initial and boundary conditions derived from an advanced Earth system model (i.e., a modified version of the Community Earth System Model/Community Atmospheric Model). The comprehensive model evaluations show that both two-way WRF-CMAQ and WRF-only simulations perform well for major meteorological variables such as temperature at 2 m, relative humidity at 2 m, wind speed at 10 m, precipitation (except for against the National Climatic Data Center data), and shortwave and longwave radiation. Both two-way and offline CMAQ also show good performance for ozone (O_3_) and fine particulate matter (PM_2.5_). Due to the consideration of aerosol direct and indirect effects, two-way WRF-CMAQ shows improved performance over offline coupled WRF and CMAQ in terms of spatiotemporal distributions and statistics, especially for radiation, cloud forcing, O_3_, sulfate, nitrate, ammonium, elemental carbon, tropospheric O_3_ residual, and column nitrogen dioxide (NO_2_). For example, the mean biases have been reduced by more than 10 W m^−2^ for shortwave radiation and cloud radiative forcing and by more than 2 ppb for max 8 h O_3_. However, relatively large biases still exist for cloud predictions, some PM_2.5_ species, and PM_10_ that warrant follow-up studies to better understand those issues. The impacts of chemistry–meteorological feedbacks are found to play important roles in affecting regional air quality in the US by reducing domain-average concentrations of carbon monoxide (CO), O_3_, nitrogen oxide (NO_*x*_), volatile organic compounds (VOCs), and PM_2.5_ by 3.1% (up to 27.8%), 4.2% (up to 16.2%), 6.6% (up to 50.9%), 5.8% (up to 46.6%), and 8.6% (up to 49.1%), respectively, mainly due to reduced radiation, temperature, and wind speed. The overall performance of the two-way coupled WRF-CMAQ model achieved in this work is generally good or satisfactory and the improved performance for two-way coupled WRF-CMAQ should be considered along with other factors in developing future model applications to inform policy making.

## 1 Introduction

The Community Multiscale Air Quality (CMAQ) modeling system developed by the U.S. Environmental Protection Agency (EPA) (Byun and Schere, 2006; Scheffe et al., 2016; San Joaquin Valley Air Pollution Control District, 2018; Pye et al., 2020; U.S. EPA, 2020) has been extensively used by both the scientific community and governmental agencies over various geographical regions and under different meteorological and air pollution conditions to address major key air quality issues such as atmospheric ozone (O_3_), acid rain, regional haze, and trans-boundary or long-range transport of air pollutants during the past decades over North America (Zhang et al., 2009a, b; Wang and Zhang, 2012; Hogrefe et al., 2015), Asia (Wang et al., 2009, 2012; Liu et al., 2010; Zheng et al., 2015; Li et al., 2017; Xing et al., 2017; Yu et al., 2018; Mehmood et al., 2020), and Europe (Kukkonen et al., 2012; Mathur et al., 2017; Solazzo et al., 2017). The CMAQ model is traditionally driven offline by the three-dimensional meteorology fields generated separately from other meteorological models, such as the Weather Research and Forecasting (WRF) model, and the dynamic feedbacks of chemistry predictions on meteorology are neglected. However, more recently (IPCC, 2018), chemistry–meteorology feedbacks have been found to play important roles in affecting both global and regional climate change and air quality (Jacobson et al., 1996; Mathur et al., 1998; Ghan et al., 2001; Zhang, 2008; Zhang et al., 2010, 2015a, b, 2017; Grell and Baklanov, 2011; Wong et al., 2012; Baklanov et al., 2014; Yu et al., 2014; Gan et al., 2015a; Wang et al., 2015a; Xing et al., 2015a, b; Yahya et al., 2015a, b; Hong et al., 2017; Jung et al., 2019). Feedbacks of aerosols on radiative transfer through aerosol–radiation interactions (i.e., aerosol direct forcing) and aerosol–cloud interactions (i.e., aerosol indirect forcing) are especially important (Zhang, 2008; Zhang et al., 2015a, b; Baklanov et al., 2014; Wang et al., 2015a; Yahya et al., 2015a, b). Recognizing this importance, as well as the recent advances in knowledge on chemistry–meteorology interactions and computational resources, the U.S. EPA developed a two-way coupled WRF-CMAQ model that accounts for the aerosol direct effect alone (Wong et al., 2012). This version of CMAQ has been applied for both regional and hemispheric studies (Wang et al., 2014; Hogrefe et al., 2015; Xing et al., 2016, 2017; Hong et al., 2017, 2020; Sekiguchi et al., 2018; Yoo et al., 2019). For example, Xing et al. (2016) showed that aerosol direct feedbacks may further improve air quality resulting from emission controls in the US and also indicated that coupled models are key tools for quantifying such feedbacks. Reduction in atmospheric ventilation resulting from aerosol-induced surface cooling can exacerbate ground level air pollution. Hong et al. (2017) estimated an increase by 4.8%–9.5% in concentrations of major air pollutants over China in winter due to incorporation of such effects. Xing et al. (2017) reported that the aerosol direct effects could reduce daily max 1 h O_3_ by up to 39 μg m^−3^ over China in January through reducing solar radiation and photolysis rates. Hong et al. (2020) found that the benefits of reduced pollutant emissions through weakening aerosol direct effects can largely offset the additional deaths caused by the warming effect of greenhouse gases over China. Some of those studies have also found that the missing aerosol indirect effects in WRF-CMAQ may introduce large model biases on their simulations of radiation and thus air quality (Wang et al., 2014; Sekiguchi et al., 2018; Yoo et al., 2019). There has been a growing awareness that both aerosol effects should be considered together to provide greater fidelity in coupling complex atmospheric processes among chemistry, aerosols, cloud, radiation, and precipitation (Grell and Baklanov, 2011). To address this issue and better represent the one-atmosphere modeling capability of CMAQ, Yu et al. (2014) further extended the two-way coupled WRF-CMAQ model by including aerosol indirect effects and improved WRF-CMAQ’s capability for predicting cloud and radiation variables.

Different from the traditional online integrated air quality models, such as the Gas, Aerosol, Transport, Radiation, General Circulation, and Mesoscale Meteorological (GATOR-GCMM) model (Jacobson, 2001), the WRF model coupled with chemistry (WRF/Chem; Grell et al., 2005), and the WRF model coupled with the Community Atmosphere Model version 5 (WRF-CAM5; Ma et al., 2013; Zhang et al., 2015a, b, 2017), in which atmospheric dynamics and chemistry are integrated and simulated altogether without an interface between meteorology and atmospheric chemistry (Zhang et al., 2013), two-way WRF-CMAQ (also referred to as the online access model) is created by combining existing meteorology (i.e., WRF) and atmospheric chemistry (i.e., CMAQ) models with an interactive interface (Yu et al., 2014). As pointed out by Yu et al. (2014), the main advantage of two-way CMAQ is to allow the existing numerical techniques to be used in both WRF and CMAQ to facilitate future independent development of both models while also maintaining CMAQ as a stand-alone model (the offline capability). In the past, a number of studies have compared and evaluated online vs. offline coupled model performance (Pleim et al., 2008; Matsui et al., 2009; Wilczak et al., 2009; Lin et al., 2010; Herwehe et al., 2011; Yu et al., 2011; Wong et al., 2012; Zhang et al., 2013, 2016a; Choi et al., 2019). However, due to the missing offline coupled mode or component for most online coupled models, many of those intercomparison studies are subject to some key limitations such as inconsistent model treatments in chemical options (Matsui et al., 2009; Lin et al., 2010; Zhang et al., 2013; Choi et al., 2019) or in both physical and chemical options (Wilczak et al., 2009; Herwehe et al., 2011; Zhang et al., 2016a), different domain projection methods or resolutions (Wilczak et al., 2009; Lin et al., 2010; Zhang et al., 2013), or disunified model inputs (Wilczak et al., 2009; Lin et al., 2010; Zhang et al., 2013). Due to the unique coupling approach, two-way WRF-CMAQ can be used to overcome those limitations and set up ideal intercomparisons between online and offline simulations using consistent model treatments (Pleim et al., 2008; Yu et al., 2011; Wong et al., 2012).

In this study, we provide a robust examination of model improvements by considering chemistry–meteorology feedbacks and their impacts on US air quality using the two-way WRF-CMAQ model (the same version as in Yu et al., 2014) with both aerosol direct and indirect effects. Long-term (5 years from 2008 to 2012) simulations using both two-way and offline coupled WRF and CMAQ models are carried out and compared (to the best of our knowledge) for the first time over the contiguous US (CONUS) with anthropogenic emissions based on multiple years of the U.S. National Emission Inventory (NEI) and chemical initial and boundary conditions (ICONs/BCONs) downscaled from the advanced Earth system model, i.e., an updated version of the Community Earth System Model/CAM5 (CESM/CAM5; He and Zhang, 2014; Glotfelty et al., 2017). Our objectives include (1) performing a comprehensive model evaluation for major meteorological variables and chemical species from this long-term application of the two-way coupled WRF-CMAQ and (2) conducting a comparative study of two-way and offline coupled WRF and CMAQ to examine the impacts of chemistry–meteorology interactions on US air quality.

Compared to previous studies in the literature, there are a few key features of this work. First, the intercomparisons between two-way (or online) and offline WRF-CMAQ are performed here using consistent model configurations including both physical and chemical options and inputs. Second, unlike a few previous intercomparison studies (Pleim et al., 2008; Yu et al., 2011; Wong et al., 2012) using two-way WRF-CMAQ with only aerosol direct effects for relatively short episodes, the model version in this work includes both aerosol direct and indirect effects and simulations are conducted for multiple years to provide more robust assessments. Third, compared to other studies (e.g., Yahya et al., 2015a, b; Choi et al., 2019) focusing on the impacts of chemistry–meteorology feedbacks on meteorology only or limited chemical species, this study performs comprehensive and extensive evaluation and comparison to demonstrate importance of chemistry–meteorology feedbacks on regional meteorology and air quality.

## 2 Model description, simulation setup, and evaluation protocols

Two sets of 5-year (i.e., 2008–2012) long-term simulations are conducted using the two-way coupled WRF v3.4-CMAQ v5.0.2 model with both aerosol direct and indirect effects and the sequentially offline coupled WRF v3.4 and CMAQ v5.0.2 model, respectively, over the CONUS with 36 km horizontal grid spacing. The vertical resolution for these simulations consists of 34 layers from the surface (~ 38 m) to 100 hPa (~ 15 km). The two-way coupled WRF-CMAQ includes estimations of aerosol optical properties based on prognostic aerosol size distributions and composition. These aerosol optical properties are then used to modulate the shortwave radiation budget estimated using the Rapid and accurate Radiative Transfer Model for General circulation (RRTMG) radiation scheme (Iacono et al., 2008) in WRF. Additionally, aerosol indirect effects, including the first (cloud albedo) and second (cloud lifetime) indirect aerosol forcing and the glaciation (ice- and mixed-phase cloud lifetime) indirect aerosol forcing are also modeled. More details on the model development of this version of WRF-CMAQ can be found in Yu et al. (2014). On the other hand, the WRF-only model calculates the radiation budgets by using prescribed aerosol optical properties such as aerosol optical depth, single-scattering albedo and asymmetry parameters and cloud formation by assuming default droplet number concentration and fixed cloud effective radius, which may not be representative for the large regions with complex air pollution conditions. Both the two-way and offline coupled WRF-CMAQ use the same model configurations as shown in Table S1 in the Supplement, except that prognostic aerosol impacts on radiation and clouds are fully treated in two-way WRF-CMAQ. The physics options include the RRTMG shortwave and longwave radiation schemes, the Asymmetric Convective Model (ACM2) planetary boundary layer (PBL) scheme (Pleim, 2007), the Pleim-Xiu (PX) land-surface scheme (Xiu and Pleim, 2001), the Morrison two-moment microphysics scheme (Morrison et al., 2009), and version 2 of the Kain–Fritsch (KF2) cumulus scheme (Kain, 2004). The chemical options include the Carbon Bond 2005 (CB05) chemical mechanism (Yarwood et al., 2005) with additional chloride chemistry (Sarwar et al., 2008), the sixth-generation CMAQ aerosol module (AERO6) (Appel et al., 2013), and CMAQ’s aqueous-phase chemistry (AQCHEM). In addition, the time steps of dynamics and radiation for two-way WRF-CMAQ are set as 1 and 15 min, respectively, and the call frequency for CMAQ in the two-way coupled model is set to be 5 min.

The meteorological ICONs/BCONs are generated from the National Centers for Environmental Prediction Final Analysis (NCEP-FNL) datasets, and the chemical ICONs/BCONs are downscaled from a modified version of CESMv1.2.2/CAM5 (He and Zhang, 2014; Glotfelty et al., 2017). The chemical ICONs/BCONs generated from CESM simulations consider the year-to-year variation. The CESM simulations have been comprehensively evaluated against surface data, remote sensing (including satellite) data, and reanalysis data for major meteorological and chemical variables over Europe, Asia, North America, and the globe. The results are also compared with other existing global model results and show generally satisfactory or superior performance. The anthropogenic emissions are based on two versions of NEI. NEI 2008 and NEI 2011 are used to cover the 5-year period, i.e., NEI 2008 for 2008–2010 and NEI 2011 for 2011–2012. Biogenic emissions are calculated online using the Biogenic Emissions Inventory System (BEIS) v3 (Schwede et al., 2005). The sea salt and dust emissions are also generated online by CMAQ’s inline modules (Zender et al., 2003; Zhang et al., 2005). Two-way coupled WRF-CMAQ simulations are reinitialized every 5 d for meteorology fields only. We have conducted sensitivity simulations in the past (Wang et al., 2021) and found that a 5 d reinitialization frequency is more suitable to improve the overall simulation quality while preserving chemistry–meteorology feedbacks. The WRF-only simulations apply the same reinitialization method to make sure any deviation between two simulations is determined more by the feedback processes.

The model evaluation in this work mainly focuses on the long-term climatological type of performance in representative seasons (i.e., winter and summer) by comparing 5-year average spatially and temporally matched model predictions of major surface meteorological and radiation-cloud variables and surface and column chemical species against various surface and satellite observations and reanalysis data (the 5-year annual results can be found in the Supplement). A brief inter-annual comparison between observations and two-way CMAQ simulations is also performed for selected major meteorological and chemical variables to examine the model’s capability in reproducing the year-to-year variations of those variables. The surface meteorological data include temperature at 2 m (T2), relative humidity at 2 m (RH2), wind speed at 10 m (WS10), and wind direction at 10 m (WD10) from the National Climatic Data Center (NCDC), and precipitation from the NCDC, the National Acid Deposition Program (NADP), the Global Precipitation Climatology Project (GPCP), the Parameter-elevation Regressions on Independent Slopes Model (PRISM), and the Tropical Rainfall Measuring Mission Multisatellite Precipitation Analysis (TMPA). The radiation and cloud data include downward shortwave radiation at the ground surface (SWDOWN), net shortwave radiation at the ground surface (GSW), downward longwave radiation at the ground surface (GLW), outgoing longwave radiation at the top of the atmosphere (OLR), and shortwave and longwave cloud forcing (SWCF and LWCF) from the Clouds and the Earth’s Radiant Energy System (CERES); aerosol optical depth (AOD), cloud fraction (CF), cloud water path (CWP), and cloud optical thickness (COT) from the MODerate resolution Imaging Spectroradiometer (MODIS); and cloud droplet number concentration (CDNC) derived based on MODIS data by Bennartz (2007). The chemical data include surface O_3_ from the Aerometric Information Retrieval System-Air Quality Subsystem (AIRS-AQS) and the Clean Air Status and Trends Network (CASTNET); surface particulate matter with 2.5 μm or less (PM_2.5_) and its constituents including sulfate (${\text{SO}}_{4}^{2-}$), nitrate (${\text{NO}}_{3}^{-}$), ammonium (${\text{NH}}_{4}^{+}$), elemental carbon (EC), organic carbon (OC), and total carbon (TC = EC+OC) from the Interagency Monitoring of Protected Visual Environments (IMPROVE) and the Chemical Speciation Network (CSN); surface particulate matter with diameters of 10 μm or less (PM_10_) from the AQS; and column abundance variables such as column carbon monoxide (CO) from the Measurements of Pollution in the Troposphere (MOPITT), tropospheric ozone residual (TOR) from the Ozone Monitoring Instrument (OMI), and column nitrogen dioxide (NO_2_) and formaldehyde (HCHO) from the Scanning Imaging Absorption Spectrometer for Atmospheric Chartography (SCIAMACHY).

The satellite datasets used in this study are all level 3 gridded monthly averaged data with various resolutions (i.e., 0.25° for OMI and PRISM; 0.5° for SCIAMACHY; 1° for CERES, GPCP, MODIS, and MOPITT). For the calculation of model performance statistics, the satellite data with different resolutions are mapped to CMAQ’s Lambert conformal conic projection using bi-linear interpolation in the NCAR command language. CMAQ model outputs at approximate time of the satellite overpass are paired with the satellite retrievals to facilitate a consistent comparison. Note that only those grid points with valid satellite observations are considered when paring model results with observations, and the averaging kernels are not considered when analyzing the column CO and NO_2_ results, which may introduce some uncertainties (Wang et al., 2015b). Modeled CDNC is calculated as the average value of the layer of low-level warm clouds between 950 and 850 hPa as suggested by Bennartz (2007). Following the approach of Wielicki et al. (1996), the SWCF and LWCF are calculated as the difference between the clear-sky and the all-sky reflected radiation at the top of atmosphere for both simulations and observations.

The statistical performance evaluation follows a protocol similar to that of Zhang et al. (2006, 2009a) and Yahya et al. (2016) and uses well-accepted statistical measures such as correlation coefficient (*R*), mean bias (MB), root-mean-square error (RMSE), normalized mean biases (NMB), and normalized mean error (NME) (S. Yu et al., 2006). Because of different sampling protocols among monitoring networks, the evaluation is conducted separately for individual networks for the same simulated variables and species.

## 3 Comprehensive model evaluation of two-way WRF-CMAQ

### 3.1 Meteorological evaluation

#### 3.1.1 Surface meteorological variables

**Figure 1.**
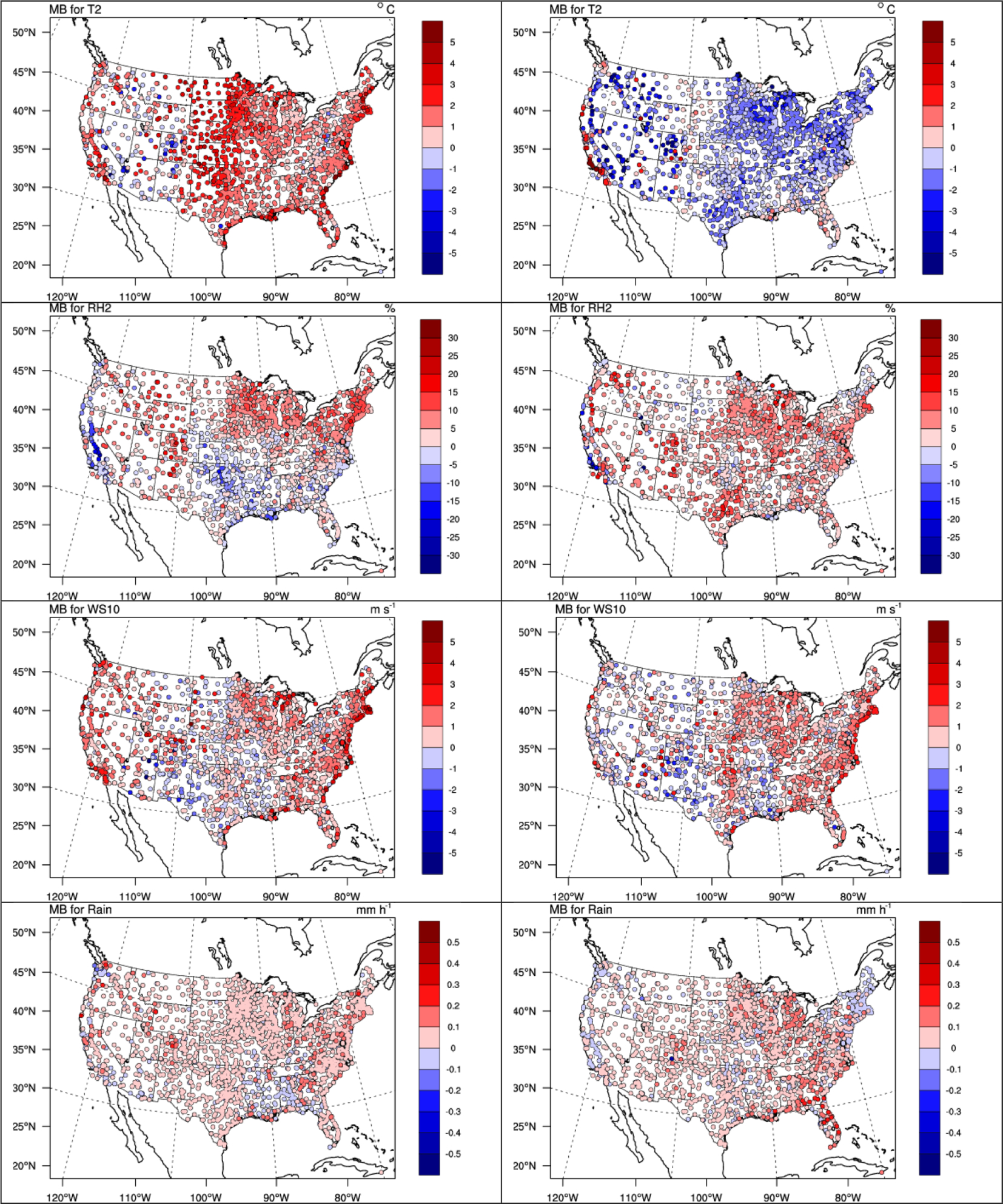
Spatial distributions of 5-year average MBs for 2 m temperature (T2), 2 m relative humidity (RH2), 10 m wind speed (WS10), and hourly precipitation from NCDC for two-way WRF-CMAQ in winter (left column) and summer (right column) 2008–2012.

Figure 1 shows the spatial distribution of 5-year average MBs for T2, RH2, WS10, and hourly precipitation from two-way WRF-CMAQ against the NCDC data in winter and summer 2008–2012, and Tables 1 and 2 summarize the statistics for the same variables. Most variables except for precipitation show overall moderate to good spatial performance, with many sites showing MBs within ±1.0°C for T2, ±10% for RH2, ±1 m s^−1^ for WS10, and ±0.2 mm h^−1^ for precipitation in both seasons. WRF-CMAQ tends to overpredict T2 (i.e., warm bias) over widespread areas of domain, especially along the US Atlantic coast, the eastern and southeastern US, the central US, and the US Pacific coast in winter and underpredict T2 (i.e., cold bias) over the eastern US, the central US, and mountainous US in summer, which leads to an overall small warm bias in the whole year (see Fig. S1 in the Supplement). Similar warm biases of T2 in winter have been previously reported by Cohen et al. (2015) and are found to be associated with the relatively deep PBL depth using the non-local ACM2 PBL scheme. The relatively larger warm and cold biases over coastal and mountainous areas are likely due to the coarse grid spacing of 36 km that cannot resolve the complex topography well (Yahya et al., 2016). Compared to many previous WRF studies (Wang et al., 2012; Brunner et al., 2015; Yahya et al., 2016), which typically show cold T2 biases, the overall small warm biases in this study can be attributed to the soil moisture nudging technique used in the PX land surface scheme (Pleim and Gilliam, 2009). The spatial patterns of MBs for RH2 show a general anti-correlation compared to T2 (i.e., RH2 is overpredicted where T2 is underpredicted and vice versa) due to the way RH2 is calculated based on T2. The spatial distribution of MBs for WS10 also shows dominant overpredictions in both winter and summer, especially along coastlines, indicating the prescribed sea surface temperature might not be sufficient to resolve the air–sea interactions. Systematic overpredictions of hourly precipitation against NCDC data in both seasons are found to be mainly caused by low non-convective precipitation events and can be attributed to the Morrison microphysics scheme (Yahya et al., 2016).

**Figure 2.**
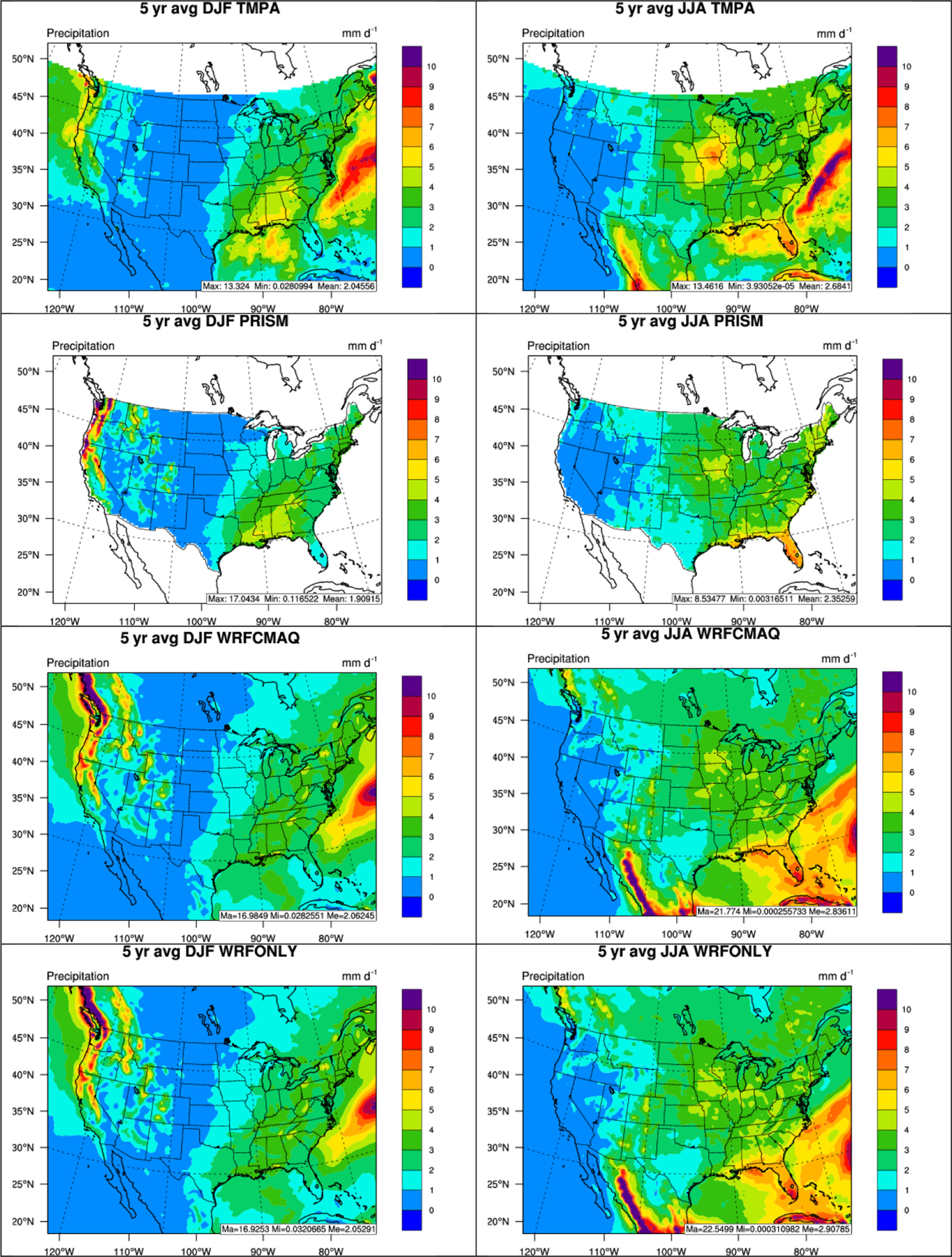
Spatial distributions of 5-year average of daily precipitation from TMPA, PRISM, two-way WRF-CMAQ, and WRF-only (from top to bottom) in winter (left column) and summer (right column) 2008–2012.

The precipitation performance is further examined by comparing WRF-CMAQ with TMPA and PRISM as shown in Fig. 2. The spatial distribution of precipitation is well simulated by WRF-CMAQ, especially over the CONUS, against observations by capturing the hot spots along the Pacific Northwest coast in winter and some areas over the central US and Florida in summer. Moderate overpredictions of precipitation against TMPA over the Atlantic Ocean and Gulf of Mexico in summer are also evident, possibly caused by overprediction of convective precipitation by the Kain–Fritsch scheme (Hong et al., 2017) over ocean. As shown in Tables 1 and 2, the domain-average seasonal statistics demonstrate good performance for all variables except for precipitation against NCDC in terms of MBs, NMBs, RMSE, and *R*s. For example, the MBs for T2, RH2, WS10, and precipitation are 1.1 °C, 2.2%, 0.57 m s^−1^, and 0.05–0.23 mm d^−1^ (except for 0.71 mm d^−1^ for NCDC) in winter and −1.1 °C, 3.7%, 0.38 m s^−1^, and 0.13–0.23 mm d^−1^ (except for 0.75 mm d^−1^ for NCDC) in summer, respectively, and *R*s for those variables are typically between 0.5–0.97, which are well within the performance benchmark values recommended by Zhang et al. (2013) and Emery et al. (2017).

**Figure 3.**
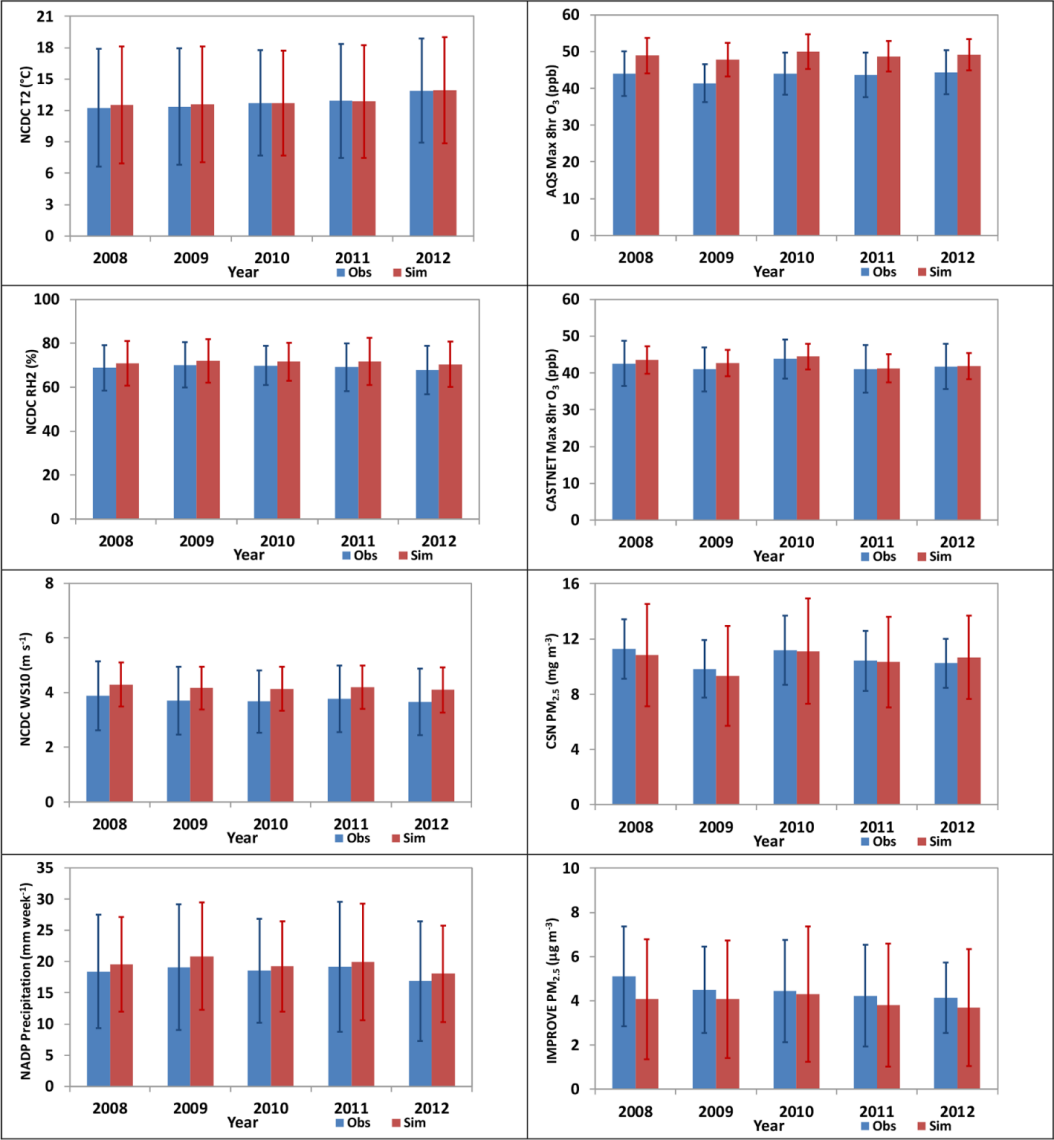
Bar charts for annual average observations and simulations (standard deviations are displayed as the error bars) from two-way WRF-CMAQ for major meteorological variables (left column) and chemical species (right column) in 2008–2012.

Figure 3 shows the bar charts of annual trends for T2, RH2, WS10, and precipitation in 2008–2012. Two-way WRF-CMAQ predicts the annual average T2 very well with MBs < 0.25 °C in all years. The simulation can also capture the increasing trend of T2 from 2008 to 2012 observed by NCDC. RH2 is consistently overpredicted by the two-way WRF-CMAQ in all years despite relatively low biases (MBs < 3%). Both observations and simulations show the lowest RH2 in 2012 and the highest in 2009. As also shown in Fig. 1, the model tends to systematically overpredict both WS10 and precipitation throughout all years as well. There are no clear trends (i.e., increasing or decreasing) for WS10 and precipitation between 2008 to 2012 from either observations or simulations. However two-way WRF-CMAQ is able to capture both the lowest wind speed and precipitation in 2012 and the highest wind speed in 2008 from observations. In general, the model performs very well in reproducing the year-to-year variation for the major meteorological variables between 2008 to 2012.

#### 3.1.2 Radiation and cloud variables

**Figure 4.**
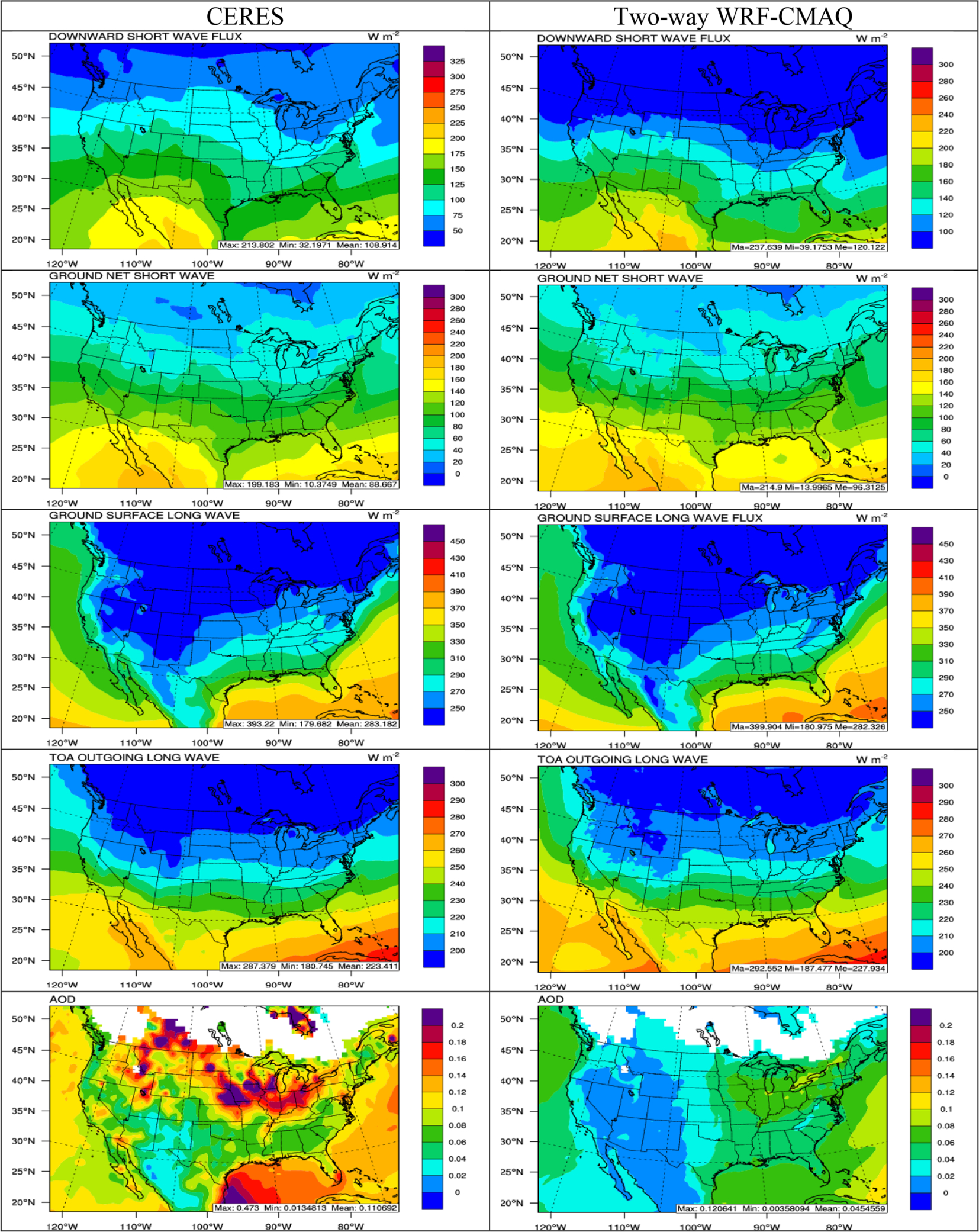
Spatial distribution of 5-year average major radiation variables (from top to bottom: SWDOWN, GSW, GLW, OLR, and AOD) between CERES observations (left column) vs. two-way WRF-CMAQ (right column) in winter 2008–2012.

**Figure 5.**
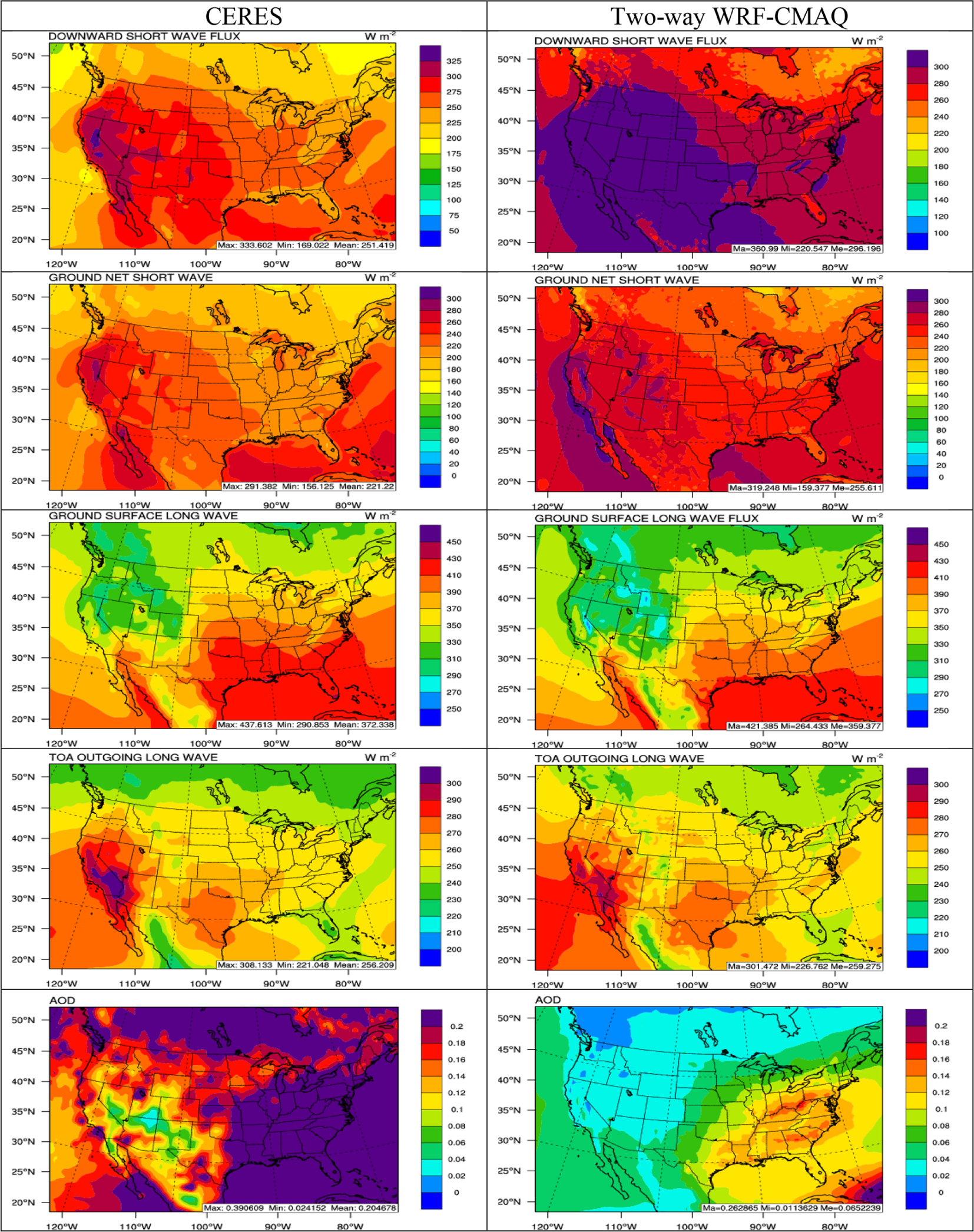
Spatial distribution of 5-year average major radiation variables (from top to bottom: SWDOWN, GSW, GLW, OLR, and AOD) between CERES observations (left column) vs. two-way WRF-CMAQ (right column) in summer 2008–2012.

Figures 4 and 5 compare the 5-year average spatial distribution of major radiation variables (i.e., SWDOWN, GSW, GLW, OLR, and AOD) based on the satellite retrievals and two-way WRF-CMAQ simulations in winter and summer 2008–2012, and Tables 1 and 2 summarize the domain-average model performance statistics. WRF-CMAQ predicts the longwave radiation variables GLW and OLR very well with domain-average NMBs of −0.3% and 1.8% in winter and −3.6% and 0.9% in summer, respectively, and *R*s of 0.96 to 0.99 for both. The shortwave radiation variables SWDOWN and GSW are slightly overpredicted on average with NMBs of 11.3% and 7.5% in winter and 17.1% and 15.1% in summer, respectively, and *R*s ranging from 0.75 to 0.99 for both. The simulations also reliably reproduce the spatial distribution of both longwave and shortwave radiation compared to observations in both seasons. The relatively large overpredictions for shortwave radiation especially in summer are very likely caused by the large underpredictions of aerosol direct radiative forcing reflected from the underpredictions of AOD (Fig. 5), as well as underprediction of indirect cloud radiative forcing (see Fig. 8). It has been reported that WRF v3.4 does not treat the subgrid cloud feedback to radiation, which could also contribute to the overpredictions in shortwave radiation, especially in summer (Alapaty et al., 2012; Hong et al., 2017). The model largely underpredicts the magnitude of AOD in both seasons (NMBs of −59.8% in winter and −67.8% in summer), while providing a reasonable representation of the spatial distribution of AOD over the US, with generally higher values over the Midwest in winter and over the eastern US in summer. The model also underpredicts the elevated AODs over oceans and the northern part of domain in both seasons. Similar AOD underpredictions have been reported in previous studies over the US using two-way coupled WRF-CMAQ (Gan et al., 2015a; Hogrefe et al., 2015; Xing et al., 2015a). The relatively large underpredictions of AOD may be caused by several factors. First, underprediction of PM_2.5_ concentrations, particularly ${\text{SO}}_{4}^{2-}$ in both seasons and OC in summer (Tables 3 and 4), can contribute significantly to the underprediction of AOD, especially over the eastern US. Second, the underestimation of dust emissions may contribute to missing hot spots from the model over arid areas in California and Arizona (Zender et al., 2003) and underestimates of sea salt emissions may lead to missing elevated AODs over oceans (Gan et al., 2015b). Third, challenges in adequately representing prescribed and wildfire emissions in the NEI (Kelly et al., 2019) may cause many missing hot spots over large areas of the Pacific Northwest, California, Canada, and the eastern US, especially in summer. Fourth, uncertainties in BCONs of PM_2.5_ concentrations may further contribute to underpredictions of AOD over oceans and the northern part of the domain. For example, Kaufman et al. (2001) found that the background AOD could reach 0.1 over the Pacific Northwest using Aerosol Robotic Network (AERONET) data. The AODs in the current simulation seem to be biased low (between 0.02–0.06 in both seasons over the Pacific Ocean) and indicate potential underpredictions of PM_2.5_ BCONs, especially in the free troposphere. Finally, there are uncertainties associated with MODIS retrievals. Remer et al. (2005) found that the uncertainty of level 3 MODIS monthly AODs can be up to ±0.05 ± 0.15 AOD over the land due to clouds and surface reflectance. More AOD data from other satellites or AERONET might be considered in the future work to provide more robust ensemble type of evaluation for AOD.

**Figure 6.**
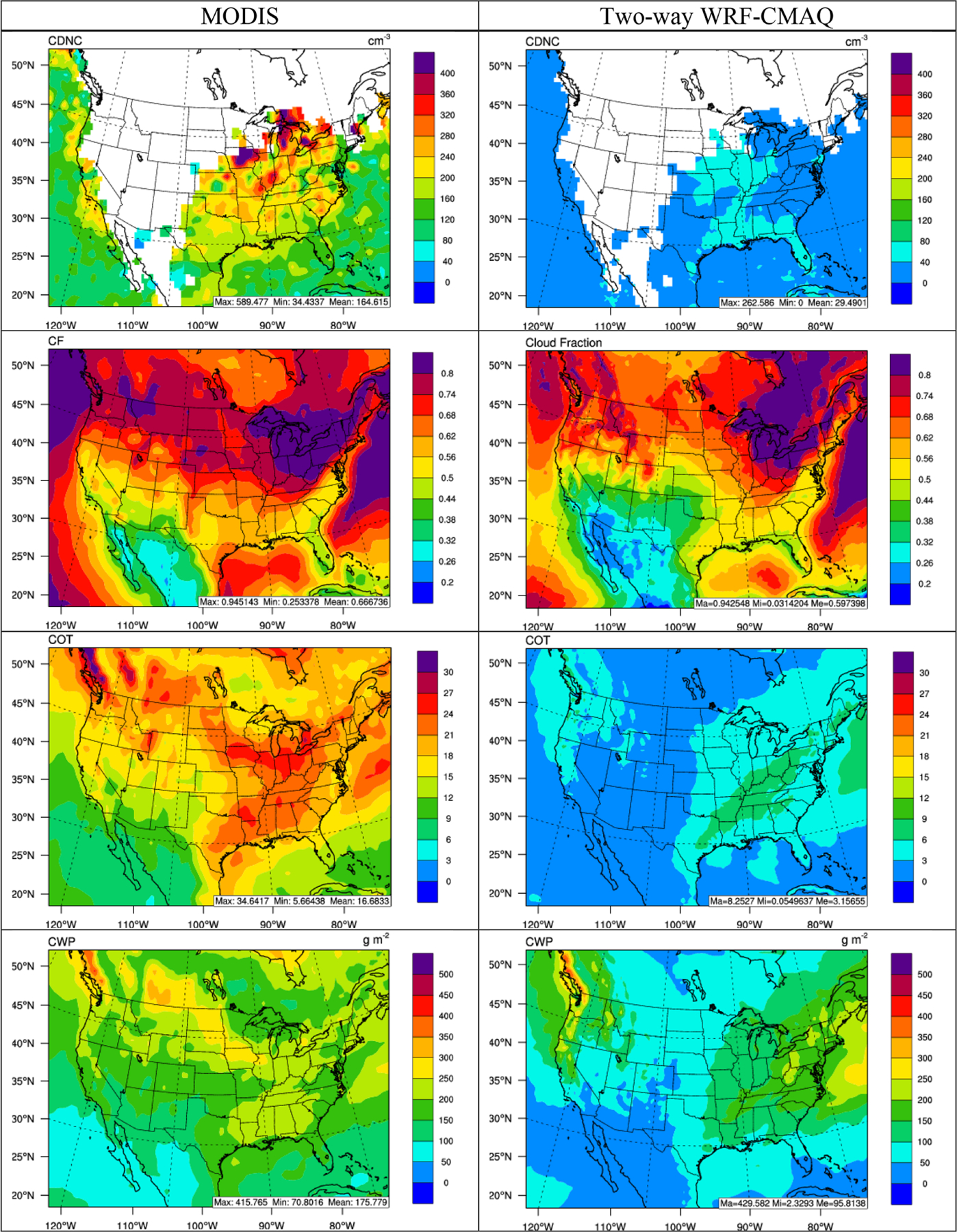
Spatial distribution of 5-year average major cloud variables (from top to bottom: CDNC, CF, COT, and CWP) between MODIS observations (left column) vs. two-way WRF-CMAQ (right column) in winter 2008–2012.

**Figure 7.**
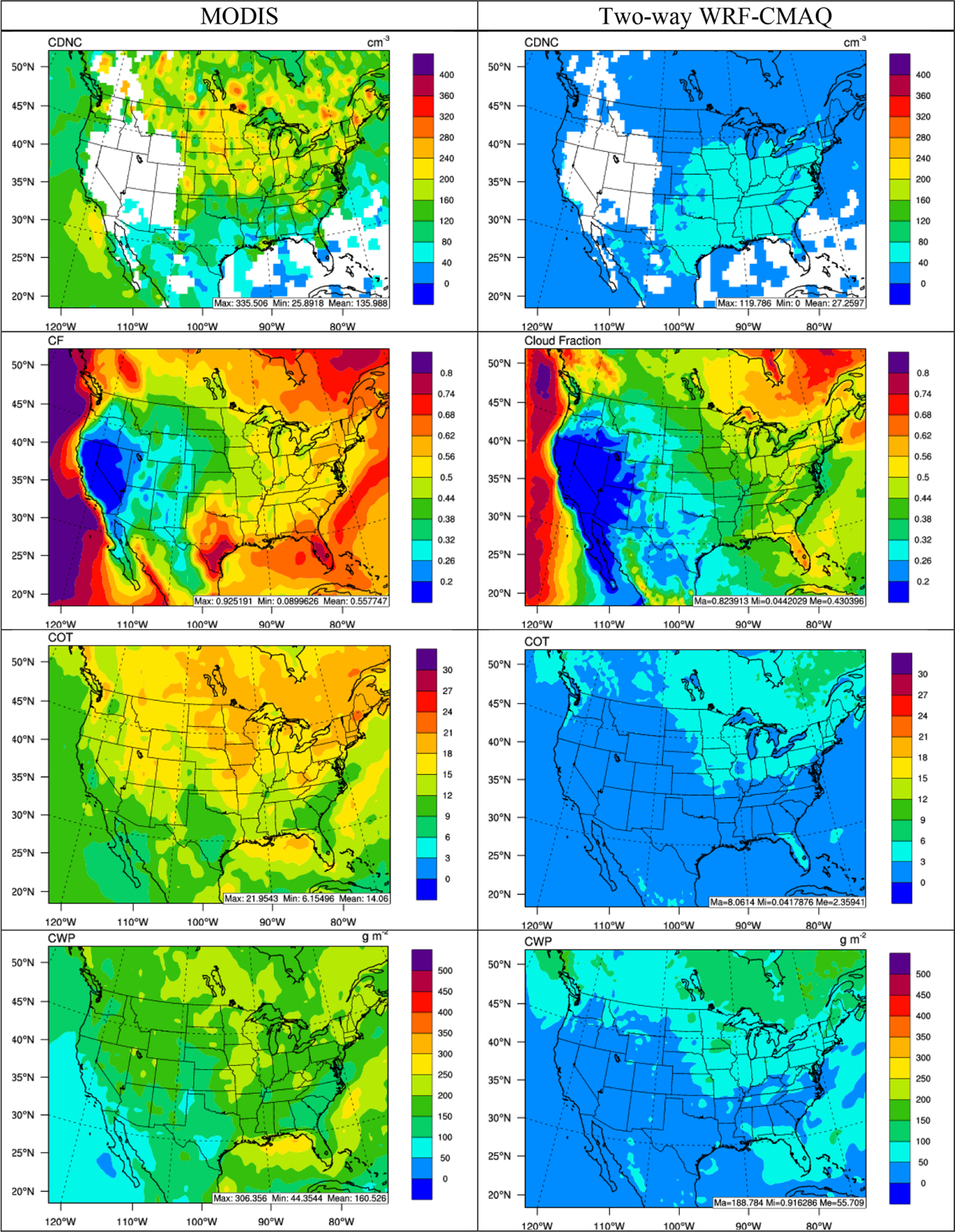
Spatial distribution of 5-year average major cloud variables (from top to bottom: CDNC, CF, COT, and CWP) between MODIS observations (left column) vs. two-way WRF-CMAQ (right column) in summer 2008–2012.

**Figure 8.**
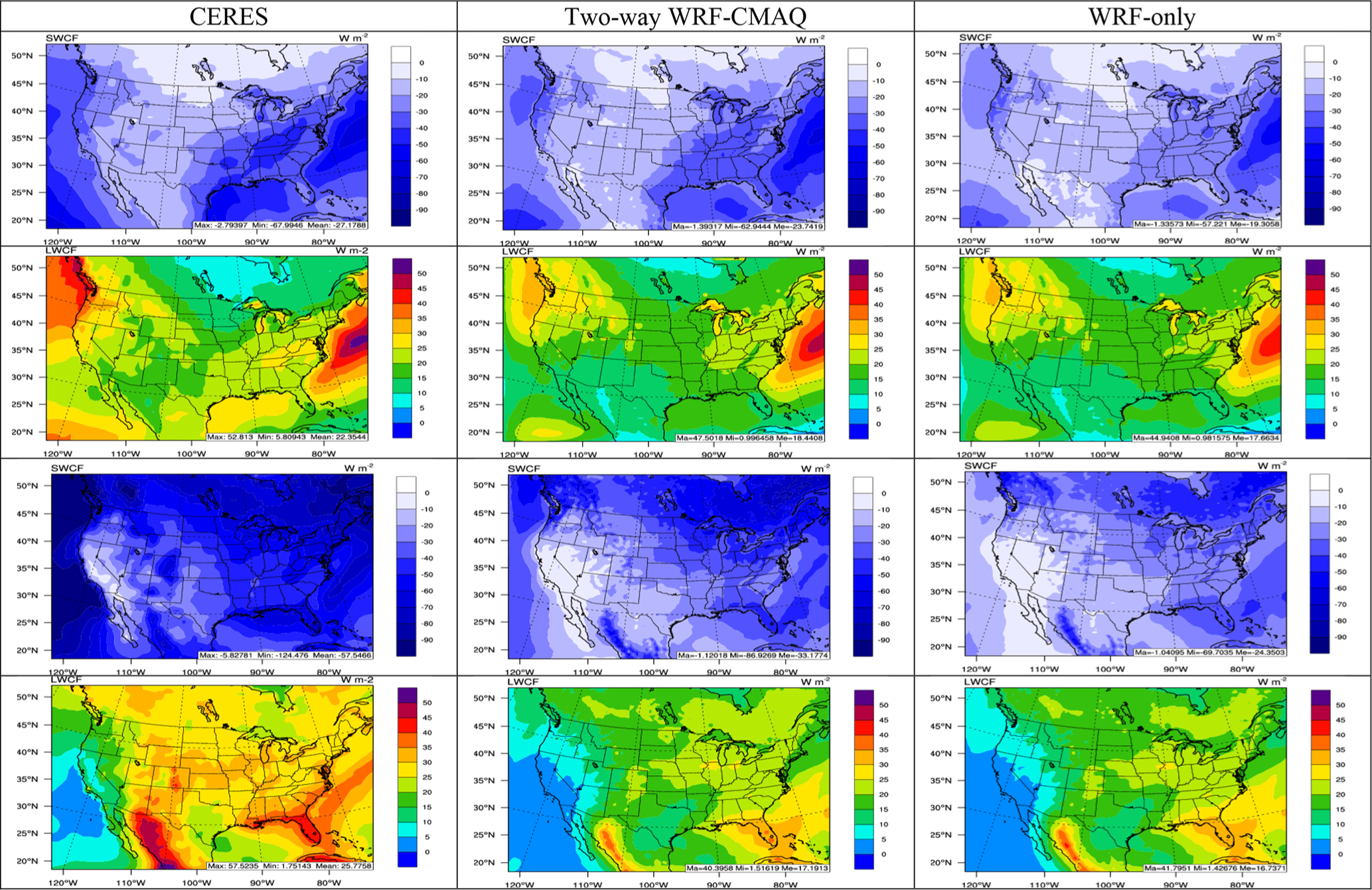
Spatial distribution of 5-year average SWCF in winter, LWCF in winter, SWCF in summer, and LWCF in summer (from top to bottom) between CERES observations (left column) vs. two-way WRF-CMAQ (center column) and WRF-only (right column) in 2008–2012.

Figures 6–8 compare the 5-year average spatial distribution of major cloud and cloud radiative variables for the satellite retrievals and two-way WRF-CMAQ simulations in winter and summer 2008–2012, and Tables 1 and 2 summarize the corresponding statistics. As shown in Figs. 6 and 7, WRF-CMAQ tends to largely underpredict CDNC, COT, and CWP in both seasons over most of the domain with the domain-average NMBs of −82.4%, −80.8%, and −45.3% in winter and −79.2%, −83.6%, and −66.3% in summer, respectively. Despite the large underprediction of those cloud variables, the spatial correlations are generally predicted well, especially for COT and CWP, with *R*s ranging from 0.63 to 0.74. Compared to the other cloud variables, CF is much better predicted, with an NMB of −10.4% and an *R* of 0.87 in winter and an NMB of −23.0% and an *R* of 0.81 in summer, which is consistent with the performance reported in Yu et al. (2014). The model can reproduce the high CFs over the northern and northeastern parts of domain, as well as over oceans, while capturing the low CFs over the mountainous and plateau regions in the US and Mexico, especially in winter. In addition to the underprediction of PM_2.5_ (thus underestimating CCN), the large underpredictions of cloud variables (especially CDNC and COT) can be attributed to uncertainties in aerosol microphysics schemes (Yahya et al., 2016), as well as missing aerosol indirect effects on subgrid convective clouds (Yu et al., 2014). Gantt et al. (2014) and Zhang et al. (2015b) also showed the aerosol activation scheme (i.e., Abdul-Razzak and Ghan, 2000) used in the current version of WRF-CMAQ may have underestimated CDNC and thus CWP and COT due to some missing processes such as insoluble aerosol adsorption and giant cloud condensation nuclei. Overall, the relatively poor model performance for cloud variables reflects current limitations in representing aerosol indirect effects and aerosol–cloud interactions in state-of-science online coupled models. Further model improvements that incorporate new knowledge from emerging studies should be conducted in the future.

As shown in Fig. 8, WRF-CMAQ predictions of SWCF and LWCF generally agree well with the satellite observations in both seasons. The model can capture the elevated SWCF and LWCF over the Atlantic Ocean and widespread areas over the eastern US in winter and those over the Pacific Northwest, northern part of the domain, and Atlantic Ocean in summer. The domain-average NMBs are −11.1% for SWCF and −15.1% for LWCF in winter and −41.3% for SWCF and −33.3% for LWCF in summer. The relatively larger biases in summer compared to winter are correlated with larger biases associated with radiation and cloud predictions potentially caused by larger underpredictions of aerosol predictions. As discussed earlier, the underpredictions of SWCF may partially contribute the overprediction of SWDOWN (more shortwave radiation reaching the ground), and those of LWCF may further lead to the overpredictions in OLR (more longwave radiation emitted into the space). The performance of SWCF and LWCF is consistent with the 12 km simulation reported in Yu et al. (2014) and even slightly better in terms of NMBs, which might be associated with the long-term vs. short-term simulations. It is also worth noting that SWCF (LWCF) is calculated as the difference between the clear-sky and all-sky shortwave (longwave) radiation at the top of the atmosphere, and thus performance for SWCF and LWCF depends on performance for both radiation and cloud properties. The generally better performance in terms of model bias for SWCF and LWCF compared to the cloud variables seems to be driven by the relatively good performance of shortwave and longwave radiation in the model.

### 3.2 Chemical evaluation

#### 3.2.1 O_3_

**Figure 9.**
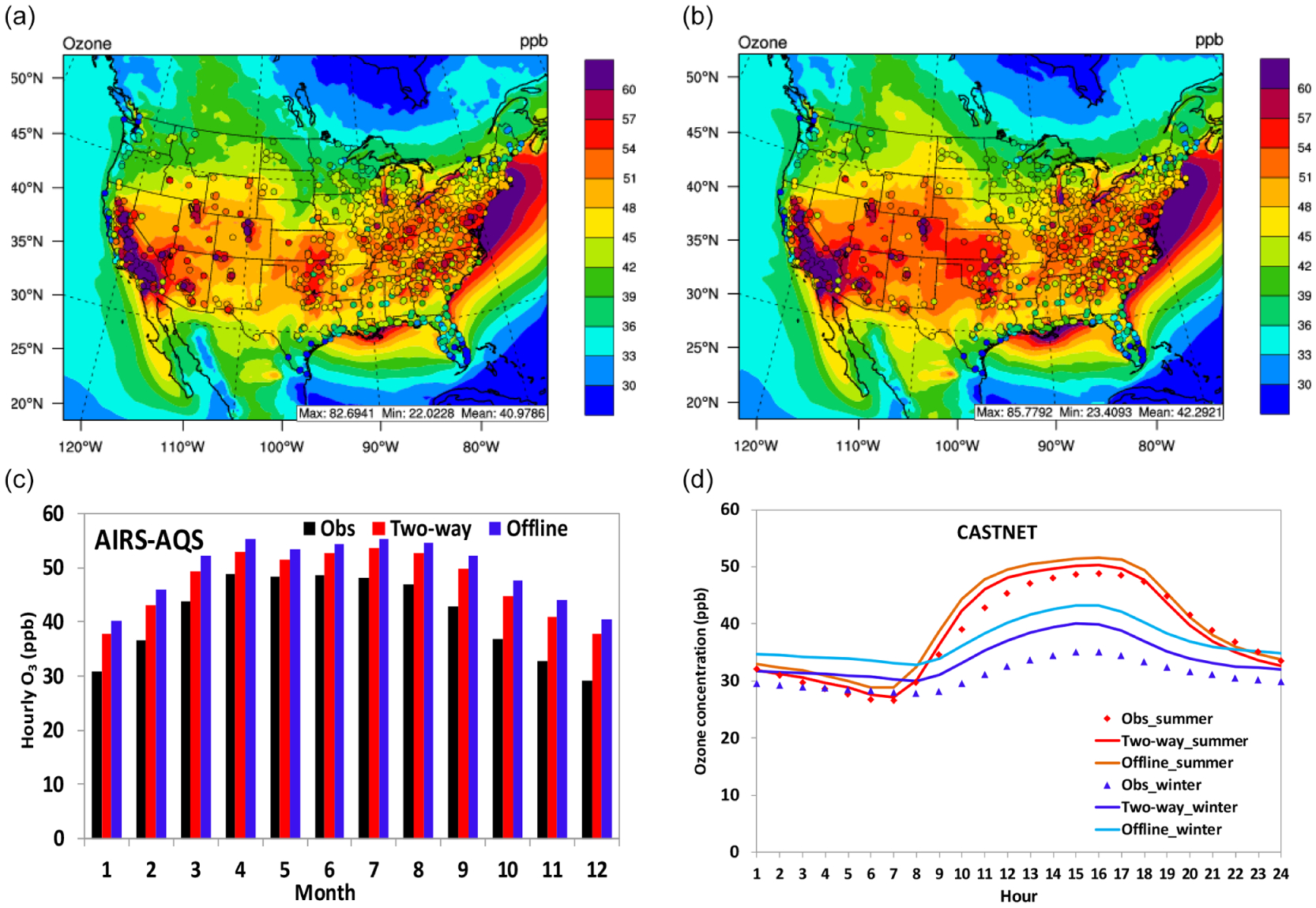
Spatial distributions of 5-year averaged max 8 h O_3_ in summer overlaid with observations from AIRS-AQS and CASTNET for (**a**) two-way WRF-CMAQ and (**b**) offline CMAQ. (**c**) Bar chart for 5-year average monthly O_3_ between observations (black bar), two-way WRF-CMAQ (red bar), and offline CMAQ (blue bar). (**d**) Diurnal plots of observed (dots) vs. simulated (lines) hourly O_3_ concentrations against CASTNET for winter (cold colors) and summer (warm colors) in 2008–2012.

Figure 9a shows the spatial distribution of simulated average daily maximum 8 h O_3_ in summer (2008–2012) from two-way WRF-CMAQ overlaid with observations from both the AIRS-AQS and CASTNET networks. WRF-CMAQ shows good performance by capturing the spatial distribution of max 8 h O_3_ over widespread areas of the domain. The model tends to overpredict O_3_ along coastlines in the southeastern US, the Gulf of Mexico, and US Pacific coast, which can be attributed to a poor representation of coastal boundary layers (Yu et al., 2007) and lack of O_3_ sink via halogen chemistry (Sarwar et al., 2015) and deposition to water (Gantt et al., 2017). The simulation also underpredicts O_3_ in widespread areas in the Midwest, central US, and mountainous regions of the US, which is consistent with the results of 36 km simulations from Wang and Zhang (2012) that used an earlier version of CMAQ v4.6 with the same CB05 gas-phase mechanism. In addition to cold T2 biases over those areas (Fig. 1), the underpredictions are also believed to be associated with inaccurate representations of precursor emissions and elevated/complex terrain due to the coarse grid spacing of 36 km over those regions. Wang and Zhang (2012) found that their 12 km simulation showed improved performance over similar regions especially in summer.

Figure 9c shows the monthly variation of domain-average 5-year average O_3_ mixing ratios between observations from AIRS-AQS and simulations from two-way WRF-CMAQ, and Figure 9d shows the diurnal variation of domain-average 5-year average hourly O_3_ mixing ratios between observations from CASTNET and simulations from two-way WRF-CMAQ for winter and summer. As shown in Fig. 9c, the O_3_ mixing ratios are overpredicted throughout the year, which is consistent with overprediction of T2 (figure not shown). The largest overprediction occurs in the relatively cold months such as September to December. It is interesting that the observations show the largest monthly O_3_ mixing ratios in spring and early summer while the simulation shows the peak during the summer. The difference in timing of peak O_3_ between observations and simulations during the year might be associated with uncertainties in the BCONs of O_3_ that reflect impacts of the long-range transport and associated stratosphere–troposphere exchange of O_3_. As shown in Fig. 9d, WRF-CMAQ tends to overpredict O_3_ during most hours (i.e., 02:00–18:00 LT) in summer and throughout the whole day in winter partially due to the overprediction of T2, especially in winter (Fig. 1). The diurnal pattern of O_3_ is captured much better during summer with much less prediction bias, especially during the nighttime, indicating that the model does a better job in predicting the evolution of nocturnal boundary layer and atmospheric chemistry in the warm season than the cold season. The overall overpredictions in this work are also consistent with previous studies (Eder and Yu, 2006; Appel et al., 2007; Wang et al., 2012), although our results show much better nighttime performance owing to the application of the ACM2 scheme that treats both local and non-local closure (Pleim, 2007). As also shown in Table 4, the domain-average NMBs and NMEs for max 8 h O_3_ in summer are 10.6% and 13.2% against AIRS-AQS and −3.0% and 11.5% against CASTNET, respectively. The statistics are also consistent with previous studies using the CMAQ model (Zhang et al., 2009a; Appel et al., 2013, 2017; Penrod et al., 2014) and can be considered to have good performance according to the criteria suggested by Zhang et al. (2013) and Emery et al. (2017).

Figure 3 also shows the bar charts of annual trends for max 8 h O_3_ from two-way WRF-CMAQ against AQS and CASTNET observations in 2008–2012. Two-way WRF-CMAQ systematically overpredicts O_3_, especially against AQS data, with MBs typically > 4.0 ppb. The potential reasons for model biases have been discussed earlier in this section. There are no obvious decreasing or increasing trends for max 8 h O_3_ from AQS or CASTNET observations. However, the model can generally capture the high O_3_ mixing ratios in 2008 and 2010 and the low O_3_ mixing rations in 2009 from both AQS and CASTNET. The similar down and up trends between 2008 to 2010 for O_3_ (i.e., decreasing from 2008 to 2009 and increasing from 2009 to 2010) from AQS observations were also found by Yahya et al. (2016), but not captured by their simulations. Zhang and Wang (2016) was able to reproduce the similar trend over the southeastern US between 2008 and 2010 using their models and attributed the abnormal high 2010 O_3_ mixing ratios to the extreme dry and warm weather conditions during fall 2010.

#### 3.2.2 Aerosols

**Figure 10.**
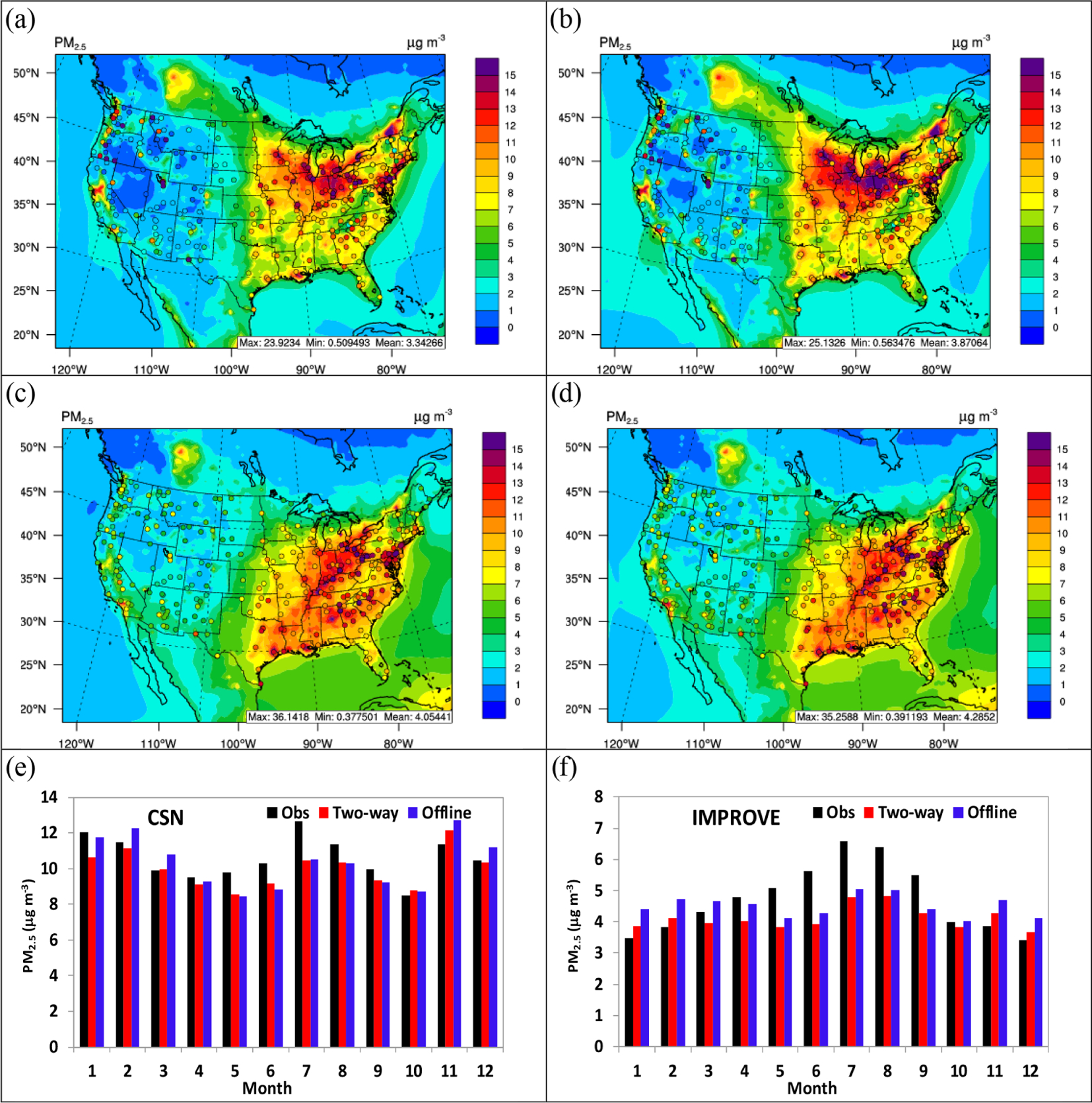
Spatial distributions of 5-year averaged daily PM_2.5_ overlaid with observations from CSN and IMPROVE for two-way WRF-CMAQ in (**a**) winter and (**c**) summer and offline CMAQ in (**b**) winter and (**d**) summer. Bar charts for 5-year average monthly PM_2.5_ between observations (black bar), two-way WRF-CMAQ (red bar), and offline CMAQ (blue bar) over (**e**) CSN and (**f**) IMPROVE in 2008–2012.

Figure 10a and c shows the spatial distribution of simulated 5-year average PM_2.5_ from two-way WRF-CMAQ overlaid with observations from both the CSN and IMPROVE networks in winter and summer, 2008–2012. As shown, WRF-CMAQ performs well for PM_2.5_ over widespread areas of the Midwest and northeastern US in both seasons, while PM_2.5_ is underpredicted over the southeastern and western US, especially in winter. The model also misses some hot spots of observed concentrations in the western US, which are mainly caused by TC underpredictions (Fig. S6) that are likely linked to poorly allocated and underestimated wildfire emissions in the NEI (Wiedinmyer et al., 2006; Roy et al., 2007; Kelly et al., 2019). The relatively large underpredictions over the eastern US are mainly caused by the combined effects from ${\text{SO}}_{4}^{2-}$, ${\text{NH}}_{4}^{+}$, and TC. As shown in Fig. S6, WRF-CMAQ largely underpredicts ${\text{SO}}_{4}^{2-}$ in the Midwest and southeastern US mainly due to the underprediction of oxidants such as O_3_ (see Fig. 9a) (which leads to less production from the gaseous oxidation), overprediction of precipitation (see Fig. 2) (which leads to more wet deposition and removal), and large underprediction of cloud fields (see Figs. 6–7) (which leads to less aqueous-phase formation), over the same area. On the other hand, ${\text{NH}}_{4}^{+}$ and ${\text{NO}}_{3}^{-}$ are either underpredicted or overpredicted, respectively, over the similar areas mainly due to underprediction of ${\text{SO}}_{4}^{2-}$. According to the aerosol thermodynamics, when ${\text{SO}}_{4}^{2-}$ is underpredicted, ${\text{NH}}_{4}^{+}$ tends to be underpredicted due to its major role as cation. More gaseous NH_3_ will be available to neutralize ${\text{NO}}_{3}^{-}$, thus leading to overprediction of ${\text{NO}}_{3}^{-}$ especially over the sulfate-poor regions (West et al., 1999). Other potential reasons include the inaccurate assumptions in the thermodynamic module (for example, the internally mixed aerosol state and equilibrium assumption may not be representative over some regions and different time periods; S. Yu et al., 2006), uncertainties in emissions of key species such as NH_3_ and non-volatile cations that affect particle acidity (Mebust et al., 2003; Wang and Zhang, 2014; Vasilakos et al., 2018; Pye et al., 2020), and measurement errors, especially for ${\text{NO}}_{3}^{-}$ and ${\text{NH}}_{4}^{+}$ (X.-Y. Yu et al., 2006; Karydis et al., 2007; Wang and Zhang, 2012). TC underpredictions over most sites of the domain can be attributed to the underprediction of emissions (e.g., wildfire and primary OC) and underestimation of secondary organic aerosol (SOA) formation (Appel et al., 2017; Pye et al., 2017) since EC (a chemically inert species) is overpredicted, which suggests that atmospheric mixing did not drive the TC underpredictions.

Figure 10e and f show the monthly variation of 5-year average PM_2.5_ between observations from CSN and IMPROVE, respectively, and simulations from two-way WRF-CMAQ. Both observations and WRF-CMAQ show higher PM_2.5_ concentrations at CSN than IMPROVE for the whole year because most of CSN sites are in more polluted urban areas, while the majority of IMPROVE sites are in rural areas and national parks. The model tends to underpredict PM_2.5_ over both CSN and IMPROVE sites in the warm months (i.e., April to September) mainly due to the underpredictions of ${\text{SO}}_{4}^{2-}$ and OC, while it overpredicts PM_2.5_ in cold months mainly due to ${\text{NO}}_{3}^{-}$. The model also captures the seasonality of PM_2.5_ better over CSN sites than IMPROVE sites, especially in the summer months. The large underpredictions over IMPROVE sites during summer months are likely due to the underestimation of precursor emissions (such as wildfire emissions).

**Figure 11.**
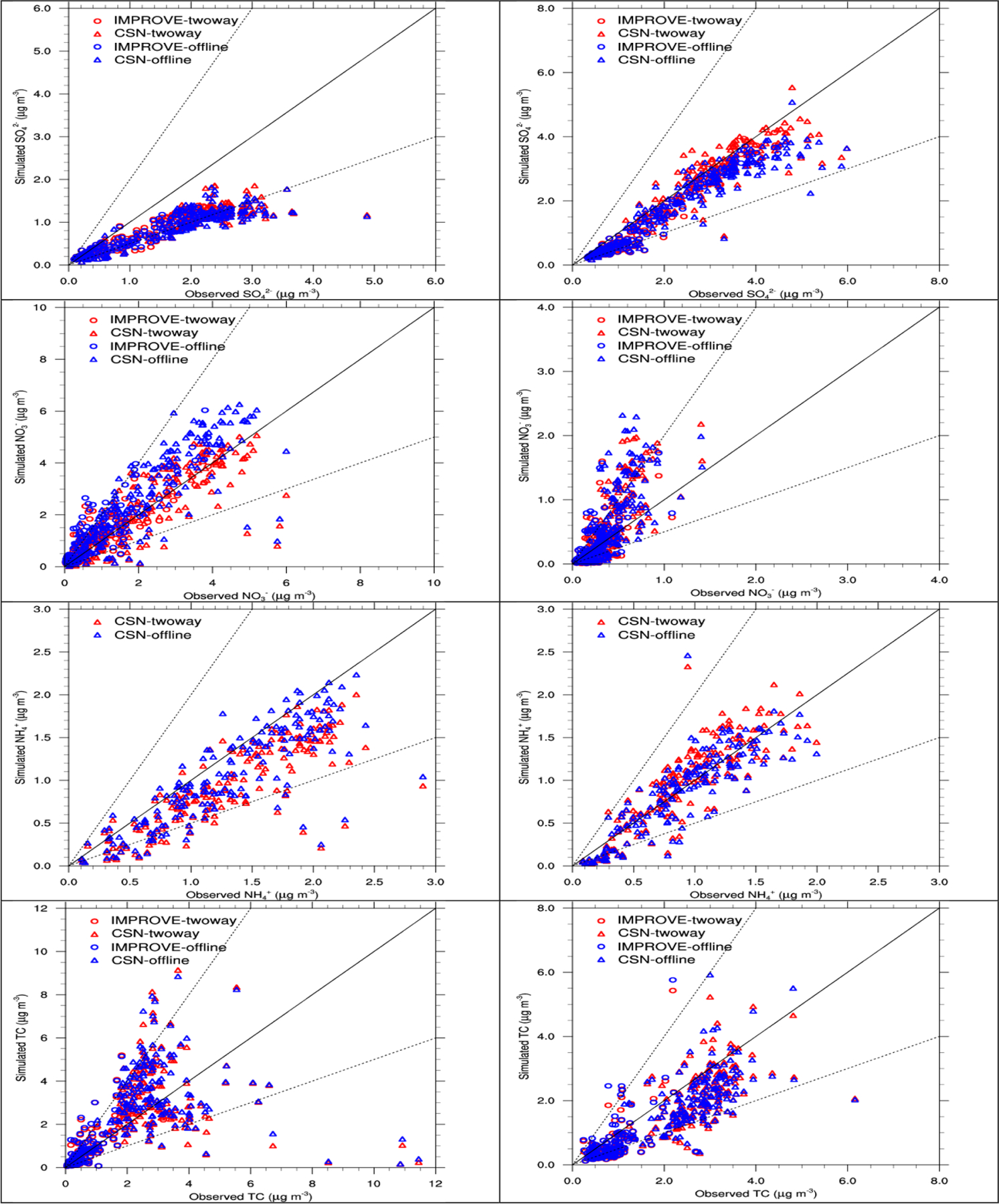
Scatterplots of 5-year averaged PM_2.5_ constituents for ${\text{SO}}_{4}^{2-}$, ${\text{NO}}_{3}^{-}$, ${\text{NH}}_{4}^{+}$, and TC (from top to bottom) between observations and simulations of two-way WRF-CMAQ (red color) and offline CMAQ (blue) in winter (left column) and summer (right column) 2008–2012.

Figure 11 shows the scatterplots of major PM_2.5_ components such as ${\text{SO}}_{4}^{2-}$, ${\text{NH}}_{4}^{+}$, and ${\text{NO}}_{3}^{-}$, and TC in winter and summer 2008–2012. The WRF-CMAQ predicts PM_2.5_ constituents well with majority of data within the 1 : 2 ratio lines in both seasons. Systematic underpredictions of ${\text{SO}}_{4}^{2-}$ and ${\text{NH}}_{4}^{+}$ in winter and overpredictions of ${\text{NO}}_{3}^{-}$ in summer are shown, which are consistent with their spatial distributions. Relatively large underpredictions and overpredictions of TC especially in winter compensate each other and lead to relatively low overall model biases. As also shown in Fig. S6, the model fails to reproduce high concentrations of PM_10_ (those > 20 μg m^−3^) over widespread areas of the domain, especially over dust source areas in California, Arizona, and New Mexico. Hong et al. (2017) found the similar large underprediction of dust using CMAQ v5.0.2 over China and attributed it to a too-high threshold for friction velocity in the current dust module (Dong et al., 2016). Sea salt also seems to be underpredicted by WRF-CMAQ, although sea salt predictions are better than dust as shown along the coastlines.

Figure 3 shows the bar charts of annual averaged observations and simulations for PM_2.5_ over the CSN and IMPROVE sites. Overall, the model performs well for PM_2.5_ for most of years and better over CSN than IMPROVE sites with general underpredictions in most years. The observations for both CSN and IMPROVE show a general decreasing trend, except for 2009 over CSN with a strong drop of PM_2.5_ concentrations. According to EPA (2012), the strong drop of PM_2.5_ in 2009 is due to a few reasons, including the many national and local regulations that are imposed, the contribution of economic slowdown to cleaner air conditions, and favorable meteorological conditions to lower air pollution levels in 2009. The impacts are more apparent over CSN sites mainly composed of urban and suburban areas than IMPROVE sites mainly composed of remote areas and national parks. Two-way WRF-CMAQ is able to reproduce the declining trend well particularly over IMPROVE sites and again demonstrate its capability in accurately simulating the year-to-year variations of not only meteorology but air quality.

As recommended by some previous studies (Zhang et al., 2006; Wang and Zhang, 2012; Emery et al., 2017), generally ±15% and ±30% for model biases and 30% and 50% for model errors can be considered as good and acceptable performance. As shown in Tables 3 and 4, WRF-CMAQ in this work demonstrates an overall good or acceptable performance in predicting aerosols in terms of statistics, especially for PM_2.5_ in both seasons, ${\text{NO}}_{3}^{-}$ OC, and TC in winter, and ${\text{SO}}_{4}^{2-}$ and ${\text{NH}}_{4}^{+}$ in summer. It shows the domain-average NMBs of −7.2% and 8.6% in winter and −13.2% and −26.9% in summer for PM_2.5_ against CSN and IMPROVE, respectively; NMBs of −10.2% and −20.9% in summer for ${\text{SO}}_{4}^{2-}$ against CSN and IMPROVE, respectively; NMBs of −0.3% and 13.3% in winter for ${\text{NO}}_{3}^{-}$ against CSN and IMPROVE, respectively; an NMB of 3.3% for ${\text{NH}}_{4}^{+}$ in summer against CSN; an NMB of 13.0% in winter for OC against IMPROVE; and NMBs of 7.2% and 17.5% in winter for TC against CSN and IMPROVE, respectively. The relatively large underpredictions of PM_10_ in both seasons, i.e., NMBs of −36.3% in winter and −45.8% in summer against AQS, indicate further improvements of dust emissions are warranted. Overall, the aerosol performance is also comparable or better than previous CMAQ or WRF-CMAQ applications (Wang and Zhang, 2012; Penrod et al., 2014; Yu et al., 2014). For example, Penrod et al. (2014) showed 5-year (2001–2005) average NMBs of −23.3% and 4.0% in winter and −19.1% to −17.6% in summer for PM_2.5_ against CSN and IMPROVE data over the CONUS using the CMAQ v5.0, and Yu et al. (2014) reported the monthly mean NMBs of −6.2% and −16.8% for PM_2.5_ against CSN and IMPROVE over the eastern US using the same version of WRF-CMAQ as that used in this study.

#### 3.2.3 Column abundance

**Figure 12.**
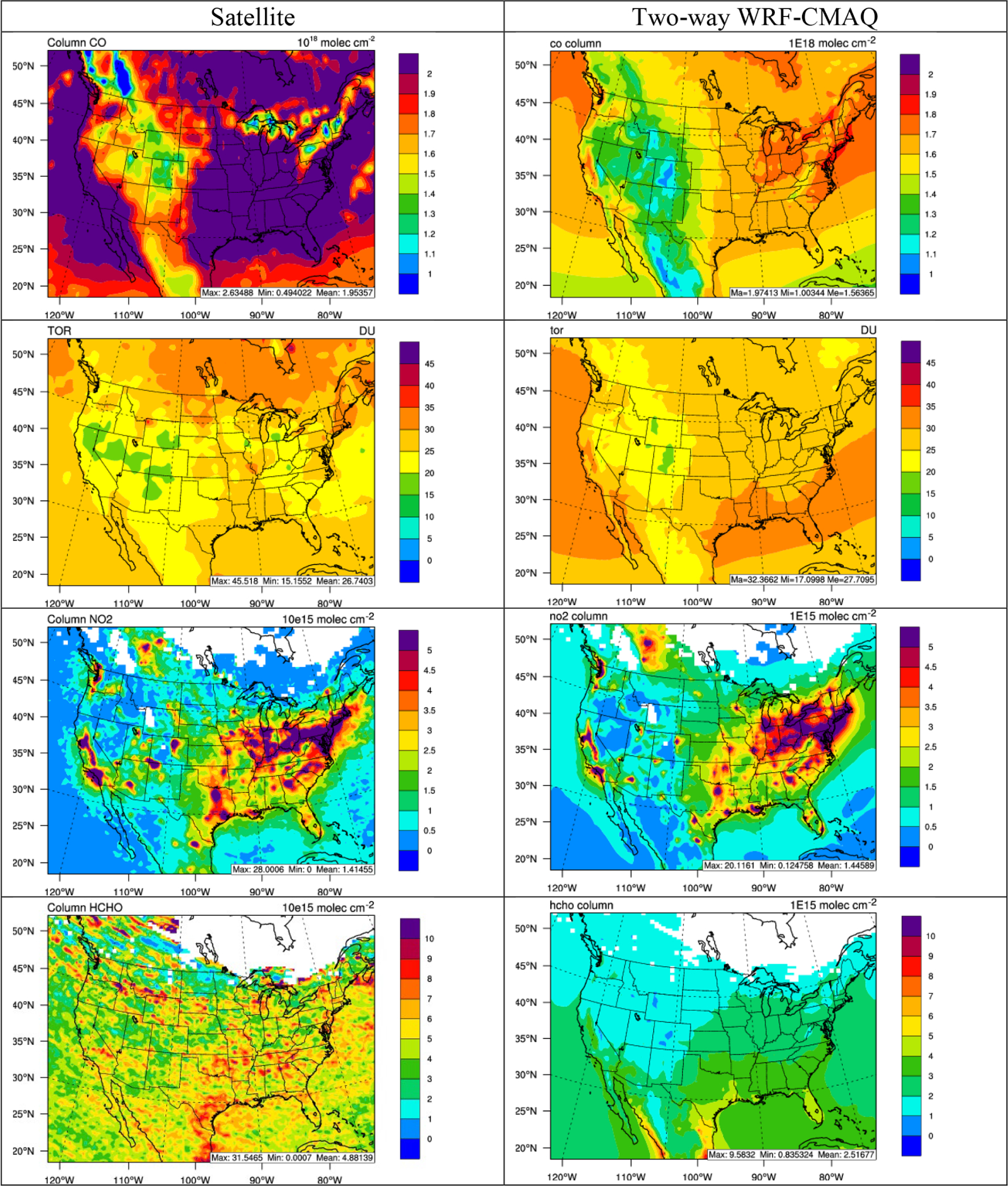
Spatial distribution of 5-year average column abundances (from top to bottom: column CO, TOR, column NO_2_, and column HCHO) between various satellite observations (left column) vs. two-way WRF-CMAQ (right column) in winter 2008–2012.

**Figure 13.**
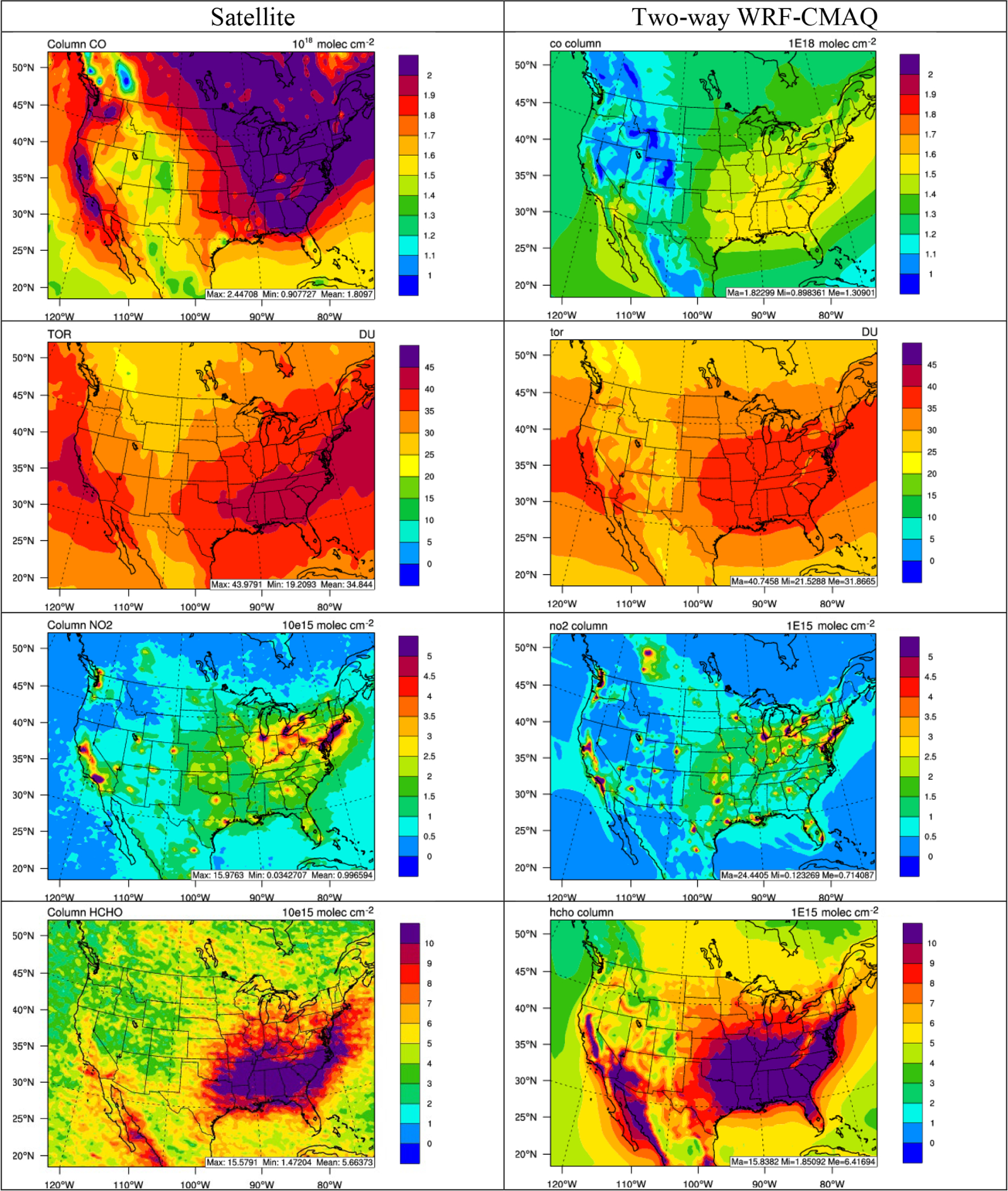
Spatial distribution of 5-year average column abundances (from top to bottom: column CO, TOR, column NO_2_, and column HCHO) between various satellite observations (left column) vs. two-way WRF-CMAQ (right column) in summer 2008–2012.

Figures 12 and 13 show the spatial distribution of 5-year average column abundances between various satellite products and two-way WRF-CMAQ for column CO, TOR, column NO_2_, and column HCHO in winter and summer 2012, and Tables 3 and 4 summarize the statistics. As shown, WRF-CMAQ can reproduce the spatial distribution of the column abundances of gases quite well in both seasons except for column HCHO in winter with *R*s ranging from 0.70 to 0.87. TOR in both seasons, column NO_2_ in winter and column HCHO in summer are also generally well predicted in terms of magnitudes with NMBs of 4.7% for TOR and 0.3% for NO_2_, respectively, in winter and −8.0% for TOR and 15.0% for HCHO, respectively, in summer. Systematic underpredictions for column CO occur in both seasons over the whole domain with NMBs of −20.5% in winter and −27.8% in summer for a few reasons. First, the BCONs of CO may be significantly underestimated from the CESM model. Using WRF/Chem or its variant, Zhang et al. (2016b, 2019) found that the column CO performance could be greatly improved by adjusting the BCON using the satellite observation. A similar approach could be applied in future WRF-CMAQ simulations as well. Second, as pointed by Heald et al. (2003), the regional emissions, especially biomass burning, could be a significant source for elevated CO concentrations, and thus underestimation of these emissions could contribute to the CO underprediction. A more robust set of fire emissions from FINN generated by NCAR based on satellite retrievals has been applied to the similar time period recently but using the WRF-Chem model (Zhang and Wang, 2019) and were found to improve the column CO performance. Finally, Emmons et al. (2009) showed positive biases (i.e., 19%) of MOPITT retrievals over the land when compared to in situ measurements, and the biases may have been increasing over time due to the MOPITT bias drift (e.g., 0.5% yr^−1^ for version 7 retrieval). The predicted TOR can capture the observed high values over the eastern US and oceans and the low values in elevated terrain, especially in summer, and it shows the best performance among all gas species. Both satellite observations and simulations can capture the elevated column NO_2_ over the industrial and metropolitan areas in the domain where large nitrogen oxide (NO_*x*_) emission sources are located, especially in winter. The model shows moderate underprediction with an NMB of −27.8% in summer, which can be attributed to both uncertainties in the emissions and satellite retrievals. For example, the lightning emissions of NO_*x*_ are missing from this study, which have been found by previous studies (Allen et al., 2012) to contribute up to 2.0 × 10^15^ molec. cm^−2^ over the southern US, the Gulf of Mexico, and northern Atlantic Ocean during the summer. Boersma et al. (2004) also found that different column NO_2_ retrieval approaches may lead to large errors (> 25%) over polluted areas. Column HCHO over the CONUS, especially the southeastern US, is well predicted in summer in terms of both magnitude and spatial distribution and correlates well with the biogenic emission source regions. The underprediction of column HCHO in winter may indicate potential underestimation of anthropogenic emissions. Other reasons including potential low yield of HCHO from isoprene and terpene in the CB05 mechanism and uncertainties in satellite retrievals (Stavrakou et al., 2009; Lorente et al., 2017). For example, According to Stavrakou et al. (2009), the air mass factors used for HCHO column calculation may bear ~ 18% error under clear-sky conditions to ~ 50% error for very cloudy conditions. The winter typically has higher cloud cover than summer (see Figs. 6 and 7) and thus higher uncertainties for HCHO column.

#### 3.2.4 Simulated O_3_ and PM_2.5_ exceedances of NAAQS levels

**Figure 14.**
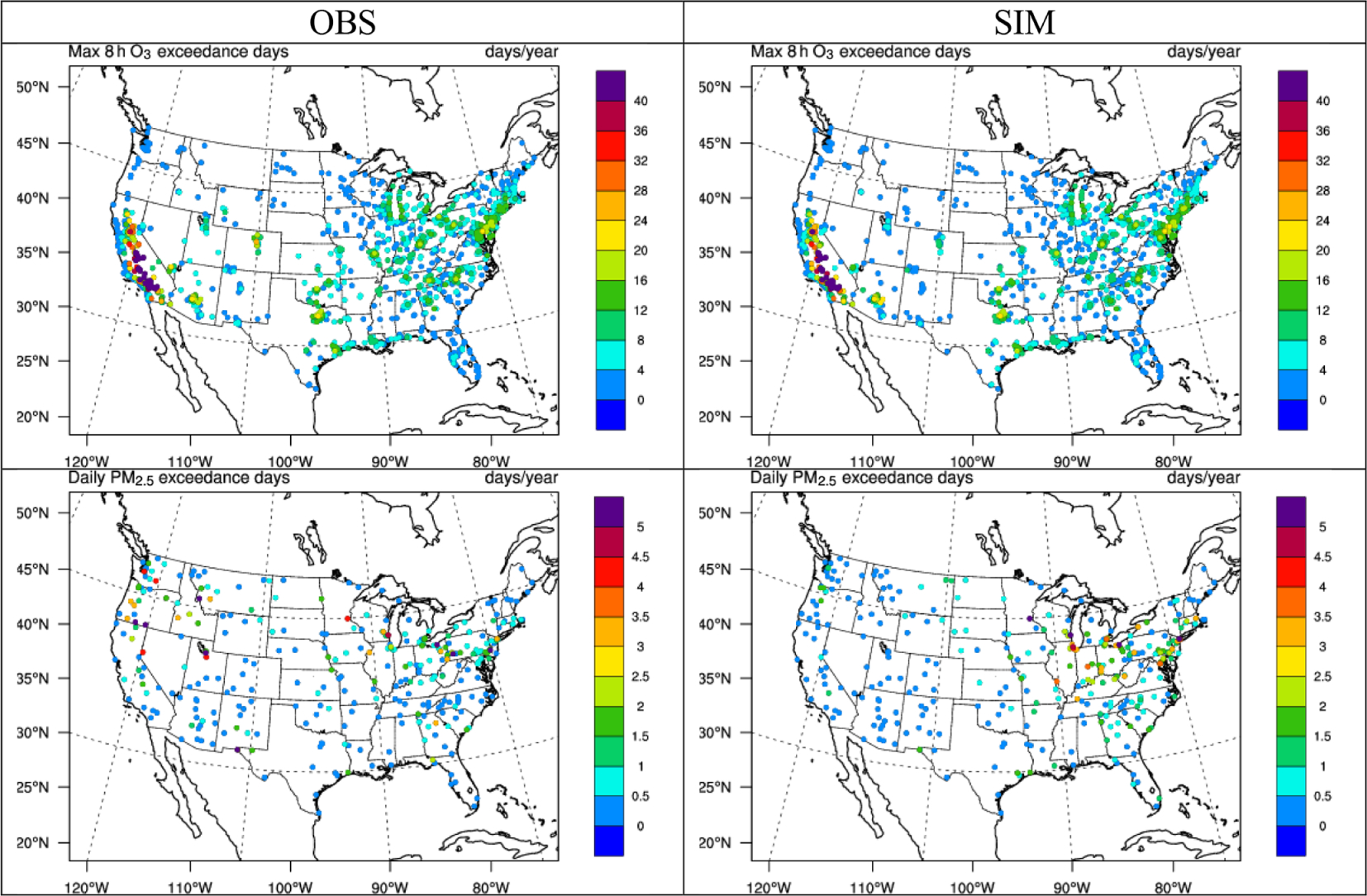
The spatial distribution of 5-year average annual exceedance days of max 8 h O_3_ and daily PM_2.5_ between observations (O_3_ over the AIRS-AQS/CASTNET network and PM_2.5_ over the IMPROVE/CSN network) and two-way WRF-CMAQ in 2008–2012.

National Ambient Air Quality Standards (NAAQS) are set for criteria pollutants, including O_3_ and PM_2.5_, to provide protection against adverse health and welfare effects (https://www.epa.gov/criteria-air-pollutants/naaqs-table, last access: 3 November 2021). In this section, the average number of days per year where the 24 h PM_2.5_ NAAQS level (35 μg m^−3^) and the max 8 h O_3_ NAAQS level (70 ppb) are exceeded from the WRF-CMAQ predictions is compared with the number of exceedances in the monitoring data (i.e., O_3_ from AQS and CASTNET and PM_2.5_ from IMPROVE and CSN). This comparison is intended to better characterize the ability of the model to simulate the high-concentration days that could be especially relevant in regulatory assessments. In Fig. 14, the 5-year average of the annual number of exceedance days is shown for WRF-CMAQ and the monitoring data at monitor locations. As shown, the observations indicate a large number of annual exceedance days for max 8 h O_3_ over major cities, especially in California, Texas, the Midwest, and northeastern US. The spatial distribution of the observed number of exceedance days from the AQS and CASTNET networks aligns well with the non-attainment map reported by the Green Book of the U.S. EPA (https://www.epa.gov/green-book, last access: 3 November 2021). The WRF-CMAQ model also captures the distribution of the number of exceedance days very well, especially in California and the northeastern US. The domain-average values of NMB, NME, and *R* are −3.4%, 14.0%, and 0.98, respectively, also indicating a good performance. For PM_2.5_, the largest number of exceedance days based on the IMPROVE and CSN observations mainly occurs in the northwestern US, the Midwest, and major cities in the northeastern US. The number of exceedance days is generally much lower for PM_2.5_ than O_3_. The spatial distribution of the number of exceedance days for observed PM_2.5_ aligns well with non-attainment areas reported by the Green Book from the U.S. EPA in California. However, the number of simulated PM_2.5_ exceedance days underpredicts the observation-based values in the western US, mainly due to large underpredictions of PM_2.5_ concentrations in the same areas as shown in Fig. 10. The simulation better predicts the distribution of the number of exceedance days in the eastern US where terrain is relatively flat and wildfires are less prevalent. The domain-average values of NMB, NME, and *R* are −29.0%, 80.8%, and 0.21%, respectively.

## 4 Impacts of chemistry–meteorology feedbacks

In this section, the impacts of chemistry–meteorology feedbacks including aerosol direct and indirect effects on regional meteorology and air quality over the US are further examined by comparing results from two-way WRF-CMAQ and offline coupled WRF and CMAQ. Model performance from the two sets of simulations is first compared to demonstrate the potential performance improvements of the two-way model, and the impacts on regional meteorology and air quality are further investigated via the spatial difference plots for selected variables and species.

### 4.1 Meteorology

Figures 2 and 8 compare observations and simulations from the two-way WRF-CMAQ and WRF-only models for precipitation and SWCF/LWCF, respectively. Tables 1 and 2 also summarize the model performance statistics for all major meteorological variables for the two simulations. The statistics of some cloud variables from the WRF-only simulation are not available due to missing model outputs. Overall, good performance is evident for both simulations for surface meteorological variables with slightly better performance for most of variables (except for RH2 in both seasons and T2 in summer) for the two-way WRF-CMAQ simulation than the WRF-only simulation. The MBs for the two-way WRF-CMAQ vs. WRF-only simulation are 1.1 vs. 1.2 °C for T2, 2.2% vs. 2.1% for RH2, 0.57 vs. 0.58 m s^−1^ for WS10, 16.7 vs. 16.9° for WD10, and 0.05–0.71 vs. 0.04–0.72 mm d^−1^ for precipitation in winter and −1.1 vs. −0.9 °C for T2, 3.7% vs. 3.2% for RH2, 0.38 vs. 0.42 m s^−1^ for WS10, 49.1 vs. 49.8° for WD10, and 0.13–0.75 vs. 0.19–0.9 mm d^−1^ for precipitation in summer. The spatial distributions for SWCF and LWCF are better captured in both seasons, especially over the eastern US, Atlantic Ocean, and Gulf of Mexico in winter and over the Midwest and Pacific Northwest in summer. Compared to WRF-only, two-way WRF-CMAQ shows noticeably better performance in terms of both MB and RMSE for radiation and cloud forcing, with MBs of 11.3 vs. 19.5 W m^−2^ for SWDOWN, 7.5 vs. 14.1 W m^−2^ for GSW, −0.9 vs. −6.3 W m^−2^ for GLW, 4.0 vs. 4.7 W m^−2^ for OLR, −3.0 vs. −7.4 W m^−2^ for SWCF, and −3.3 vs. −4.1 W m^−2^ for LWCF in winter and with MBs of 43.6 vs. 59.4 W m^−2^ for SWDOWN, 33.6 vs. 47.2 W m^−2^ for GSW, −13.4 vs. −16.8 W m^−2^ for GLW, 2.3 vs. 3.0 W m^−2^ for OLR, −22.8 vs. −31.1 W m^−2^ for SWCF, and −8.6 vs. −9.0 W m^−2^ for LWCF in summer. These results are consistent with those reported by Yahya et al. (2015a, b) that showed similar improvements in meteorological and radiative variables when comparing predictions from WRF-Chem with those from WRF only. Since identical inputs and physics options are used in both simulations, the differences in performance for meteorological variables is due to the consideration of feedback processes among chemistry, aerosol, cloud, and radiation in the two-way coupled WRF-CMAQ simulation.

**Figure 15.**
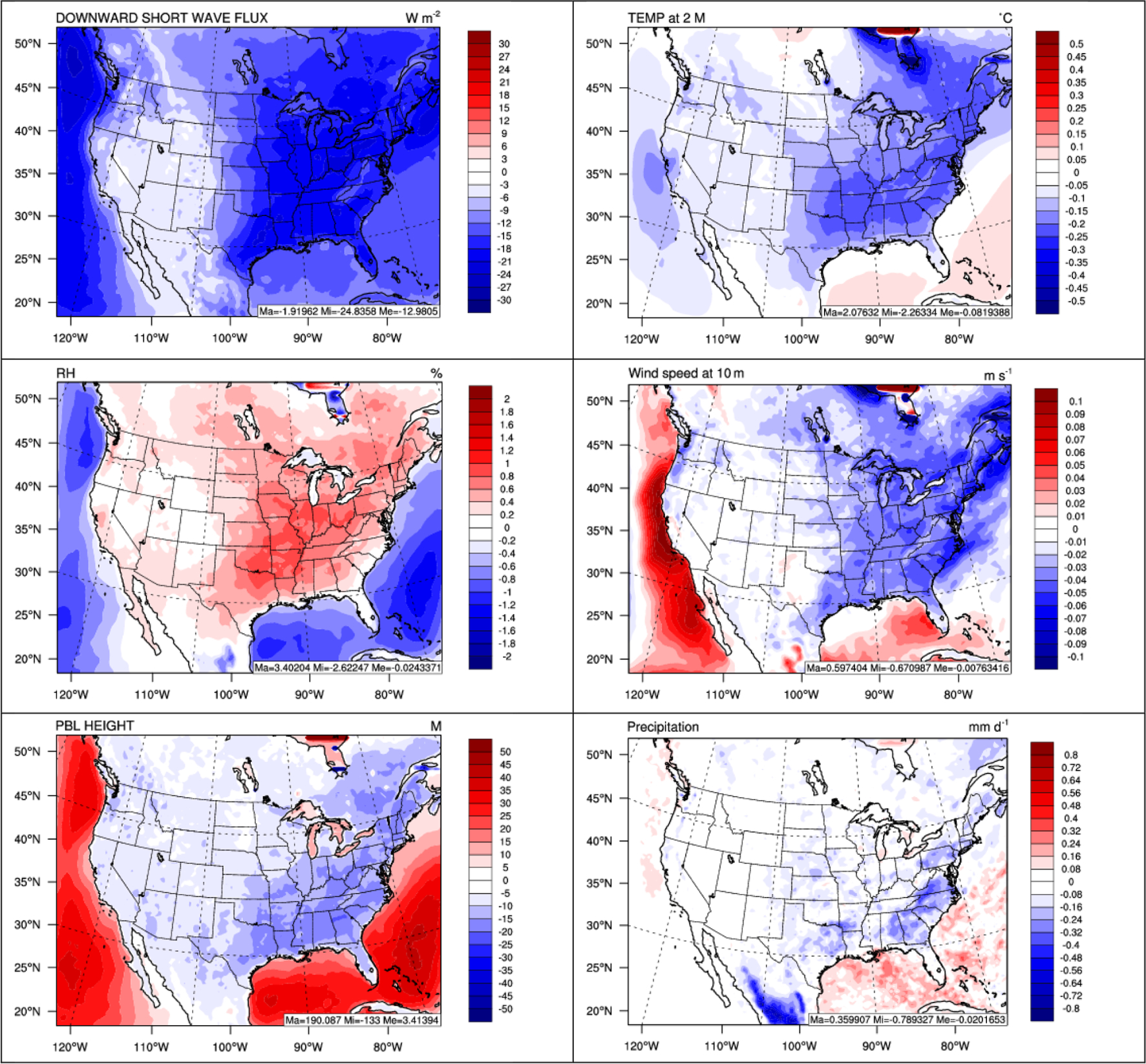
Spatial difference plots (two-way WRF-CMAQ–WRF-only) for major meteorological variables between two-way WRF-CMAQ and WRF-only in 2008–2012.

Figure 15 shows the 5-year average difference plots of selected major meteorological variables including SWDOWN, T2, RH2, WS10, PBL height, and precipitation between two-way WRF-CMAQ and WRF-only in 2008–2012. As shown, the incoming shortwave radiation is reduced by up to 24.8 W m^−2^ (13.6%) with a domain average of 13.0 W m^−2^ (6%) due to the combined aerosol direct and indirect radiative effects over the domain. The reduction is predominant over the eastern US where both aerosol loading and cloud cover are high and over the oceans where cloud cover is high. The magnitude of shortwave radiation reduction in this work is consistent with other studies. For example, Wang et al. (2015a) found that the combined aerosol direct and indirect effects using the WRF/Chem model, which includes the sub-scale cloud forcing not treated in the current WRF-CMAQ model, may decrease the incoming shortwave radiation by 16.0 W m^−2^ in the summer over the US. Hogrefe et al. (2015) reported the reduction of shortwave radiation may reach up to 20 W m^−2^ over the eastern US by only considering the aerosol direct effect using an older version of WRF-CMAQ v5.0.1. Xing et al. (2015b) showed that the aerosol direct forcing may cause the surface shortwave radiation to decrease by up to 10 W m^−2^ over the eastern US over a decadal time period using WRF-CMAQ v5.0. The reduction of shortwave radiation further reduces the surface temperature by up to 0.25 °C over the eastern US, which is much larger than the reduction of 0.1 °C reported by Hogrefe et al. (2015), mainly due to the inclusion of aerosol indirect effects. However, there are smaller reductions in T2 over the Pacific Ocean and even increases (by up to 0.1 °C) over large areas of Atlantic Ocean and Gulf of Mexico where much larger reductions of shortwave radiation occur. As pointed by Wang et al. (2015a), due to the much larger heat capacity of ocean, the response of sea surface temperature is less sensitive to the change of shortwave radiation for ocean compared to the land. The large increase of incoming longwave radiation and latent heat (figures not shown) caused by the aerosol indirect effects and other complex feedback processes over the ocean compensates for the reduction of shortwave radiation, especially over the Atlantic Ocean and Gulf of Mexico, and thus leads to less reduction or even increases of T2. RH2 is found to mostly increase by 3.4% over the land, caused by the decrease of temperature while decrease by 2.6% over the ocean caused by either the increase of temperature or large decrease of water vapor. Over the land, the decreases in shortwave radiation and temperature along with the latent heat (figure not shown) lead to a more stable PBL and thus suppress the wind (by reducing the wind speed as shown). Over the ocean, the changes lead to a more unstable PBL and thus enhance the wind over the ocean. The wind speed and PBL height are reduced by up to 0.05 m s^−1^ and 25 m, respectively, over the US. The aerosol feedbacks on precipitation are also mixed with relatively large decreases by up to 0.4 mm d^−1^ over the US and increases by up to 0.4 mm d^−1^ over oceans. The suppression of precipitation over the land is mainly due to the formation of more small-sized CCNs caused by aerosol indirect effects and align well with areas with high aerosol loadings while the enhancement of precipitation, especially along coastlines and over oceans, might be associated with the larger CCN formation via more activated sea salt particles, as indicated by Zhang et al. (2010) and Wang et al. (2015a).

### 4.2 Air quality

Figures 9–11 compare observations and simulations from two-way WRF-CMAQ and offline CMAQ for O_3_, PM_2.5_, and PM_2.5_ constituents. Tables 3 and 4 summarize the statistics for all major chemical variables for the two simulations. As shown in Fig. 9, two-way WRF-CMAQ shows better performance for both the monthly variation of O_3_ (throughout the whole year) over AQS sites and the diurnal pattern of O_3_ (especially during winter) over CASTNET sites due to better performance of T2 and radiation compared to offline WRF and CMAQ. As shown in Fig. 10, two-way WRF-CMAQ shows better spatial distribution of PM_2.5_ in winter and similar one in summer and better performance for PM_2.5_ for most of months over CSN sites and for cold seasons across IMPROVE sites compared to offline CMAQ. Figure 11 shows systematically better performance for ${\text{SO}}_{4}^{2-}$, ${\text{NO}}_{3}^{-}$, ${\text{NH}}_{4}^{+}$, and TC with more data within 1 : 2 or closer to 1 : 1 ratio lines of scatterplots in both seasons. Overall, as shown in Tables 3 and 4, both simulations show generally good performance for all major chemical species except for PM_10_. For example, the domain-average NMBs are 10.6% (AQS) and −3.0% (CASTNET) vs. 14.2% (AQS) and 0.2% (CASTNET) for O_3_ in summer, −7.2% (CSN) and 8.6% (IMPROVE) vs. 1.8% (CSN) and 23.7% (IMPROVE) for PM_2.5_ in winter and −13.2% (CSN) and −26.9% (IMPROVE) vs. −14.0% (CSN) and −22.8% (IMPROVE) for PM_2.5_ in summer for two-way WRF-CMAQ and offline coupled CMAQ, respectively. The two-way WRF-CMAQ shows better domain-wide statistics in terms of both correlation and biases for many variables including O_3_, ${\text{SO}}_{4}^{2-}$, ${\text{NO}}_{3}^{-}$, and EC as well as TOR and column NO_2_ in both seasons, apparently due to the treatment of chemistry–meteorology feedbacks. Offline CMAQ performs better for total PM_2.5_, especially in the western US due to higher dust emissions from higher wind speed and higher SOA due to stronger radiation and higher temperatures. However, more robust comparisons are needed in the future with improved dust emissions and the use of FINN wildfire emissions.

**Figure 16.**
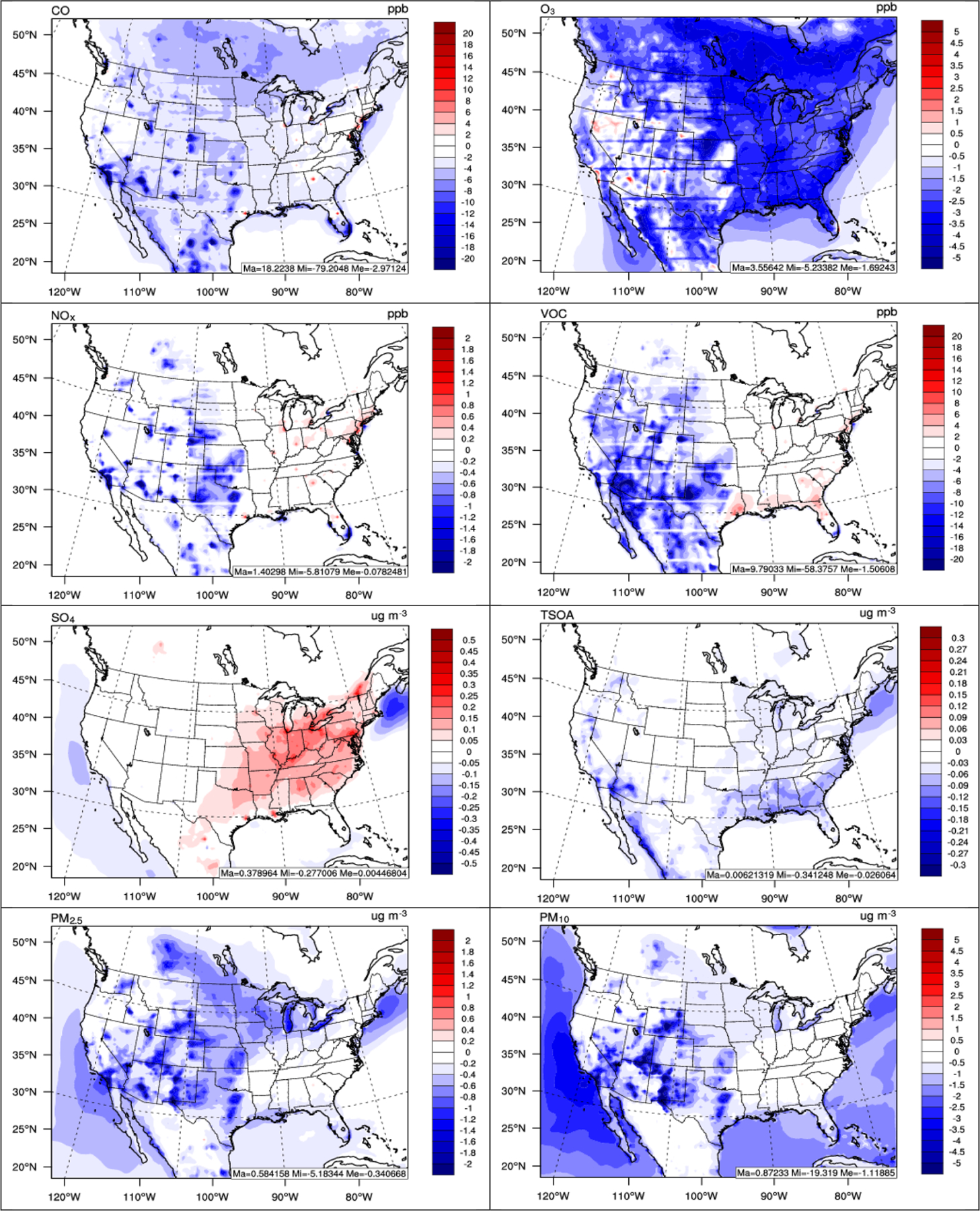
Spatial difference plots (two-way WRF-CMAQ – offline CMAQ) for major chemical species between two-way WRF-CMAQ and offline CMAQ in 2008–2012.

Figure 16 shows the 5-year average difference plots of selected chemical variables including CO, O_3_, NO_*x*_, volatile organic compounds (VOCs), ${\text{SO}}_{4}^{2-}$, SOA, PM_2.5_, and PM_10_ between two-way WRF-CMAQ and offline coupled CMAQ. As shown, the CO mixing ratios decrease by up to 79.2 ppb (27.8%), especially over the western US, with a domain-average reduction of 3.0 ppb (3.1%) due to reduced formation of CO from the oxidation of VOCs caused by reduced solar radiation as indicated by Zhang et al. (2017). Such reductions seem to dominate over the increases caused by reduced PBL height, especially in the western US, where PBL height reductions are at a minimum. The O_3_ mixing ratios decrease by up to 5.2 ppb (16.2%) with domain average of 1.7 ppb (4.2%) mainly due to the reduced solar radiation and T2. The change of O_3_ is consistent with other studies such as Makar et al. (2015) and Wang et al. (2015a) that also reported lower O_3_ mixing ratios caused by aerosol direct and indirect effects. On the other hand, both NO_*x*_ and VOC mixing ratios increase over the eastern US, while they decrease over the western US. The increase should be caused by the combination of the large reduction of PBL mixing and reduced solar radiation, which reduces NO_2_ photolysis and VOC oxidation to SOA. For aerosol species, ${\text{SO}}_{4}^{2-}$ concentrations increase by up to 0.38 μg m^−3^ (26.6%) especially over the eastern US. In fact, the decrease of O_3_ mixing ratios caused by feedbacks is expected to reduce ${\text{SO}}_{4}^{2-}$ production via the gas-phase oxidation pathway due to the influence of O_3_ on OH but increase ${\text{SO}}_{4}^{2-}$ production via the aqueous-phase chemistry pathway due to more clouds in the two-way WRF-CMAQ simulation. Thus, the net increase of ${\text{SO}}_{4}^{2-}$ is more dominated by the aqueous-phase chemistry instead of the gas-phase oxidation. This net increase of ${\text{SO}}_{4}^{2-}$, in turn, leads to an increase of ${\text{NH}}_{4}^{+}$ and decrease of ${\text{NO}}_{3}^{-}$ (figures not shown) through aerosol thermodynamic equilibrium. SOA concentrations decrease by up to 0.34 μg m^−3^ (41.6%), especially over the eastern US, due to the large reduction of oxidants. PM_2.5_ concentrations also decrease by up to 5.2 μg m^−3^ (49.1%) with a domain average of 0.34 μg m^−3^ (8.6%), and PM_10_ concentrations decrease by up to 19.3 μg m^−3^ (64.8%) with a domain average of 1.1 μg m^−3^ (11.1%). The reductions are more apparent over the western US than the eastern US, partially due to the compensation of the increase of ${\text{SO}}_{4}^{2-}$ and ${\text{NH}}_{4}^{+}$ and decrease of other secondary aerosols over the eastern US, as well as the relatively large reduction of dust concentrations over the western US caused by reduced wind speed.

## 5 Summary and conclusion

In this study, two sets of long-term simulations for 2008–2012 using the two-way coupled WRF-CMAQ and offline coupled WRF and CMAQ, respectively, are conducted, evaluated, and compared to investigate the performance improvements due to chemistry–meteorology feedbacks and impacts of those feedbacks on the reginal air quality in the US. First, the two-way coupled WRF-CMAQ simulation with both aerosol direct and indirect radiative forcing is comprehensively evaluated in both winter and summer seasons, and the annual trend is examined between observations and simulations for selected major variables. The results show that WRF-CMAQ performs well for major surface meteorological variables such as temperature at 2 m, relative humidity at 2 m, wind speed at 10 m, and precipitation with domain-average MBs of −1.1 to 1.1 °C, 2.2%–3.7%, 0.38–0.57 m s^−1^, and 0.13–0.23 mm d^−1^ (except for 0.71–0.75 mm d^−1^ against NCDC), respectively, in winter and summer. The relatively large positive biases for precipitation are found to be more apparent when observed precipitation is low (dominated more by the non-convective precipitation) and are thus believed to be more associated with uncertainties in the Morrison microphysics scheme. The long-term simulation also shows generally good performance for major radiation and cloud radiative variables. Relatively large model biases still exist for cloud variables such as CDNC, COT, and CWP, indicating that the processes associated with aerosol indirect effects are still not well understood and an accurate simulation of those effects is still challenging using state-of-the-science models. WRF-CMAQ can also capture the observed year-to-year variations well for almost all the major meteorological and chemical variables.

Two-way WRF-CMAQ also shows generally good or acceptable performance for max 8 h O_3_, PM_2.5_, and PM_2.5_ constituents, with NMBs generally within ±15% for O_3_ and ±30% for PM_2.5_ species. For example, the domain-average NMBs are 10.6% and −3.0% for max 8 h O_3_ against AQS and CASTNET in summer and −13.2% to 7.2% and −26.9% to 8.6% for PM_2.5_ against CSN and IMPROVE in both seasons. O_3_ mixing ratios are overpredicted for most months, especially in the winter, in part due to the larger overprediction of T2 during the cold season. The overall model biases are small for PM_2.5_ due to the compensation of relatively large underpredictions of ${\text{SO}}_{4}^{2-}$ and OC, especially in the warm season, and overprediction of ${\text{NO}}_{3}^{-}$ in the cold season. In addition to biases inherited from the meteorology, the model performance for chemistry also suffers from uncertainties associated with emissions, the use of a coarse spatial resolution, and representation of aerosol formation pathways in the model. For example, the relatively large biases for EC might be associated with poorly allocated anthropogenic or wildfire emissions, and those for OC might be due to underestimation of SOA formation in version 5.0.2 of CMAQ. WRF-CMAQ also predicts the column abundances of chemical species well and the relatively large model biases for CO are found to be associated with an underestimation of BCONs. The model better reproduces the observed number of exceedance days for O_3_ than PM_2.5_ mainly due to better performance for O_3_ than PM_2.5_ concentrations.

The performance comparison between two-way WRF-CMAQ and WRF-only simulations shows that two-way WRF-CMAQ model performs better for major surface meteorological, radiation, and cloud radiative variables due to the consideration of chemistry–meteorology feedbacks associated with aerosol direct and indirect forcing. The feedbacks are found to reduce the 5-year average SWDOWN by up to 24.8 W m^−2^, T2 by up to 0.25 °C, PBL height by up to 25 m, wind speed by up to 0.05 m s^−1^, and precipitation by up to 0.4 mm d^−1^ over the CONUS, which in turn affect the air quality significantly. As a result of feedbacks, two-way WRF-CMAQ outperforms offline CMAQ for O_3_, ${\text{SO}}_{4}^{2-}$, ${\text{NO}}_{3}^{-}$, ${\text{NH}}_{4}^{+}$, and EC as well as TOR and column NO_2_ in terms of both spatiotemporal variations and domain-average statistics due to better meteorology performance for variables such as T2, WS10, radiation, and precipitation. Despite these improvements, the offline CMAQ performs better for total PM_2.5_ in terms of domain-average statistics, which could be partially caused by the compensation of larger underpredictions and overpredictions of PM_2.5_ constituents. More robust comparison for PM_2.5_ should be performed with improved dust and wildfire emissions in future work. Chemistry–meteorology feedbacks are found to play important roles in affecting US air quality by reducing domain-wide 5-year average surface CO by 3.0 ppb (3.1%) and up to 79.2 ppb (27.8%), O_3_ by 1.7 ppb (4.1%) and up to 5.2 ppb (16.2%), PM_2.5_ by 0.34 μg m^−3^ (8.6%) and up to 5.2 μg m^−3^ (49.1%), and PM_10_ by 1.1 μg m^−3^ (11.1%) and up to 19.3 μg m^−3^ (64.8%), mainly due to reduction of radiation, temperature, and wind speed.

In summary, the two-way coupled WRF-CMAQ modeling in this study shows generally satisfactory and consistent performance for the long-term prediction of regional meteorology and air quality when compared to other studies in the literature. Possible causes for the meteorological and chemical biases that were identified through this work can provide valuable information for future model development to improve the two-way coupled WRF-CMAQ model and those biases should also be considered when making future climate and air quality projections. Non-negligible model improvements for many major meteorological and chemical variables compared to the traditional application of offline coupled WRF and CMAQ suggest the importance of chemistry–meteorology feedbacks, especially aerosol direct and indirect effects. The feedbacks should be considered along with other factors in developing future model applications to inform policy making.

## Supplementary Material

Supplement1

## Figures and Tables

**Table 1.** The 5-year average performance statistics for meteorological variables between two-way WRF-CMAQ and WRF-only simulations in winter 2008–2012.

Variables	Datasets	Mean obs	Two-way WRF-CMAQ					WRF-only				
			Mean sim	*R*	MB	NMB (%)	RMSE	Mean sim	*R*	MB	NMB (%)	RMSE
T2 (°C)		7.5	8.6	0.97	1.1	14.9	1.6	8.6	0.97	1.2	15.8	1.6
RH2 (%)	NCDC	72.9	75.1	0.79	2.2	3.0	6.3	75.0	0.79	2.1	2.8	6.3
WS10 (m s^−1^)		3.93	4.50	0.4	0.57	14.6	1.17	4.50	0.4	0.58	14.6	1.17
WD10 (degree)		166.4	183.1	0.0	16.7	10.0	44.2	183.3	0.0	16.9	10.2	44.4
Precipitation (mm d^−1^)	NCDC	1.54	2.25	0.46	0.71	46.3	1.94	2.26	0.47	0.72	47.0	1.94
	NADP	2.48	2.68	0.77	0.2	8.0	1.14	2.69	0.77	0.21	8.6	1.14
	GPCP	1.81	2.04	0.80	0.23	12.8	1.03	2.04	0.80	0.23	12.8	1.02
	PRISM	1.91	2.08	0.89	0.17	9.0	0.79	2.09	0.89	0.18	9.4	0.79
	TMPA	2.02	2.07	0.81	0.05	2.4	1.01	2.06	0.81	0.04	2.0	1.02
SWDOWN (W m^−2^)		108.5	119.8	0.99	11.3	10.4	13.7	128.0	0.98	19.5	17.9	22.2
GSW (W m^−2^)	CERES	87.1	94.6	0.99	7.5	8.6	10.1	101.3	0.98	14.1	16.2	17.1
GLW (W m^−2^)		278.9	278.0	0.99	−0.9	−0.3	5.9	272.7	0.99	−6.3	−2.2	8.6
OLR (W m^−2^)		222.3	226.2	0.99	4.0	1.8	5.1	227.0	0.99	4.7	2.1	5.8
SWCF (W m^−2^)		−26.6	−23.6	0.91	−3.0	−11.1	6.3	−19.2	0.85	−7.4	−27.8	10.6
LWCF(W m^−2^)		22.0	18.7	0.76	−3.3	−15.1	6.0	18.0	0.72	−4.1	−18.4	6.7
AOD		0.11	0.04	0.44	−0.06	−59.8	0.08	NA	NA	NA	NA	NA
CF		0.66	0.59	0.87	−0.07	−10.4	0.1	NA	NA	NA	NA	NA
CDNC (cm^−3^)	MODIS	172.3	30.4	0.21	−141.9	−82.4	157.5	NA	NA	NA	NA	NA
CWP (g m^−2^)		177.4	97.0	0.63	−80.4	−45.3	93.2	NA	NA	NA	NA	NA
COT		16.9	3.3	0.74	−13.6	−80.8	14.2	NA	NA	NA	NA	NA

NA: outputs of AOD, CF, CDNC, CWP, and COT are not available from WRF-only simulations.

**Table 2.** The 5-year average performance statistics for meteorological variables between two-way WRF-CMAQ and WRF-only simulations in summer 2008–2012.

Variables	Datasets	Mean obs	Two-way WRF-CMAQ					WRF-only				
			Mean sim	*R*	MB	NMB (%)	RMSE	Mean sim	*R*	MB	NMB (%)	RMSE
T2 (°C)		22.3	22.2	0.95	−1.1	−4.6	1.7	22.4	0.95	−0.9	−3.7	1.6
RH2 (%)	NCDC	67.0	70.7	0.91	3.7	5.5	6.6	70.1	0.91	3.2	4.7	6.3
WS10 (m s^−1^)		3.19	3.57	0.36	0.38	11.8	0.99	3.61	0.35	0.42	13.1	1.01
WD10 (degree)		146.4	195.4	0.0	49.1	33.5	67.3	196.1	0.0	49.8	34.0	67.9
Precipitation (mm d^−1^)	NCDC	2.11	2.86	0.5	0.75	35.6	1.93	3.01	0.5	0.9	42.6	2.01
	NADP	2.82	2.99	0.83	0.17	5.9	0.87	3.14	0.83	0.32	11.2	0.93
	GPCP	2.55	2.78	0.80	0.23	9.0	1.19	2.86	0.80	0.30	11.9	1.21
	PRISM	2.35	2.55	0.89	0.20	8.4	0.69	2.65	0.89	0.30	12.9	0.73
	TMPA	2.70	2.83	0.80	0.13	4.8	1.27	2.89	0.81	0.19	6.8	1.27
SWDOWN (W m^−2^)		254.7	298.3	0.84	43.6	17.1	46.6	314.1	0.73	59.4	23.3	62.8
GSW (W m^−2^)	CERES	222.5	256.1	0.75	33.6	15.1	37.6	269.7	0.57	47.2	21.2	51.7
GLW (W m^−2^)		372.2	358.8	0.98	−13.4	−3.6	15.3	355.4	0.98	−16.8	−4.5	18.7
OLR (W m^−2^)		257.2	259.6	0.96	2.3	0.9	4.8	260.2	0.96	3.0	1.2	5.2
SWCF (W m^−2^)		−55.1	−32.3	0.69	−22.8	−41.3	27.6	−24.0	0.50	−31.1	−56.4	36.2
LWCF (W m^−2^)		26.1	17.5	0.85	−8.6	−33.0	9.8	17.1	0.87	−9.0	−34.6	10.0
AOD	MODIS	0.20	0.07	0.67	−0.13	−67.8	0.14	NA	NA	NA	NA	NA
CF		0.53	0.41	0.81	−0.12	−23.0	0.16	NA	NA	NA	NA	NA
CDNC (cm^−3^)		138.9	28.9	0.11	−110.0	−79.2	124.1	NA	NA	NA	NA	NA
CWP (g m^−2^)		162.2	54.6	0.65	−107.6	−66.3	113.8	NA	NA	NA	NA	NA
COT		14.2	2.3	0.73	−11.9	−83.6	12.2	NA	NA	NA	NA	NA

NA: outputs of AOD, CF, CDNC, CWP, and COT are not available from WRF-only simulations.

**Table 3.** The 5-year average performance statistics for chemical variables between two-way WRF-CMAQ and offline CMAQ simulations in winter 2008–2012.

Variables	Datasets	Mean obs	Two-way WRF-CMAQ					Offline CMAQ				
			Mean sim	*R*	MB	NMB (%)	NME (%)	Mean sim	*R*	MB	NMB (%)	NME (%)
Max 8 h O_3_ (ppb)	AQS	32.4	39.6	0.61	7.2	22.5	23.0	42.3	0.65	9.9	30.7	30.9
	CASTNET	34.9	36.6	0.76	1.7	4.9	9.4	39.7	0.75	4.7	13.5	14.3
PM_2.5_ (μg m^−3^)	CSN	11.4	10.6	0.21	−0.8	−7.2	29.3	11.7	0.2	0.21	1.8	31.0
	IMPROVE	3.59	3.90	0.83	0.31	8.6	30.3	4.44	0.86	0.85	23.7	32.1
PM_10_ (μg m^−3^)	AQS	19.9	12.7	0.04	−7.2	−36.3	46.9	15.7	0.17	−4.2	−21.3	42.8
${\text{SO}}_{4}^{2-}$ (μg m^−3^)	CSN	2.06	1.06	0.78	−1.0	−48.3	48.4	1.02	0.78	−1.04	−50.7	50.8
	IMPROVE	0.79	0.49	0.95	−0.3	−37.4	38.9	0.49	0.95	−0.3	−38.5	39.9
${\text{NO}}_{3}^{-}$ (μg m^−3^)	CSN	2.37	2.36	0.79	−0.01	−0.3	25.8	2.89	0.81	0.52	21.7	37.8
	IMPROVE	0.73	0.83	0.87	0.1	13.3	40.9	1.06	0.90	0.33	44.6	54.4
${\text{NO}}_{4}^{+}$ (μg m^−3^)	CSN	1.30	0.92	0.80	−0.38	−29.4	30.5	1.03	0.81	−0.27	−21.0	24.1
EC (μg m^−3^)	CSN	0.69	0.75	0.18	0.06	8.7	58.5	0.79	0.24	0.1	14.2	58.0
	IMPROVE	0.17	0.23	0.80	0.06	40.8	59.2	0.25	0.84	0.09	53.4	65.6
OC (μg m^−3^)	IMPROVE	0.65	0.74	0.65	0.09	13.0	55.7	0.8	0.67	0.15	23.1	56.4
TC (μg m^−3^)	CSN	3.05	3.27	0.01	0.22	7.2	53.2	3.49	0.0	0.44	14.4	55.8
	IMPROVE	0.53	0.62	0.75	0.09	17.5	51.3	0.68	0.78	0.15	28.1	52.6
Col. CO (10^18^ molec.cm^−3^)	MOPITT	1.96	1.56	0.70	−0.4	−20.5	21.6	1.57	0.69	−0.39	−19.8	21.1
TOR (DU)	OMI	26.4	27.6	0.78	1.2	4.7	14.0	28.0	0.19	1.6	5.9	14.3
Col. NO_2_ (10^15^ molec. cm^−3^)	SCIAMACHY	1.55	1.55	0.86	0.04	0.3	33.5	1.53	0.87	−0.02	−1.2	33.1
Col. HCHO (10^15^ molec. cm^−3^)	SCIAMACHY	4.87	2.48	0.29	−2.39	−49.0	50.1	2.53	0.28	−2.34	−48.0	49.2

**Table 4.** The 5-year average performance statistics for chemical variables between two-way WRF-CMAQ and offline CMAQ simulations in summer 2008–2012.

Variables	Datasets	Mean obs	Two-way WRF-CMAQ					Offline CMAQ				
			Mean sim	*R*	MB	NMB (%)	NME (%)	Mean sim	*R*	MB	NMB (%)	NME (%)
Max 8 h O_3_ (ppb)	AQS	47.9	53.0	0.66	5.1	10.6	13.2	54.8	0.66	6.8	14.2	15.6
	CASTNET	47.2	45.8	0.66	−1.4	−3.0	11.5	47.3	0.68	0.1	0.2	10.5
PM_2.5_ (μg m^−3^)	CSN	11.4	9.9	0.74	−1.5	−13.2	20.5	9.8	0.71	−1.6	−14.0	20.8
	IMPROVE	6.19	4.52	0.88	−1.66	−26.9	31.2	4.78	0.86	−1.41	−22.8	28.9
PM_10_ (μg m^−3^)	AQS	26.7	14.5	0.03	−12.2	−45.8	50.7	16.2	0.07	−10.5	−39.4	48.6
${\text{SO}}_{4}^{2-}$ (μg m^−3^)	CSN	2.86	2.57	0.91	−0.29	−10.2	15.1	2.34	0.91	−0.52	−18.1	19.5
	IMPROVE	1.40	1.11	0.98	−0.29	−20.9	21.3	1.08	0.98	−0.31	−22.5	22.6
${\text{NO}}_{3}^{-}$ (μg m^−3^)	CSN	0.49	0.71	0.54	0.22	45.2	70.6	0.77	0.59	0.28	57.2	76.8
	IMPROVE	0.20	0.19	0.6	−0.01	−4.7	71.4	0.22	0.63	0.02	10.3	72.2
${\text{NO}}_{4}^{+}$ (μg m^−3^)	CSN	0.91	0.94	0.86	0.03	3.3	22.4	0.88	0.85	−0.03	−3.6	20.1
EC (μg m^−3^)	CSN	0.56	0.79	0.56	0.23	41.0	56.3	0.79	0.55	0.23	41.9	55.5
	IMPROVE	0.20	0.24	0.56	0.04	20.4	58.8	0.26	0.52	0.06	27.9	63.0
OC (μg m^−3^)	IMPROVE	1.37	0.70	0.31	−0.67	−49.2	54.0	0.75	0.28	−0.62	−45.4	52.4
TC (μg m^−3^)	CSN	2.85	2.17	0.54	−0.67	−23.6	29.3	2.19	0.5	−0.65	−22.9	29.7
	IMPROVE	0.88	0.61	0.56	−0.27	−30.5	47.6	0.66	0.53	−0.23	−25.6	47.6
Col. CO (10^18^ molec. cm^−3^)	MOPITT	1.82	1.32	0.75	−0.5	−27.8	27.8	1.32	0.54	−0.5	−27.3	27.3
TOR (DU)	OMI	35.0	32.2	0.87	−2.8	−8.0	9.0	32.4	0.85	−2.6	−7.3	8.6
Col. NO_2_ (10^15^ molec. cm^−3^)	SCIAMACHY	1.08	0.78	0.81	−0.3	−27.8	38.0	0.78	0.80	−0.3	−27.5	38.1
Col. HCHO (10^15^ molec. cm^−3^)	SCIAMACHY	5.81	6.71	0.82	0.9	15.0	22.5	6.82	0.82	1.01	17.4	23.5

## Data Availability

The model inputs and outputs in this study for both two-way coupled WRF-CMAQ and offline coupled WRF and CMAQ are available upon request. The surface network data including AQS, CASTNET, CSN, IMPROVE, and NADP for model evaluations are available from https://www.cmascenter.org/download/data.cfm#obs (last access: 3 November 2021, CMAS, 2021). The NCDC data are available from https://www.ncei.noaa.gov/data/global-hourly/archive/csv (last access: 3 November 2021, NCEI, 2021b). The GPCP data are available from https://www.ncei.noaa.gov/data/global-precipitation-climatology-project-gpcp-monthly (last access: 3 November 2021, NCEI, 2021a). The CERES data are available from https://ceres-tool.larc.nasa.gov/ord-tool/jsp/EBAF41Selection.jsp (last access: 3 November 2021, NASA CERES, 2021). The MODIS data are available from https://doi.org/10.5067/MODIS/MOD08_M3.061 (Platnick et al., 2015). The OMI and SCIAMACHY data are available from https://www.temis.nl/protocols/tropo.php (last access: 3 November 2021, TEMIS, 2021b) and https://www.temis.nl/airpollution/no2.php (last access: 3 November 2021, TEMIS, 2021a). The MOPITT data are available from https://doi.org/10.5067/TERRA/MOPITT/MOP03JM.009 (NASA/LARC/SD/ASDC, 2021).

## References

[R1] Abdul-Razzak H and Ghan SJ: A parameterization of aerosol activation 2. Multiple aerosol types, J. Geophys. Res, 105, 6837–6844, 2000.

[R2] Alapaty K, Herwehe JA, Otte TL, Nolte CG, Bullock OR, Mallard MS, Kain JS, and Dudhia J: Introducing subgrid-scale cloud feedbacks to radiation for regional meteorological and climate modeling, Geophys. Res. Lett, 39, L24809, 10.1029/2012GL054031, 2012.

[R3] Allen DJ, Pickering KE, Pinder RW, Henderson BH, Appel KW, and Prados A: Impact of lightning-NO on eastern United States photochemistry during the summer of 2006 as determined using the CMAQ model, Atmos. Chem. Phys, 12, 1737–1758, 10.5194/acp-12-1737-2012, 2012.

[R4] Appel KW, Gilliland AB, Sarwar G, and Gilliam RC: Evaluation of the Community Multiscale Air Quality (CMAQ) model version 4.5: Sensitivities impacting model performance: Part I, Ozone, Atmos. Environ, 41, 9603–9615, 2007.

[R5] Appel KW, Pouliot GA, Simon H, Sarwar G, Pye HOT, Napelenok SL, Akhtar F, and Roselle SJ: Evaluation of dust and trace metal estimates from the Community Multiscale Air Quality (CMAQ) model version 5.0, Geosci. Model Dev, 6, 883–899, 10.5194/gmd-6-883-2013, 2013.

[R6] Appel KW, Napelenok SL, Foley KM, Pye HOT, Hogrefe C, Luecken DJ, Bash JO, Roselle SJ, Pleim JE, Foroutan H, Hutzell WT, Pouliot GA, Sarwar G, Fahey KM, Gantt B, Gilliam RC, Heath NK, Kang D, Mathur R, Schwede DB, Spero TL, Wong DC, and Young JO: Description and evaluation of the Community Multiscale Air Quality (CMAQ) modeling system version 5.1, Geosci. Model Dev, 10, 1703–1732, 10.5194/gmd-10-1703-2017, 2017. 30147852 PMC6104654

[R7] Baklanov A, Schlünzen K, Suppan P, Baldasano J, Brunner D, Aksoyoglu S, Carmichael G, Douros J, Flemming J, Forkel R, Galmarini S, Gauss M, Grell G, Hirtl M, Joffre S, Jorba O, Kaas E, Kaasik M, Kallos G, Kong X, Korsholm U, Kurganskiy A, Kushta J, Lohmann U, Mahura A, Manders-Groot A, Maurizi A, Moussiopoulos N, Rao ST, Savage N, Seigneur C, Sokhi RS, Solazzo E, Solomos S, Sørensen B, Tsegas G, Vignati E, Vogel B, and Zhang Y: Online coupled regional meteorology chemistry models in Europe: current status and prospects, Atmos. Chem. Phys, 14, 317–398, 10.5194/acp-14-317-2014, 2014.

[R8] Bennartz R: Global assessment of marine boundary layer cloud droplet number concentration from satellite, J. Geophys. Res, 112, D02201, 10.1029/2006JD007547, 2007.

[R9] Boersma KF, Eskes HJ, and Brinksma EJ: Error analysis for tropospheric NO_2_ retrieval from space, J. Geophys. Res, 109, D04311, 10.1029/2003JD003962, 2004.

[R10] Brunner D, Savage N, Jorba O, Eder B, Giordano L, Badia A, Balzarini A, Baro R, Bianconi R, Chemel C, Curci G, Forkel R, Jimenez-Guerrero P, Hirtl M, Hodzic A, Hozak L, Im U, Knote C, Makar P, Manders-Groot A, van Meijgaard E, Neal L, Perez JL, Pirovano G, San Jose R, Schroder W, Sokhi RS, Syrakov D, Torian A, Tuccella P, Werhahn J, Wolke R, Yahya K, Zabkar R, Zhang Y, Hogrefe C, and Galmarini S: Comparative analysis of meteorological performance of coupled chemistry-meteorology models in the context of AQMEII phase 2, Atmos. Environ, 115, 470–498, 10.1016/j.atmosenv.2014.12.032, 2015.

[R11] Byun DW and Schere KL: Review equations, computational algorithms, and other components of the Models-3 Community Multi-Scale Air Quality (CMAQ) modeling system, Appl. Mech. Rev, 59, 51–77, 10.1115/1.2128636, 2006.

[R12] Choi MW, Lee JH, Woo JW, Kim CH, and Lee SH: Comparison of PM_2.5_ chemical components over East Asia simulated by the WRF-Chem and WRF/CMAQ models: On the models’ prediction inconsistency, Atmosphere, 10, 618, 10.3390/atmos10100618, 2019.

[R13] CMAS (Community Modeling and Analysis System): https://www.cmascenter.org/download/data.cfm#obs, last access: 3 November 2021.

[R14] Cohen AE, Cavallo SM, Coniglio MC, and Brooks HE: A review of planetary boundary layer parameterization schemes and their sensitivity in simulating southeastern U.S. cold season severe weather environments, Weather Forecast, 30, 591–612, 10.1175/WAF-D-14-00105.1, 2015.

[R15] Dong X, Fu JS, Huang K, Tong D, and Zhuang G: Model development of dust emission and heterogeneous chemistry within the Community Multiscale Air Quality modeling system and its application over East Asia, Atmos. Chem. Phys, 16, 8157–8180, 10.5194/acp-16-8157-2016, 2016.

[R16] Eder B and Yu S: A performance evaluation of the 2004 release of Models-3 CMAQ, Atmos. Environ, 40, 4811–4824, 2006.

[R17] Emery C, Liu Z, Russell AG, Odman MT, Yarwood G, and Kumar N: Recommendations on statistics and benchmarks to assess photochemical model performance, J. Air Waste Manage. Assoc, 67, 582–598, 10.1080/10962247.2016.1265027, 2017. 27960634

[R18] Emmons LK, Edwards DP, Deeter MN, Gille JC, Campos T, Nédélec P, Novelli P, and Sachse G: Measurements of Pollution In The Troposphere (MOPITT) validation through 2006, Atmos. Chem. Phys, 9, 1795–1803, 10.5194/acp-9-1795-2009, 2009.

[R19] Gan C-M, Pleim J, Mathur R, Hogrefe C, Long CN, Xing J, Wong D, Gilliam R, and Wei C: Assessment of long-term WRF–CMAQ simulations for understanding direct aerosol effects on radiation “brightening” in the United States, Atmos. Chem. Phys, 15, 12193–12209, 10.5194/acp-15-12193-2015, 2015a.

[R20] Gan C-M, Binkowski F, Pleim J, Xing J, Wong D, Mathur R, and Gilliam R: Assessment of the aerosol optics component of the coupled WRF–CMAQ model using CARES field campaign data and a single column model, Atmos. Environ, 115, 670–682, 2015b.

[R21] Gantt B, He J, Zhang X, Zhang Y, and Nenes A: Incorporation of advanced aerosol activation treatments into CESM/CAM5: model evaluation and impacts on aerosol indirect effects, Atmos. Chem. Phys, 14, 7485–7497, 10.5194/acp-14-7485-2014, 2014.

[R22] Gantt B, Sarwar G, Xing J, Simon H, Schwede D, Hutzell WT, Mathur R, and Saiz-Lopez A: The impact of iodide-mediated ozone deposition and halogen chemistry on surface ozone concentrations across the continental United States, Environ. Sci. Technol, 51, 1458–1466, 2017. 28051851 10.1021/acs.est.6b03556 PMC6145082

[R23] Ghan SJ, Laulainen NS, Easter RC, Wagener R, Nemesure S, Chapman EG, Zhang Y, and Leung LR: Evaluation of aerosol direct radiative forcing in MIRAGE, J. Geophys. Res, 106, 5295–5316, 2001.

[R24] Glotfelty T, He J, and Zhang Y: Impact of future climate policy scenarios on air quality and aerosol-cloud interactions using an advanced version of CESM/CAM5: Part I. model evaluation for the current decadal simulations, Atmos. Environ, 152, 222–239, 2017.

[R25] Grell GA and Baklanov A: Integrated modelling for forecasting weather and air quality: A call for fully coupled approaches, Atmos. Environ, 45, 38, 6845–6851, 2011.

[R26] Grell GA, Peckham SE, Schmitz R, McKenn SA, Frost G, Skamarock WC, and Eder B: Fully Coupled “Online” chemistry within the WRF Model, Atmos. Environ, 39, 6957–6975, 2005.

[R27] He J and Zhang Y: Improvement and further development in CESM/CAM5: gas-phase chemistry and inorganic aerosol treatments, Atmos. Chem. Phys, 14, 9171–9200, 10.5194/acp-14-9171-2014, 2014.

[R28] Heald CL, Jacob DJ, Fiore AM, Emmons LK, Gille JC, Deeter MN, Warner J, Edwards DP, Crawford JH, Hamlin AJ, Sachse GW, Browell EV, Avery MA, Vay SA, Westberg DJ, Blake DR, Singh HB, Sandholm ST, Talbot RW, and Fuelberg HE: Asian outflow and trans-Pacific transport of carbon monoxide and ozone pollution: An integrated satellite, aircraft, and model perspective, J. Geophys. Res, 108, 4804, 10.1029/2003JD003507, 2003.

[R29] Herwehe JA, Otte TL, Mathur R, and Rao ST: Diagnostic analysis of ozone concentrations simulated by two regional-scale air quality models, Atmos. Environ, 45, 5957–5969, 2011.

[R30] Hogrefe C, Pouliot G, Wong D, Torian A, Roselle S, Pleim J, and Mathur R: Annual application and evaluation of the online coupled WRF–CMAQ system over North America under AQMEII phase 2, Atmos. Environ, 115, 683–694, 2015.

[R31] Hong C, Zhang Q, Zhang Y, Tang Y, Tong D, and He K: Multi-year downscaling application of two-way coupled WRF v3.4 and CMAQ v5.0.2 over east Asia for regional climate and air quality modeling: model evaluation and aerosol direct effects, Geosci. Model Dev, 10, 2447–2470, 10.5194/gmd-10-2447-2017, 2017.

[R32] Hong C-P, Zhang Q, Zhang Y, Davis SJ, Zhang X, Tong D, Guan D, Liu Z, and He K-B: Weakened aerosol radiative effects may mitigate the climate penalty on Chinese air quality, Nat. Clim. Change, 10, 845–850, 10.1038/s41558-020-0840-y, 2020.

[R33] Iacono MJ, Delamere JS, Mlawer EJ, Shephard MW, Clough SA, and Collins WD: Radiative forcing by long-lived greenhouse gases: Calculations with the AER radiative transfer models, J. Geophys. Res, 113, D13103, 10.1029/2008JD009944, 2008.

[R34] IPCC: Global warming of 1.5 °C, An IPCC Special Report on the impacts of global warming of 1.5 °C above pre-industrial levels and related global greenhouse gas emission pathways, in the context of strengthening the global response to the threat of climate change, sustainable development, and efforts to eradicate poverty, edited by: Masson-Delmotte V, Zhai P, Pörtner HO, Roberts D, Skea J, Shukla PR, Pirani A, Moufouma-Okia W, Péan C, Pidcock R, Connors S, Matthews JBR, Chen Y, Zhou X, Gomis MI, Lonnoy E, Maycock T, Tignor M, and Waterfield T, World Meteorological Organization, Geneva, Switzerland, 32 pp., 2018.

[R35] Jacobson MZ: GATOR-GCMM: A global-through urban-scale air pollution and weather forecast model 1. Model design and treatment of subgrid soil, vegetation, roads, rooftops, water, sea, ice, and snow, J. Geophys. Res, 106, 5385–5401, 2001.

[R36] Jacobson MZ, Lu R, Turco RP, and Toon OB: Development and application of a new air pollution modeling system. Part I: Gas-phase simulations, Atmos. Environ, 30B, 1939–1963, 1996.

[R37] Jung J, Souri AH, Wong DC, Lee S, Jeon W, Kim J, and Choi Y: The impact of the direct effect of aerosols on meteorology and air quality using aerosol optical depth assimilation during the KORUS-AQ campaign, J. Geophys. Res.-Atmos, 124, 8303–8319, 10.1029/2019JD030641, 2019. 31667043 PMC6820163

[R38] Kain JS: The Kain-Fritsch convective parameterization: An update, J. Appl. Meteorol, 43, 170–181, 10.1175/1520-0450(2004)043<0170:TKCPAU>2.0.CO;2, 2004.

[R39] Karydis VA, Tsimpidi AP, and Pandis SN: Evaluation of a three-dimensional chemical transport model (PMCAMx) in the eastern United States for all four seasons, J. Geophys. Res, 112, D14211, 10.1029/2006JD007890, 2007.

[R40] Kaufman YJ, Smirnov A, Holben B, and Dubovik O: Baseline maritime aerosol methodology to derive the optical thickness and scattering properties, Geophys. Res. Lett, 28, 3251, 10.1029/2001GL013312, 2001.

[R41] Kelly J, Koplitz S, Baker K, Holder A, Pye H, Murphy B, Bash J, Henderson B, Possiel N, Simon H, Eyth A, Jang C, Phillips S, and Timin B: Assessing PM_2.5_ model performance for the conterminous U.S. with comparison to model performance statistics from 2007–2015, Atmos. Environ, 214, 116872, 10.1016/j.atmosenv.2019.116872, 2019. PMC6859642 31741655

[R42] Kukkonen J, Olsson T, Schultz DM, Baklanov A, Klein T, Miranda AI, Monteiro A, Hirtl M, Tarvainen V, Boy M, Peuch V-H, Poupkou A, Kioutsioukis I, Finardi S, Sofiev M, Sokhi R, Lehtinen KEJ, Karatzas K, San José R, Astitha M, Kallos G, Schaap M, Reimer E, Jakobs H, and Eben K: A review of operational, regional-scale, chemical weather forecasting models in Europe, Atmos. Chem. Phys, 12, 1–87, 10.5194/acp-12-1-2012, 2012.

[R43] Li P, Wang L, Guo P, Yu S, Mehmood K, Wang S, Liu W, Seinfeld JH, Zhang Y, Wong D, Alapaty K, Pleim J, and Mathur R: High reduction of ozone and particulate matter during the 2016 G-20 summit in Hangzhou by forced emission controls of industry and traffic, Environ. Chem. Lett, 15, 709–715, 10.1007/s10311-017-0642-2, 2017. 29713260 PMC5920520

[R44] Lin M, Holloway T, Carmichael GR, and Fiore AM: Quantifying pollution inflow and outflow over East Asia in spring with regional and global models, Atmos. Chem. Phys, 10, 4221–4239, 10.5194/acp-10-4221-2010, 2010.

[R45] Liu X-H, Zhang Y, Xing J, Zhang Q, Wang K, Streets DG, Jang CJ, Wang W-X, and Hao JM: Understanding of regional air pollution over China using CMAQ: Part II. Process analysis and ozone sensitivity to precursor emissions, Atmos. Environ, 44, 3719–3727, 2010.

[R46] Lorente A, Folkert Boersma K, Yu H, Dörner S, Hilboll A, Richter A, Liu M, Lamsal LN, Barkley M, De Smedt I, Van Roozendael M, Wang Y, Wagner T, Beirle S, Lin J-T, Krotkov N, Stammes P, Wang P, Eskes HJ, and Krol M: Structural uncertainty in air mass factor calculation for NO_2_ and HCHO satellite retrievals, Atmos. Meas. Tech, 10, 759–782, 10.5194/amt-10-759-2017, 2017.

[R47] Ma P-L, Rasch PJ, Fast JD, Easter RC, Gustafson WI Jr., Liu X, Ghan SJ, and Singh B: Assessing the CAM5 physics suite in the WRF-Chem model: implementation, resolution sensitivity, and a first evaluation for a regional case study, Geosci. Model Dev, 7, 755–778, 10.5194/gmd-7-755-2014, 2014.

[R48] Makar PA, Gonga W, Hogrefe C, Zhang Y, Curci G, Žabkar R, Milbrandt J, Im U, Balzarini A, Baró R, Bianconi R, Cheung P, Forkel R, Gravel S, Hirtl M, Honzak L, Hou A, Jiménez-Guerrero P, Langer M, Moran MB, Pabla B, Pérez JL, Pirovano G, San José R, Tuccella P, Werhahn J, Zhang J, and Galmarini S: Feedbacks between air pollution and weather, Part 2: Effects on chemistry, Atmos. Environ, 115, 499–526, 2015.

[R49] Mathur R, Xiu A, Coats C, Alapaty K, Shankar U, and Hanna A: Development of an air quality modeling system with integrated meteorology, chemistry, and emissions, Proc. Measurement of Toxic and Related Air Pollutants, AWMA, Cary, NC, 1–3 September 1998.

[R50] Mathur R, Xing J, Gilliam R, Sarwar G, Hogrefe C, Pleim J, Pouliot G, Roselle S, Spero TL, Wong DC, and Young J: Extending the Community Multiscale Air Quality (CMAQ) modeling system to hemispheric scales: overview of process considerations and initial applications, Atmos. Chem. Phys, 17, 12449–12474, 10.5194/acp-17-12449-2017, 2017. 29681922 PMC5907506

[R51] Matsui H, Koike M, Kondo Y, Takegawa N, Kita K, Miyazaki Y, Hu M, Chang S-Y, Blake DR, Fast JD, Zaveri RA, Streets DG, Zhang Q and Zhu T: Spatial and temporal variations of aerosols around Beijing in summer 2006: Model evaluation and source apportionment, J. Geophys. Res, 114, D00G13, 10.1029/2008JD010906, 2009.

[R52] Mebust MR, Eder BK, Binkowski FS, and Roselle SJ: Models-3 Community Multiscale Air Quality (CMAQ) model aerosol component: 2. Model evaluation, J. Geophys. Res, 108, 4184, 10.1029/2001JD001410, 2003.

[R53] Mehmood K, Wu Y, Wang L, Yu S, Li P, Chen X, Li Z, Zhang Y, Li M, Liu W, Wang Y, Liu Z, Zhu Y, Rosenfeld D, and Seinfeld JH: Relative effects of open biomass burning and open crop straw burning on haze formation over central and eastern China: modeling study driven by constrained emissions, Atmos. Chem. Phys, 20, 2419–2443, 10.5194/acp-20-2419-2020, 2020.

[R54] Morrison H, Thompson G, and Tatarskii V: Impact of cloud microphysics on the development of trailing stratiform precipitation in a simulated squall line: Comparison of one- and two-moment schemes, Mon. Weather Rev, 137, 991–1007, 10.1175/2008MWR2556.1, 2009.

[R55] NASA CERES: CERES_EBAF_Ed4.1 Subsetting and Browsing, NASA [data set], available at: https://ceres-tool.larc.nasa.gov/ord-tool/jsp/EBAF41Selection.jsp, last access: 3 November 2021.

[R56] NASA/LARC/SD/ASDC: MOPITT CO gridded monthly means (Near and Thermal Infrared Radiances) V009, NASA Langley Atmospheric Science Data Center DAAC [data set], 10.5067/TERRA/MOPITT/MOP03JM.009, last access: 3 November 2021.

[R57] NCAR (National Center for Atmospheric Research): WRFv3.4, NCAR [code], available at: https://www2.mmm.ucar.edu/wrf/src/WRFV3.4.TAR.gz (last access: 3 November 2021), 2012.

[R58] NCEI (National Centers for Environmental Information): GPCP data, available at: https://www.ncei.noaa.gov/data/global-precipitation-climatology-project-gpcp-monthly, last access: 3 November 2021a.

[R59] NCEI (National Centers for Environmental Information): NCDC data, available at: https://www.ncei.noaa.gov/data/global-hourly/archive/csv, last access: 3 November 2021b.

[R60] Penrod A, Zhang Y, Wang K, Wu S-Y, and Leung RL: Impacts of future climate and emission changes on US air quality, Atmos. Environ, 89, 533–547, 10.1016/j.atmosenv.2014.01.001, 2014.

[R61] Platnick S, Hubanks P, Meyer K. and King MD: MODIS Atmosphere L3 Monthly Product (08_L3), NASA MODIS Adaptive Processing System, Goddard Space Flight Center [data set], 10.5067/MODIS/MOD08_M3.006 (Terra), 2015.

[R62] Pleim J, Young J, Wong D, Gilliam R, Otte T, and Mathur R: Two-way coupled meteorology and air quality modeling, in Air Pollution Modeling and its Application, edited by: Borrego C and Miranda AI, XIX, NATO Science for Peace and Security Series, Series C: Environmental Security, Springer, Dordrecht, 2008.

[R63] Pleim JE: A combined local and nonlocal closure model for the atmospheric boundary layer. Part I: Model description and testing, J. Appl. Meteorol. Clim, 46, 1383–1395, 10.1175/JAM2539.1, 2007.

[R64] Pleim JE and Gilliam R: An indirect data assimilation scheme for deep soil temperature in the Pleim–Xiu land surface model, J. Appl. Meteorol. Clim, 48, 1362–1376, 2009.

[R65] Pye HOT, Murphy BN, Xu L, Ng NL, Carlton AG, Guo H, Weber R, Vasilakos P, Appel KW, Budisulistiorini SH, Surratt JD, Nenes A, Hu W, Jimenez JL, Isaacman-VanWertz G, Misztal PK, and Goldstein AH: On the implications of aerosol liquid water and phase separation for organic aerosol mass, Atmos. Chem. Phys, 17, 343–369, 10.5194/acp-17-343-2017, 2017. 30147709 PMC6104851

[R66] Pye HOT, Nenes A, Alexander B, Ault AP, Barth MC, Clegg SL, Collett JL Jr., Fahey KM, Hennigan CJ, Herrmann H, Kanakidou M, Kelly JT, Ku I-T, McNeill VF, Riemer N, Schaefer T, Shi G, Tilgner A, Walker JT, Wang T, Weber R, Xing J, Zaveri RA, and Zuend A: The acidity of atmospheric particles and clouds, Atmos. Chem. Phys, 20, 4809–4888, 10.5194/acp-20-4809-2020, 2020. 33424953 PMC7791434

[R67] Remer LA, Kaufman YJ, Tanré D, Mattoo S, Chu DA, Martins JV, Li RR, Ichoku C, Levy RC, and Kleidman RG: The MODIS aerosol algorithm, products, and validation, J. Atmos. Sci, 62, 947–973, 2005.

[R68] Roy B, Pouliot GA, Gilliland A, Pierce T, Howard S, Bhave PV, and Benjey W: Refining fire emissions for air quality modeling with remotely sensed fire counts: A wildfire case study, Atmos. Environ, 41, 655–665, 10.1016/j.atmosenv.2006.08.037, 2007.

[R69] San Joaquin Valley Air Pollution Control District: 2018 Plan for the 1997, 2006, and 2012 PM_2.5_ Standards, available at: https://www.valleyair.org/pmplans (last access: 3 November 2021), 15 November 2018.

[R70] Sarwar G, Luecken D, Yarwood G, Whitten GZ, and Carter WPL: Impact of an updated carbon bond mechanism on predictions from the CMAQ modeling system: Preliminary assessment, J. Appl. Meteorol. Clim, 47, 3–14, 2008.

[R71] Sarwar G, Gantt B, Schwede D, Foley K, Mathur R, and Saiz-Lopez A: Impact of enhanced ozone deposition and halogen chemistry on tropospheric ozone over the Northern Hemisphere, Environ. Sci. Technol, 49, 9203–9211, 2015. 26151227 10.1021/acs.est.5b01657

[R72] Scheffe RD, Strum M, Phillips SB, Thurman J, Eyth A, Fudge S, Morris M, Palma T, and Cook R: Hybrid modeling approach to estimate exposures of hazardous air pollutants (HAPs) for the National Air Toxics Assessment (NATA), Environ. Sci. Technol, 50, 12356–12364, 10.1021/acs.est.6b04752, 2016. 27779870

[R73] Schwede D, Pouliot GA, and Pierce T: Changes to the biogenic emissions inventory system version 3 (BEIS3), in: Proceedings of the 4th CMAS Models-3 Users’ Conference, Chapel Hill, NC, 26–28 September 2005.

[R74] Sekiguchi A, Shimadera H, and Kondo A: Impact of aerosol direct effect on wintertime PM_2.5_ simulated by an online coupled meteorology-air quality model over East Asia, Aerosol. Air Qual. Res, 18, 1068–1079, 2018.

[R75] Solazzo E, Hogrefe C, Colette A, Garcia-Vivanco M, and Galmarini S: Advanced error diagnostics of the CMAQ and Chimere modelling systems within the AQMEII3 model evaluation framework, Atmos. Chem. Phys, 17, 10435–10465, 10.5194/acp-17-10435-2017, 2017. 30147711 PMC6104839

[R76] Stavrakou T, Müller J-F, De Smedt I, Van Roozendael M, van der Werf GR, Giglio L, and Guenther A: Global emissions of non-methane hydrocarbons deduced from SCIAMACHY formaldehyde columns through 2003–2006, Atmos. Chem. Phys, 9, 3663–3679, 10.5194/acp-9-3663-2009, 2009.

[R77] TEMIS (Tropospheric Emission Monitoring Internet Service): Tropospheric NO2 from satellites, available at: https://www.temis.nl/airpollution/no2.php, last access: 3 November 2021a.

[R78] TEMIS (Tropospheric Emission Monitoring Internet Service): Tropospheric ozone column, available at: https://www.temis.nl/protocols/tropo.php, last access: 3 November 2021b.

[R79] U.S. EPA: Our nation’s air status and trends through 2010, EPA-454/R-12–001, available at: https://www.epa.gov/sites/default/files/2017-11/documents/trends_brochure_2010.pdf (last access: 3 November 2021), 2012.

[R80] U.S. EPA: Policy assessment for the review of the National Ambient Air Quality Standards for particulate matter, EPA-452/R-20–002, available at: https://www.epa.gov/sites/production/files/2020-01/documents/final_policy_assessment_for_the_review_of_the_pm_naaqs_01-2020.pdf (last access: 3 November 2021), 2020.

[R81] U.S. EPA Office of Research and Development: CMAQv5.0.2, 5.0.2, Zenodo [code], 10.5281/zenodo.1079898, 2014.

[R82] Vasilakos P, Russell A, Weber R, and Nenes A: Understanding nitrate formation in a world with less sulfate, Atmos. Chem. Phys, 18, 12765–12775, 10.5194/acp-18-12765-2018, 2018.

[R83] Wang J, Wang S, Jiang J, Ding A, Zheng M, Zhao B, Wong C-D, Zhou W, Zheng G, Wang L, Pleim J, and Hao J: Impact of aerosol–meteorology interactions on fine particle pollution during China’s severe haze episode in January 2013, Environ. Res. Lett, 9, 094002, 10.1088/1748-9326/9/9/094002, 2014.

[R84] Wang K and Zhang Y: Application, evaluation, and process analysis of U.S. EPA’s 2002 multiple-pollutant air quality modeling platform, Atmospheric and Climate Sciences, 2, 254–289, 2012.

[R85] Wang K and Zhang Y: 3-D agricultural air quality modeling: Impacts of NH_3_/H_2_S gas-phase reactions and bidirectional exchange of NH_3_, Atmos. Environ, 98, 554–570, 10.1016/j.atmosenv.2014.09.010, 2014.

[R86] Wang K, Zhang Y, Jang C, Phillips S, and Wang B: Modeling intercontinental air pollution transport over the trans-Pacific region in 2001 using the Community Multiscale Air Quality modeling system, J. Geophys. Res, 114, D04307, 10.1029/2008JD010807, 2009.

[R87] Wang K, Zhang Y, Nenes A, and Fountoukis C: Implementation of dust emission and chemistry into the Community Multiscale Air Quality modeling system and initial application to an Asian dust storm episode, Atmos. Chem. Phys, 12, 10209–10237, 10.5194/acp-12-10209-2012, 2012.

[R88] Wang K, Zhang Y, Yahya K, Wu S-Y, and Grell G: Implementation and initial application of new chemistry-aerosol options in WRF/Chem for simulating secondary organic aerosols and aerosol indirect effects for regional air quality, Atmos. Environ, 115, 716–732, 10.1016/j.atmosenv.2014.12.007, 2015a.

[R89] Wang K, Yahya K, Zhang Y, Hogrefe C, Pouliot G, Knote C, Hodzic A, Jose RS, Perez JL, Jiménez-Guerrero P, Baro R, Makar P, and Bennartz R: A multi-model assessment for the 2006 and 2010 simulations under the Air Quality Model Evaluation International Initiative (AQMEII) Phase 2 over North America: Part II. Evaluation of column variable predictions using satellite data, Atmos. Environ, 115, 587–603, 10.1016/j.atmosenv.2014.07.044, 2015b.

[R90] Wang K, Zhang Y, and Yahya K: Decadal application of WRF/Chem over the continental U.S.: Simulation design, sensitivity simulations, and climatological model evaluation, Atmos. Environ, 253, 118331, 10.1016/j.atmosenv.2021.118331, 2021.

[R91] West JJ, Ansari AS, and Pandis SN: Marginal PM_2.5_: Non-linear aerosol mass response to sulfate reductions in the Eastern United States, J. Air Waste Manage. Assoc, 49, 1415–1424, 10.1080/10473289.1999.10463973, 1999. 28060636

[R92] Wiedinmyer C, Quayle B, Geron C, Belote A, McKenzie D, Zhang X, O’Neill S, and Wynne KK: Estimating emissions from fires in North America for air quality modeling, Atmos. Environ, 40, 3419–3432, 10.1016/j.atmosenv.2006.02.010, 2006.

[R93] Wielicki BA, Barkstrom BR, Harrison EF, Lee III RB, Smith GL, and Cooper JE: Clouds and the Earth’s Radiant Energy System (CERES): An earth observing system experiment, B. Am. Meteorol. Soc, 77, 853–868, 1996.

[R94] Wilczak JM, Djalalova I, McKeen S, Bianco L, Bao J-W, Grell G, Peckham S, Mathur R, McQueen J, and Lee P: Analysis of regional meteorology and surface ozone during the TexAQS II field program and an evaluation of the NMM-CMAQ and WRF-Chem air quality models, J. Geophys. Res, 114, D00F14, 10.1029/2008JD011675, 2009.

[R95] Wong DC, Pleim J, Mathur R, Binkowski F, Otte T, Gilliam R, Pouliot G, Xiu A, Young JO, and Kang D: WRF-CMAQ two-way coupled system with aerosol feedback: software development and preliminary results, Geosci. Model Dev, 5, 299–312, 10.5194/gmd-5-299-2012, 2012.

[R96] Xing J, Mathur R, Pleim J, Hogrefe C, Gan C-M, Wong DC, Wei C, and Wang J: Air pollution and climate response to aerosol direct radiative effects: A modeling study of decadal trends across the northern hemisphere, J. Geophys. Res.-Atmos, 120, 12221–12236, 10.1002/2015JD023933, 2015a.

[R97] Xing J, Mathur R, Pleim J, Hogrefe C, Gan C-M, Wong DC, and Wei C: Can a coupled meteorology–chemistry model reproduce the historical trend in aerosol direct radiative effects over the Northern Hemisphere?, Atmos. Chem. Phys, 15, 9997–10018, 10.5194/acp-15-9997-2015, 2015b.

[R98] Xing J, Wang J, Mathur R, Pleim J, Wang S, Hogrefe C, Gan C-M, Wong D, and Hao J: Unexpected benefits of reducing aerosol cooling effects, Environ. Sci. Technol, 50, 7527–7534, 10.1021/acs.est.6b00767, 2016. 27310144

[R99] Xing J, Wang J, Mathur R, Wang S, Sarwar G, Pleim J, Hogrefe C, Zhang Y, Jiang J, Wong DC, and Hao J: Impacts of aerosol direct effects on tropospheric ozone through changes in atmospheric dynamics and photolysis rates, Atmos. Chem. Phys, 17, 9869–9883, 10.5194/acp-17-9869-2017, 2017. 30147710 PMC6104653

[R100] Xiu A and Pleim JE: Development of a land surface model. Part I: Application in a mesoscale meteorological model, J. Appl. Meteorol, 40, 192–209, 10.1175/1520-0450(2001)040<0192:DOALSM>2.0.CO;2, 2001.

[R101] Yahya K, Wang K, Gudoshava M, Glotfelty T, and Zhang Y: Application of WRF/Chem over North America under the AQMEII Phase 2. Part I. Comprehensive evaluation of 2006 simulation, Atmos. Environ, 115, 733–755, 10.1016/j.atmosenv.2014.08.063, 2015a.

[R102] Yahya K, Wang K, Zhang Y, and Kleindienst TE: Application of WRF/Chem over North America under the AQMEII Phase 2 – Part 2: Evaluation of 2010 application and responses of air quality and meteorology–chemistry interactions to changes in emissions and meteorology from 2006 to 2010, Geosci. Model Dev, 8, 2095–2117, 10.5194/gmd-8-2095-2015, 2015b.

[R103] Yahya K, Wang K, Campbell P, Glotfelty T, He J, and Zhang Y: Decadal evaluation of regional climate, air quality, and their interactions over the continental US and their interactions using WRF/Chem version 3.6.1, Geosci. Model Dev, 9, 671–695, 10.5194/gmd-9-671-2016, 2016.

[R104] Yarwood G, Rao S, Yocke M, and Whitten GZ: Final Report–Updates to the Carbon Bond Chemical Mechanism: CB05, Rep. RT-04–00675, Yocke and Co., Novato, Calif, 246 pp., 2005.

[R105] Yoo J-W, Jeon W, Park S-Y, Park C, Jung J, Lee S-H, and Lee HW: Investigating the regional difference of aerosol feedback effects over South Korea using the WRF-CMAQ two-way coupled modeling system, Atmos. Environ, 218, 116968, 10.1016/j.atmosenv.2019.116968, 2019.

[R106] Yu S, Eder B, Dennis R, Chu S, and Schwartz S: New unbiased symmetric metrics for evaluation of air quality models, Atmos. Sci. Lett, 7, 26–34, 2006.

[R107] Yu S, Mathur R, Pleim J, Wong D, Gilliam R, Alapaty K, Zhao C, and Liu X: Aerosol indirect effect on the grid-scale clouds in the two-way coupled WRF–CMAQ: model description, development, evaluation and regional analysis, Atmos. Chem. Phys, 14, 11247–11285, 10.5194/acp-14-11247-2014, 2014 (data available at: https://person.zju.edu.cn/shaocaiyu#674502, last access: 3 November 2021).

[R108] Yu S, Li P, Wang L, Wu Y, Wang S, Liu W, Zhu T, Zhang Y, Hu M, Alapaty K, Wong D, Pleim J, Mathur R, Rosenfeld D, and Seinfeld J: Mitigation of severe urban haze pollution by a precision air pollution control approach, Scientific Reports, 8, 8151, 10.1038/s41598-018-26344-1, 2018. 29802392 PMC5970218

[R109] Yu SC, Mathur R, Schere K, Kang D, Pleim J, and Otte TL: A detailed evaluation of the Eta-CMAQ forecast model performance for O_3_, its related precursors, and meteorological parameters during the 2004 ICARTT Study, J. Geophys. Res, 112, D12S14, 10.1029/2006JD007715, 2007.

[R110] Yu SC, Mathur R, Pleim J, Wong D, Carlton AG, Roselle S, and Rao ST: Simulation of the indirect radiative forcing of climate due to aerosols by the two-way coupled WRF-CMAQ over the eastern United States, in Air Pollution Modeling and its Applications, edited by: Steyn DG and Castelli ST, XXI, Springer Netherlands, Netherlands, C(96), 579–583, 2011.

[R111] Yu X-Y, Lee T, Ayres B, Kreidenweis SM, Malm W, and Collett JL: Loss of fine particle ammonium from denuded nylon filters, Atmos. Environ, 40, 4797–4807, 2006.

[R112] Zender CS, Bian H, and Newman D: Mineral Dust Entrainment and Deposition (DEAD) model: Description and 1990s dust climatology, J. Geophys. Res, 108, 4416, 10.1029/2002JD002775, 2003.

[R113] Zhang Y: Online-coupled meteorology and chemistry models: history, current status, and outlook, Atmos. Chem. Phys, 8, 2895–2932, 10.5194/acp-8-2895-2008, 2008.

[R114] Zhang Y and Wang Y: Climate-driven ground-level ozone extreme in the fall over the Southeast United States, P. Natl. Acad. Sci. USA, 113, 10025–10030, 10.1073/pnas.1602563113, 2016. PMC5018760 27551089

[R115] Zhang Y and Wang K: Project 3 – Air quality and climate modeling: Multi-model application, evaluation, intercomparison, and ensemble over the U.S., poster presentation at the Air Climate Energy (ACE) Centers Meeting, Pittsburgh, PA, 18–19 June 2019.

[R116] Zhang KM, Knipping EM, Wexler AS, Bhave PV, and Tonnesen GS: Size distribution of sea-salt emissions as a function of relative humidity, Atmos. Environ, 39, 3373–3379, 2005.

[R117] Zhang Y, Liu P, Pun B, and Seigneur C: A comprehensive performance evaluation of MM5-CMAQ for the summer 1999 Southern Oxidants Study episode, Part-I. Evaluation protocols, databases and meteorological predictions, Atmos. Environ, 40, 4825–4838, 10.1016/j.atmosenv.2005.12.043, 2006.

[R118] Zhang Y, Vijayaraghavan K, Wen X-Y, Snell HE, and Jacobson MZ: Probing into regional ozone and particulate matter pollution in the United States: 1. A 1-year CMAQ simulation and evaluation using surface and satellite data, J. Geophys. Res, 114, D22304, 10.1029/2009JD011898, 2009a.

[R119] Zhang Y, Wen X-Y, Wang K, Vijayaraghavan K, and Jacobson MZ: Probing into regional ozone and particulate matter pollution in the United States: 2. An examination of formation mechanisms through a process analysis technique and sensitivity study, J. Geophys. Res, 114, D22305, 10.1029/2009JD011900, 2009b.

[R120] Zhang Y, Wen X-Y, and Jang CJ: Simulating chemistry-aerosol-cloud-radiation-climate feedbacks over the continental US using the online-coupled Weather Research Forecasting Model with chemistry (WRF/Chem), Atmos. Environ, 44, 3568–3582, 10.1016/j.atmosenv.2010.05.056, 2010.

[R121] Zhang Y, Sartelet K, Zhu S, Wang W, Wu S-Y, Zhang X, Wang K, Tran P, Seigneur C, and Wang Z-F: Application of WRF/Chem-MADRID and WRF/Polyphemus in Europe – Part 2: Evaluation of chemical concentrations and sensitivity simulations, Atmos. Chem. Phys, 13, 6845–6875, 10.5194/acp-13-6845-2013, 2013.

[R122] Zhang Y, Chen Y, Fan J, and Leung LR: Application of an online-coupled regional climate model, WRF-CAM5, over East Asia for examination of ice nucleation schemes: Part II. Sensitivity to ice nucleation parameterizations and dust emissions, Climate, 3, 753–774, 10.3390/cli3030753, 2015a.

[R123] Zhang Y, Zhang X, Wang K, He J, Leung LR, Fan J-W, and Nenes A: Incorporating an advanced aerosol activation parameterization into WRF-CAM5: Model evaluation and parameterization intercomparison, J. Geophys. Res.-Atmos, 120, 6952–6979, 10.1002/2014JD023051, 2015b.

[R124] Zhang Y, Zhang X, Wang L, Zhang Q, Duan F, and He K: Application of WRF/Chem over East Asia: Part I. Model evaluation and intercomparison with MM5/CMAQ, Atmos. Environ, 124, 285–300, 2016a.

[R125] Zhang Y, Hong C-P, Yahya K, Li Q, Zhang Q, and He K-B: Comprehensive evaluation of multi-year real-time air quality forecasting using an online-coupled meteorology-chemistry model over southeastern United States, Atmos. Environ, 138, 162–182, 10.1016/j.atmosenv.2016.05.006, 2016b.

[R126] Zhang Y, Wang K, and He J: Multi-year application of WRF-CAM5 over East Asia-Part II: Interannual variability, trend analysis, and aerosol indirect effects, Atmos. Environ, 165, 222–239, 2017.

[R127] Zhang Y, Jena C, Wang K, Paton-Walsh C, Guérette E-A, Utembe S, Silver JD, and Keywood M: Multiscale applications of two online-coupled meteorology-chemistry models during recent field campaigns in Australia, Part I: Model description and WRF/Chem-ROMS evaluation using surface and satellite data and sensitivity to spatial grid resolutions, Atmosphere, 10, 189, 10.3390/atmos10040189, 2019.

[R128] Zheng B, Zhang Q, Zhang Y, He KB, Wang K, Zheng GJ, Duan FK, Ma YL, and Kimoto T: Heterogeneous chemistry: a mechanism missing in current models to explain secondary inorganic aerosol formation during the January 2013 haze episode in North China, Atmos. Chem. Phys, 15, 2031–2049, 10.5194/acp-15-2031-2015, 2015.

