# Supplementary material for: A comparative study of two-way and offline coupled WRF v3.4 and CMAQ v5.0.2 over the contiguous US: performance evaluation and impacts of chemistry–meteorology feedbacks on air quality
Source: Geosci Model Dev. Author manuscript; Available in PMC 2022 Nov 26. (PMC8883479; doi:10.5194/gmd-14-7189-2021)
Supplement: Supplement1 [file NIHMS1779352-supplement-Supplement1.pdf]

Supplement of Geosci. Model Dev., 14, 7189–7221, 2021
https://doi.org/10.5194/gmd-14-7189-2021-supplement
© Author(s) 2021. CC BY 4.0 License.

Geoscientific Model Development
Open Access

Supplement of

A comparative study of two-way and offline coupled WRF v3.4 and CMAQ v5.0.2 over the contiguous US: performance evaluation and impacts of chemistry–meteorology feedbacks on air quality

Kai Wang et al.

Correspondence to: Yang Zhang (ya.zhang@northeastern.edu) and Shaocai Yu (shaocaiyu@zju.edu.cn)

The copyright of individual parts of the supplement might differ from the article licence.

Table S1. The model configurations of the two-way WRF-CMAQ simulation.

**Attributes**	**Model Configurations**
Model	WRF v3.4-CMAQ v5.0.2
Simulation period	2008-2012
Domain	CONUS
Horizontal grid spacing	36-km (148 × 112 grid cells)
Vertical grid	34 layers from surface to 100 hPa
Physical options	
Shortwave radiation	Rapid and accurate Radiative Transfer Model for GCM (RRTMG)
Longwave radiation	RRTMG
PBL	ACM2
Land surface	Pleim-Xiu
Microphysics	Morrison two-moment
Cumulus	Kain-Fritsch
Aerosol activation	Abdul-Razzak and Ghan
Chemical options	
Gas-phase chemistry	CB05 with updated chlorine chemistry
Aerosol module	AERO6
Photolysis	CMAQ inline
Aqueous-phase chemistry	AQ chemistry module (AQCHEM)
Meteorological and chemical IC and BC	Downscaled from the modified Community Earth System Model/Community Atmosphere Model (CESM/CAM5) v1.2.2; Meteorological ICs/BCs bias-corrected with National Center for Environmental Protection's Final (FNL) Operational Global Analysis data
Anthropogenic emission	NEI 2008 updated to 2010, and NEI 2011
Biogenic emission	BEIS3
Dust emission	CMAQ inline
Sea-salt emission	CMAQ inline

Table S2 summarizes the observational databases and the variables evaluated in this work. For evaluation of chemical concentrations and meteorological variables, the surface networks include the National Climatic Data Center (NCDC) Quality Controlled Local Climatological Data (QCLCD), Clean Air Status and Trends Network (CASTNET), the Aerometric Information Retrieval System (AIRS) – Air Quality System (AQS), the Interagency Monitoring of Protected Visual Environments (IMPROVE), the Chemical Speciation Network (CSN), the Southeastern Aerosol Research and Characterization (SEARCH), and the National Atmospheric Deposition Network (NADP). Several aerosol-cloud-radiation variables are also evaluated against satellite retrievals including the Clouds and the Earth's Radiant Energy System (CERES) and the Moderate Resolution Imaging Spectroradiometer (MODIS).

NCDC QCLCD data contains data over 700 U.S. locations from July 1996 to December 2004, and over 1600 locations from 2005 onwards (http://www.ncdc.noaa.gov/data-access/land-based-station-data/land-based-datasets/quality-controlled-local-climatological-data-qclcd). CASTNET observations have been collected in a range of rural environments, from desert to agricultural locations, and from flat to complex terrains (http://java.epa.gov/castnet/epa_jsp/sites.jsp). It contains measurement data for meteorological variables and chemical concentrations. AIRS-AQS is the U.S. EPA's repository for ambient air quality data from over 5000 active monitors (http://www.epa.gov/ttn/airs/airsaqs/). While IMPROVE observations have been collected in protected visual environments, i.e., in National Parks and Wilderness Areas (http://vista.cira.colostate.edu/improve/), CSN sites are located in a range of locations from urban to rural areas (http://www.epa.gov/ttnamti1/specgen.html). Both networks contain data for $PM_{2.5}$ and major $PM_{2.5}$ species. NADP contains precipitation data from rain gauges.

The MODIS satellite retrievals for AOD (Remer et al., 2005), CF, COT, and CWP come from the level 3 MODIS gridded atmosphere monthly global joint product (MOD08_M3) collected from the Terra platform (http://modis-atmos.gsfc.nasa.gov/MOD08_M3/). The CDNC data used in this study are derived from MODIS by Bennartz (2007).

Table S2. Observational datasets and variables evaluated in this study.

**Gases and PM Species**			
**Observational database**	**Variables evaluated**	**Sampling Frequency**	**Number of Sites**
CASTNET	Max 1-hr and 8-hr O_3	Daily for O_3	~90
AIRS–AQS	O_3	Hourly	~1150
IMPROVE	$PM_{2.5}$, SO_4^{2-}, NO_3^-, NH_4^+, EC, OC	24-hour data. Data availability once every 3 days	~160
CSN	$PM_{2.5}$, SO_4^{2-}, NO_3^-, NH_4^+, EC, TC	24-hour data. Data availability once every 3 days	~200
**Meteorology**			
Observational Database	*Variables evaluated*	*Temporal Resolution*	*Spatial Resolution*
NCDC QCLCD	T2, RH, WS10,WD10	Hourly	~700 before 2005<br>~1600 after 2005

NADP	Precipitation	Weekly	255
**Radiation and other Aerosol/Cloud variables**			
Observational Database/ Satellite	*Variables evaluated*	*Temporal Resolution*	*Number of sites/ Spatial Resolution*
CERES	SWDOWN	Monthly	1° × 1°
MODIS	AOD, CF, COT, CWP, QVAPOR, CCN	Monthly	1° × 1°
MODIS derived based on Bennartz (2007)	CDNC	Monthly	1° × 1°

Table S3. The 5-year (2008-2012) average performance statistics for meteorological variables between two-way WRF-CMAQ and WRF-only simulations.

Variables	Datasets	Mean Obs	Two-way WRF-CMAQ					WRF-only				
			Mean Sim	R	MB	NMB (%)	RMSE	Mean Sim	R	MB	NMB (%)	RMSE
**T2 (^oC)**	**NCDC**	12.9	13.0	0.98	0.1	0.8	1.0	13.1	0.98	0.2	1.8	1.1
**RH2 (%)**		69.1	71.3	0.88	2.2	3.2	5.3	71.0	0.88	1.8	2.6	5.2
**WS10 (m s^{-1})**		3.74	4.18	0.52	0.44	11.7	1.15	4.20	0.52	0.46	12.4	1.16
**WD10 (deg)**		154.4	187.2	0.07	32.8	21.3	47.7	187.8	0.06	33.4	21.6	48.1
**Precipitation (mm day^{-1})**	**NCDC**	1.84	2.55	0.62	0.71	38.4	1.27	2.64	0.62	0.8	43.5	1.33
	**NADP**	2.66	2.81	0.84	0.15	5.8	0.7	2.9	0.84	0.24	9.3	0.73
	**GPCP**	2.15	2.43	0.79	0.28	13.0	0.9	2.45	0.80	0.30	14.1	0.9
	**PRISM**	2.16	2.30	0.91	0.14	6.8	0.55	2.36	0.91	0.20	9.5	0.56
	**TMPA**	2.28	2.50	0.86	0.22	9.9	0.81	2.52	0.86	0.24	10.7	0.82
**SWDOWN (W m^{-2})**	**CERES**	185.6	209.8	0.97	24.2	13.0	25.7	222.6	0.96	37.0	19.9	38.3
**GSW (W m^{-2})**		158.5	176.0	0.97	17.6	11.1	19.8	187.0	0.95	28.5	18.0	30.6
**GLW (W m^{-2})**		322.9	316.8	0.99	-6.1	-1.9	8.1	312.3	0.99	-10.6	-3.3	12.1
**OLR (W m^{-2})**		241.2	243.2	0.99	2.0	0.8	3.5	244.0	0.99	2.8	1.2	4.2
**SWCF (W m^{-2})**		-41.1	-30.4	0.74	-10.7	-26.0	13.7	-23.5	0.63	-17.6	-42.8	20.1
**LWCF (W m^{-2})**		23.7	18.4	0.73	-5.3	-22.2	6.5	17.8	0.74	-5.9	-24.9	6.9
**AOD**	**MODIS**	0.15	0.05	0.60	-0.1	-64.8	0.11	N/A	N/A	N/A	N/A	N/A
**CF**		0.57	0.50	0.92	-0.07	-12.2	0.09	N/A	N/A	N/A	N/A	N/A
**CDNC (cm^{-3})**		163.3	29.3	0.35	-134.0	-82.1	138.8	N/A	N/A	N/A	N/A	N/A
**CWP (g m^{-2})**		167.4	81.6	0.79	-85.8	-51.2	90.4	N/A	N/A	N/A	N/A	N/A
**COT**		15.3	3.0	0.84	-12.3	-80.1	12.6	N/A	N/A	N/A	N/A	N/A

*outputs of AOD, CF, CDNC, CWP, and COT are not available from WRF-only simulations

Table S4. The 5-year (2008-2012) average performance statistics for chemical variables between two-way WRF-CMAQ and offline CMAQ simulations.

Variables	Datasets	Mean Obs	Two-way WRF-CMAQ					Offline CMAQ				
			Mean Sim	R	MB	NMB (%)	NME (%)	Mean Sim	R	MB	NMB (%)	NME (%)
Max 8-hr O_3 (ppb)	AQS	43.5	49.0	0.66	5.5	12.6	13.1	51.2	0.66	7.7	17.7	17.9
	CASTNET	42.2	42.8	0.65	0.6	1.5	8.4	45.1	0.65	3.0	7.0	10.5
$PM_{2.5}$ (μg m^{-3})	CSN	10.7	9.9	0.50	-0.75	-7.0	21.9	10.3	0.46	-0.36	-3.4	21.7
	IMPROVE	4.78	4.13	0.88	-0.65	-13.7	26.6	4.51	0.87	-0.27	-5.7	23.2
PM_{10} (μg m^{-3})	AQS	24.0	13.0	0.02	-11.0	-45.9	49.6	15.4	0.14	-8.6	-35.6	45.0
SO_4^{2-} (μg m^{-3})	CSN	2.32	1.70	0.88	-0.62	-26.7	27.1	1.57	0.89	-0.75	-32.3	32.3
	IMPROVE	1.08	0.78	0.98	-0.29	-27.2	27.2	0.76	0.98	-0.32	-29.4	29.4
NO_3^- (μg m^{-3})	CSN	1.29	1.51	0.85	0.22	16.6	32.8	1.73	0.85	0.43	33.5	44.9
	IMPROVE	0.41	0.47	0.85	0.06	14.6	42.9	0.57	0.87	0.16	39.0	51.7
NH_4^+ (μg m^{-3})	CSN	1.03	0.88	0.86	-0.15	-14.3	18.6	0.87	0.85	-0.16	-15.7	18.7
EC (μg m^{-3})	CSN	0.63	0.76	0.34	0.13	20.6	52.4	0.77	0.39	0.14	22.4	50.5
	IMPROVE	0.18	0.23	0.80	0.05	29.4	50.8	0.25	0.79	0.07	37.7	55.6
OC (μg m^{-3})	IMPROVE	0.97	0.69	0.59	-0.28	-28.9	44.8	0.74	0.58	-0.23	-23.8	43.4
TC (μg m^{-3})	CSN	2.87	2.60	0.10	-0.27	-9.4	29.7	2.71	0.07	-0.16	-5.7	28.8
	IMPROVE	0.68	0.62	0.79	-0.06	-9.2	37.2	0.80	0.72	-0.08	-9.2	39.0
Col. CO (10^{18} mole. cm^{-3})	MOPITT	1.96	1.44	0.89	-0.52	-26.6	26.7	1.45	0.89	-0.51	-26.2	26.2
TOR (DU)	OMI	30.3	30.8	0.83	0.47	1.6	4.7	31.1	0.82	0.77	2.5	5.1
Col. NO_2 (10^{15} mole. cm^{-3})	SCIAMACHY	1.27	1.09	0.91	-0.18	-14.5	27.1	1.08	0.91	-0.19	-14.9	27.3
Col. HCHO (10^{15} mole. cm^{-3})	SCIAMACHY	5.13	4.21	0.83	-0.92	-18.0	20.6	4.28	0.83	-0.85	-16.6	19.8

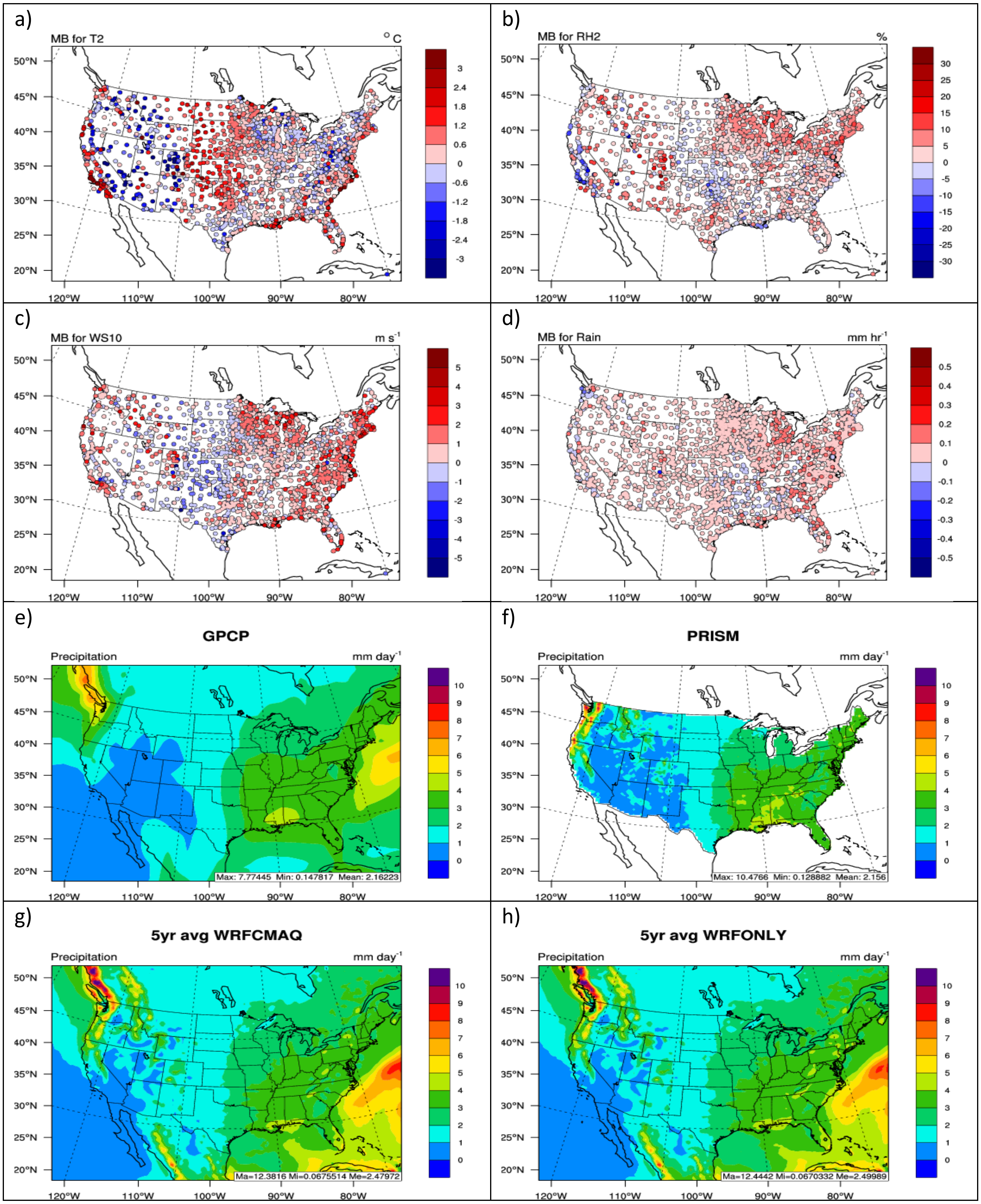

Figure S1. Spatial distributions of 5-year average MBs for a) 2-m temperature (T2), b) 2-m relative humidity (RH2), c) 10-m wind speed (WS10), and d) hourly precipitation from NCDC for two-way WRF-CMAQ in 2008-2012 and 5-year average of daily precipitation for e) GPCP, f) PRISM, g) two-way WRF-CMAQ, and h) WRF-only.

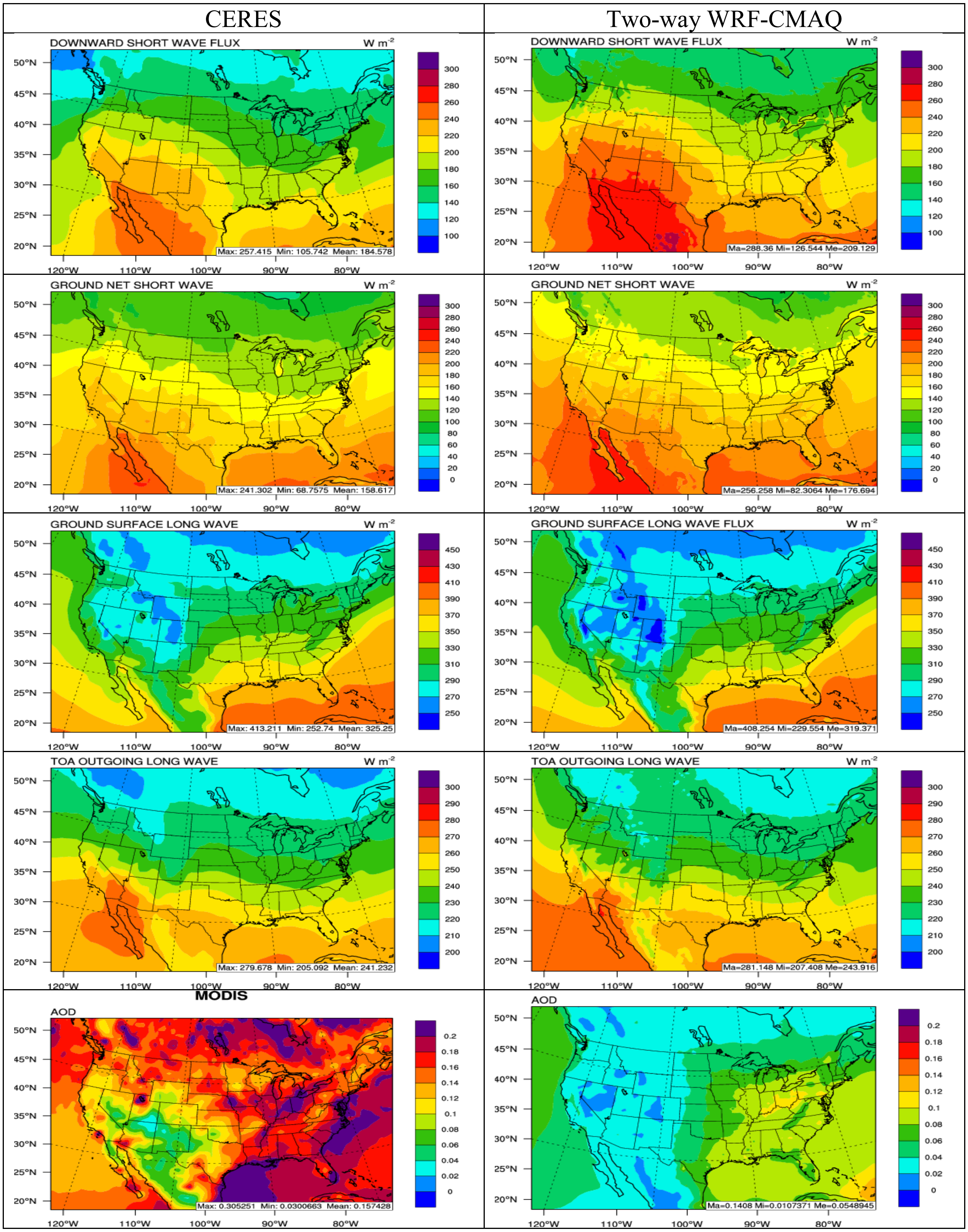

Figure S2. Spatial distribution of 5-year average major radiation variables (from top to bottom: SWDOWN, GSW, GLW, OLR, and AOD) between CERES observations (left panel) vs. two-way WRF-CMAQ (right panel) for 2008-2012.

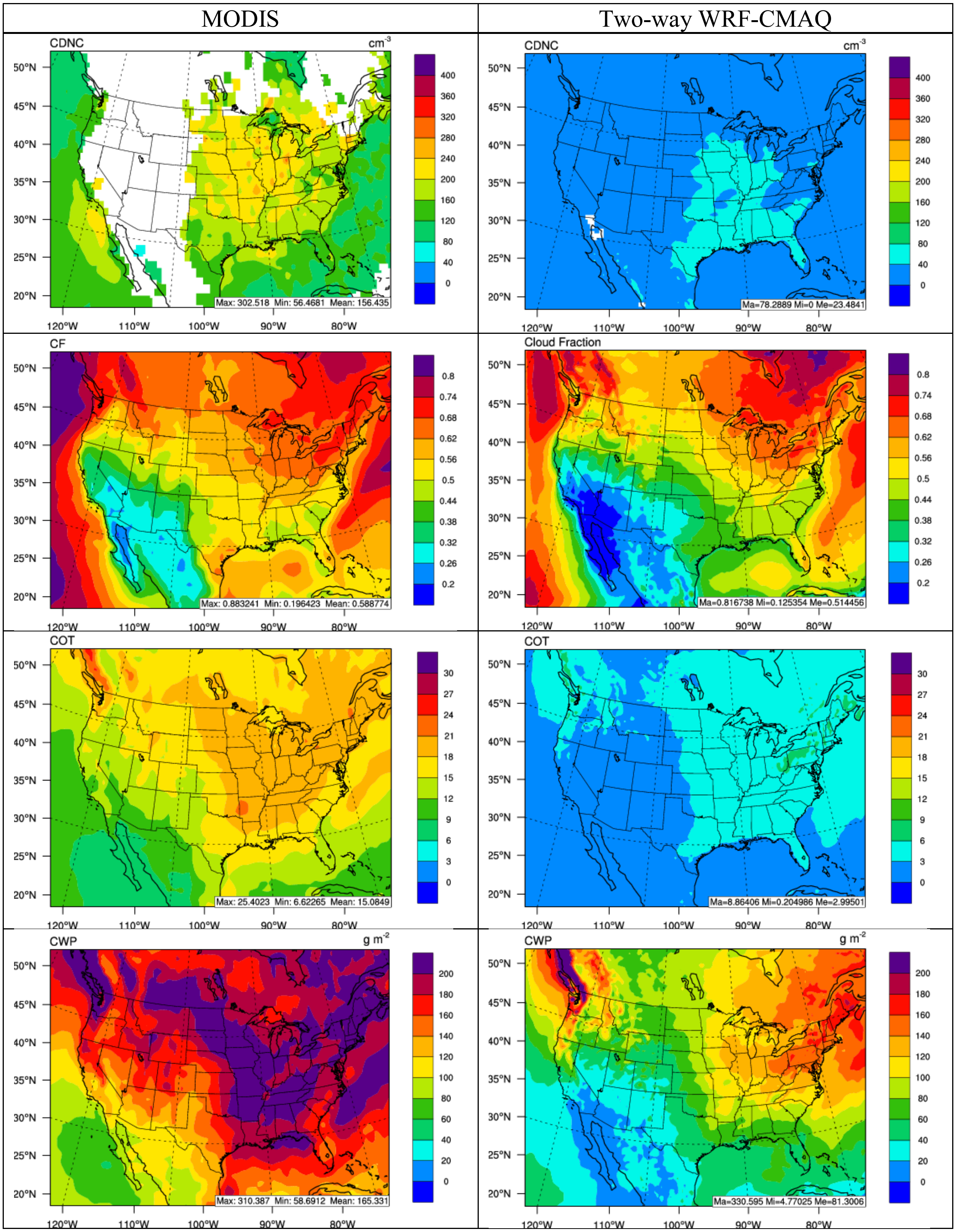

Figure S3. Spatial distribution of 5-year average major cloud variables (from top to bottom: CDNC, CF, COT, and CWP) between MODIS observations (left panel) vs. two-way WRF-CMAQ (right panel) for 2008-2012.

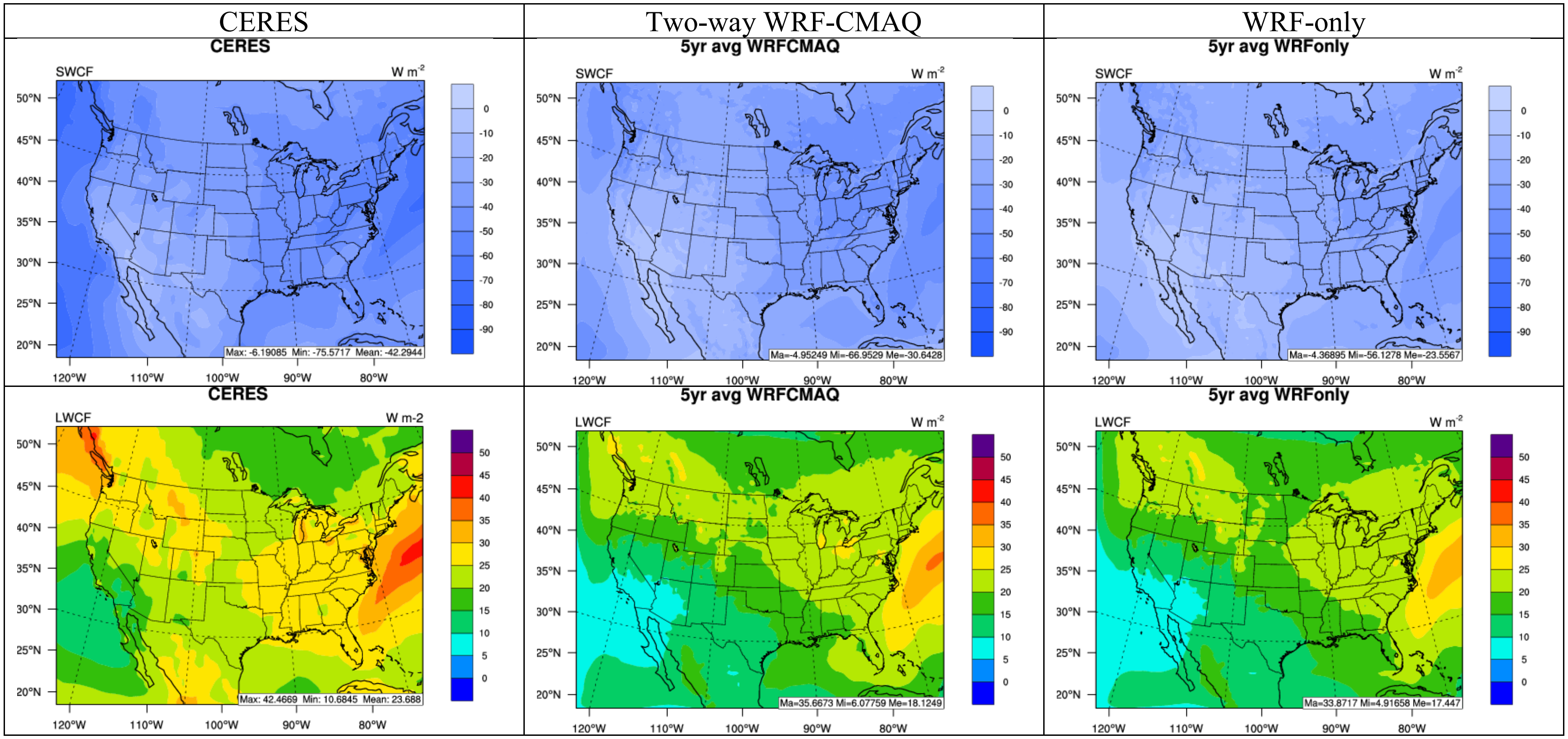

Figure S4. Spatial distribution of 5-year average SWCF (top panel) and LWCF (bottom panel) between SERES observations (left panel) vs. two-way WRF-CMAQ (center panel) and WRF-only (right panel) for 2008-2012.

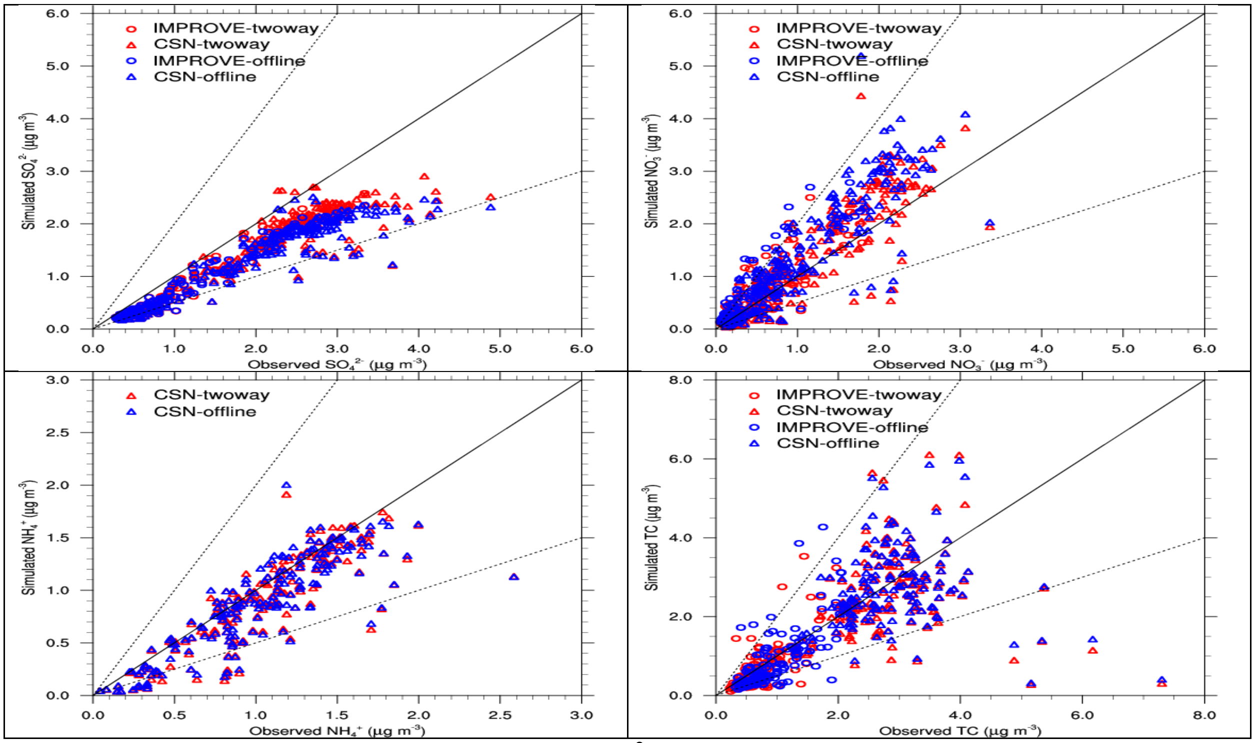

Figure S5. scatter plots of $PM_{2.5}$ constituents SO_4^{2-}, NH_4^+, NO_3^-, and TC between observations and simulations of two-way WRF-CMAQ (red color) and offline CMAQ (blue) for 2008-2012.

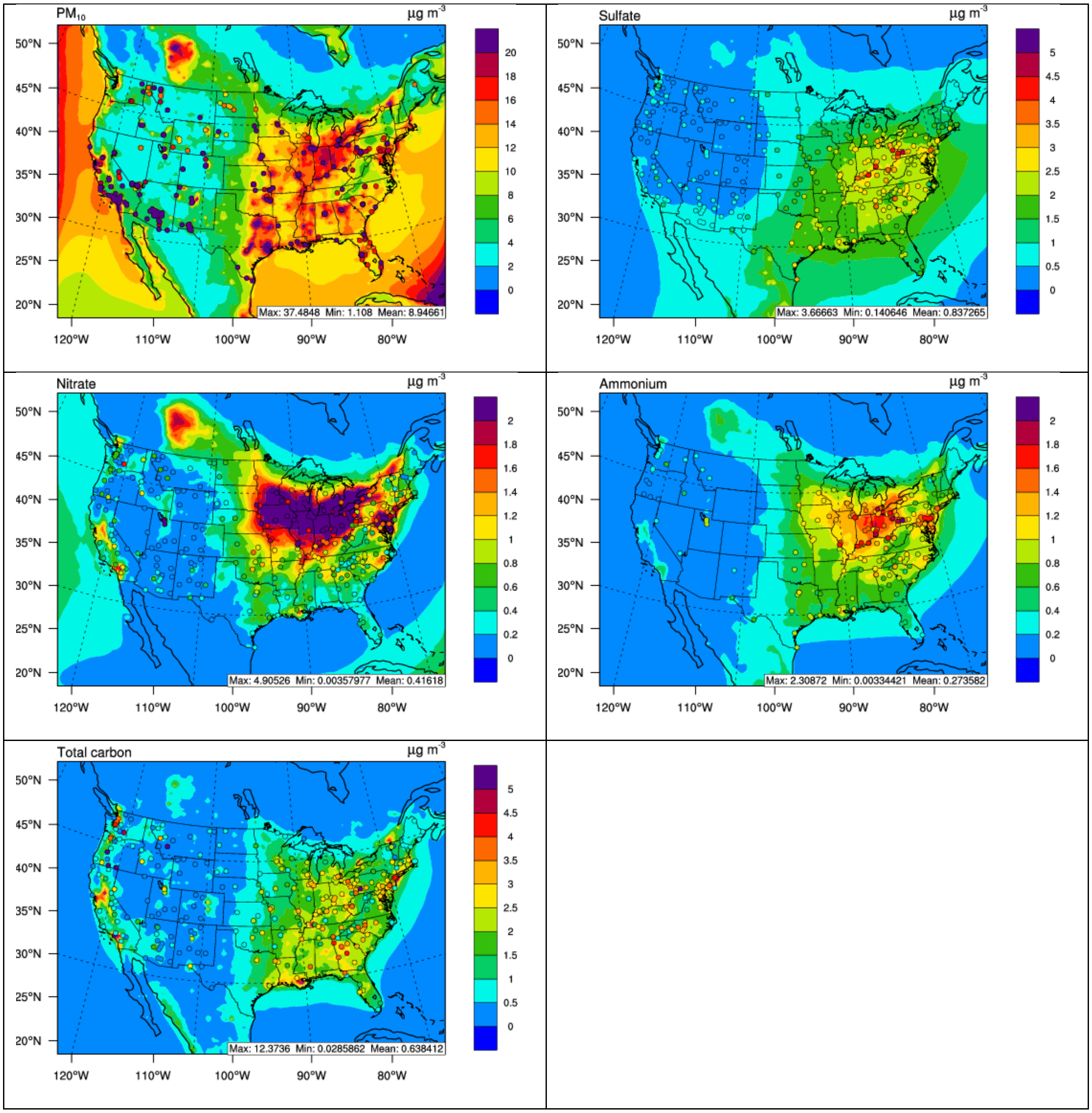

Figure S6. Spatial distributions of 5-year averaged daily PM_{10} and $PM_{2.5}$ constituents overlaid with observations.

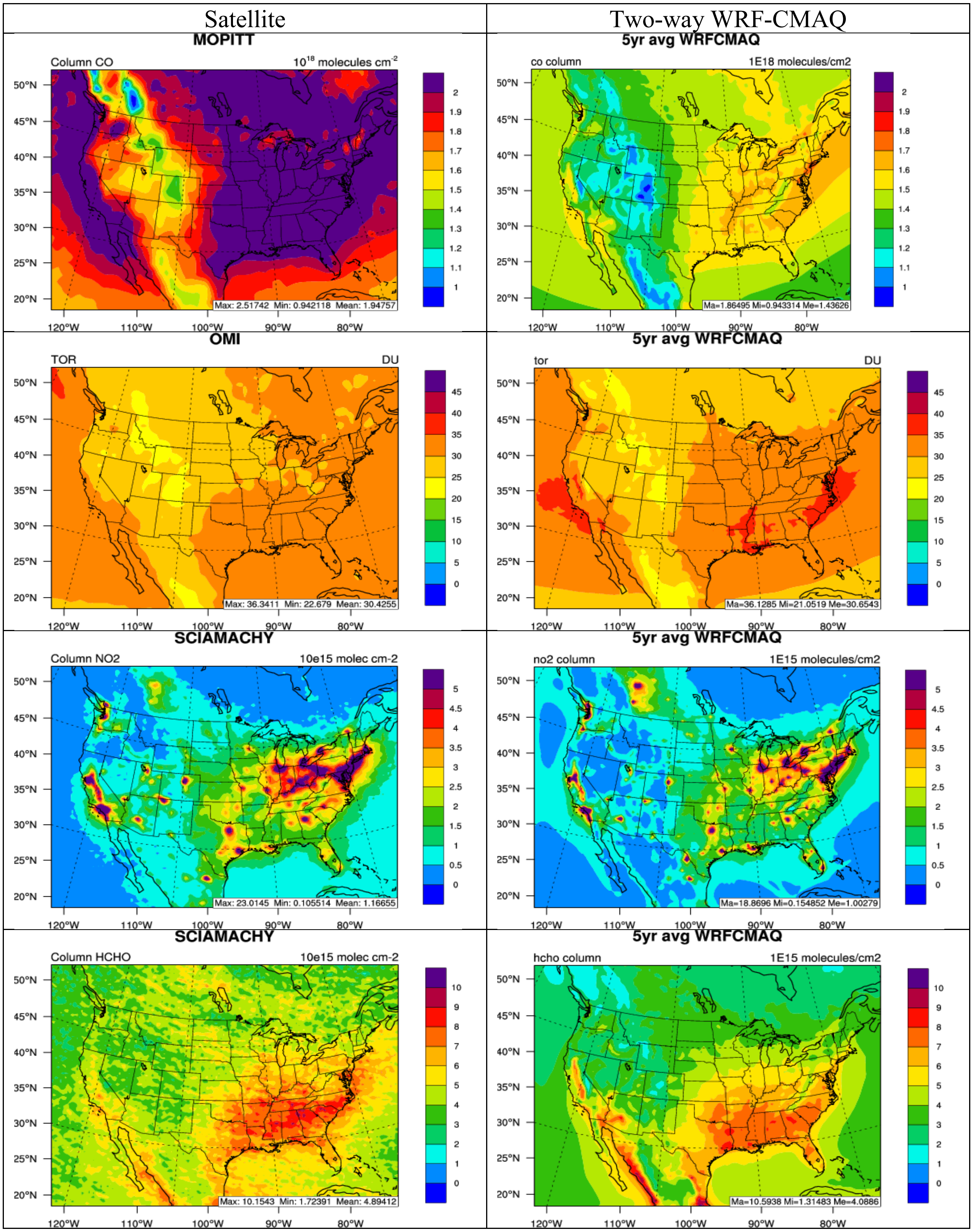

Figure S7. Spatial distribution of 5-year average column abundances (from top to bottom: column CO, TOR, column NO_2, and column HCHO) between various satellite observations (left panel) vs. two-way WRF-CMAQ (right panel) for 2008-2012.
